# The repetitive DNA landscape in Avena (Poaceae): chromosome and genome evolution defined by major repeat classes in whole-genome sequence reads

**DOI:** 10.1186/s12870-019-1769-z

**Published:** 2019-05-30

**Authors:** Qing Liu, Xiaoyu Li, Xiangying Zhou, Mingzhi Li, Fengjiao Zhang, Trude Schwarzacher, John Seymour Heslop-Harrison

**Affiliations:** 1 Key Laboratory of Plant Resources Conservation and Sustainable Utilization / Guangdong Provincial Key Laboratory of Applied Botany, South China Botanical Garden, Chinese Academy of Sciences, Guangzhou, China; 2 University of Chinese Academy of Sciences, Beijing, China; 3 Genepioneer Biotechnologies Co. Ltd., Nanjing, China; 4 Institute of Botany, Jiangsu Province and Chinese Academy of Sciences, Nanjing, China; 5 Department of Genetics and Genome Biology, University of Leicester, Leicester, LE1 7RH UK

**Keywords:** Chromosome evolution, Common oat (*Avena sativa*), Fluorescence in situ hybridization (FISH)-based karyotypes, Genome-specific markers, Intergenomic translocations, Repetitive DNAs

## Abstract

**Background:**

Repetitive DNA motifs – not coding genetic information and repeated millions to hundreds of times – make up the majority of many genomes. Here, we identify the nature, abundance and organization of all the repetitive DNA families in oats (*Avena sativa*, 2*n* = 6*x* = 42, AACCDD), a recognized health-food, and its wild relatives.

**Results:**

Whole-genome sequencing followed by k-mer and RepeatExplorer graph-based clustering analyses enabled assessment of repetitive DNA composition in common oat and its wild relatives’ genomes. Fluorescence in situ hybridization (FISH)-based karyotypes are developed to understand chromosome and repetitive sequence evolution of common oat. We show that some 200 repeated DNA motifs make up 70% of the *Avena* genome, with less than 20 families making up 20% of the total. Retroelements represent the major component, with Ty3/Gypsy elements representing more than 40% of all the DNA, nearly three times more abundant than Ty1/Copia elements. DNA transposons are about 5% of the total, while tandemly repeated, satellite DNA sequences fit into 55 families and represent about 2% of the genome. The *Avena* species are monophyletic, but both bioinformatic comparisons of repeats in the different genomes, and in situ hybridization to metaphase chromosomes from the hexaploid species, shows that some repeat families are specific to individual genomes, or the A and D genomes together. Notably, there are terminal regions of many chromosomes showing different repeat families from the rest of the chromosome, suggesting presence of translocations between the genomes.

**Conclusions:**

The relatively small number of repeat families shows there are evolutionary constraints on their nature and amplification, with mechanisms leading to homogenization, while repeat characterization is useful in providing genome markers and to assist with future assemblies of this large genome (c. 4100 Mb in the diploid). The frequency of inter-genomic translocations suggests optimum strategies to exploit genetic variation from diploid oats for improvement of the hexaploid may differ from those used widely in bread wheat.

**Electronic supplementary material:**

The online version of this article (10.1186/s12870-019-1769-z) contains supplementary material, which is available to authorized users.

## Background

Genome evolution involves multiple processes including whole genome duplications (WGDs or polyploidy), segmental genome deletions or duplications, chromosome restructuring (fusion, fission, translocation, and inversion), and amplification or loss of gene and repetitive sequences, along with DNA mutation [1, 2]. There is a growing interest in reconstructing ancestral genomes of fungi [3], animals [4] and plants [5], revealing principles governing genome evolution and diversification leading to speciation and adaptation.

Repetitive DNA constitutes a substantial fraction, typically between 25 and 85%, of plant genomes, and can be referred to as the repeatome. Repeat motifs vary extensively in sequence and dispersion patterns [6–8]. Several major groups of repetitive elements are found: ribosomal DNAs (rDNAs) [both 45S (18S–5.8S-26S) and 5S rDNAs with intergenic spacers], the telomeric repeats, class I retrotransposons (amplified through an RNA intermediate), class II DNA transposons (amplified through DNA copies), and tandem repeats (postulated to be generated/modified by slippage replication, uneven crossing-over or rolling circle amplification) [7]. Their presence and similarity, variation in copy number and sequences, pose a major challenge to genome assembly and gene annotation [9]. Repetitive DNA has been postulated to have multiple roles in the genome, including genome stability, recombination, chromatin modulation and modification of gene expression [7]. Copy number variations in repeats, representing 5 to 10% of the human genome, are important for disease and population variation [10].

Through the decades up to 2010, repeatome knowledge came largely from DNA annealing experiments, screens of random clones, restriction fragment analyses, or amplification of conserved elements with primers. Now whole-genome shotgun sequencing approaches can be used for genome-wide, unbiased repeat analysis [11–13]. A k-mer analysis counts the number of motifs k-bases long in whole-genome sequence reads [14], to identify abundant motifs without using reference genomes. The graph-based clustering analysis (e.g. RepeatExplorer [11, 15]) is another approach to identify and classify repeats from raw reads. Both are de novo identification strategies, and results can be used for repeat identification or protein domain searches. Because of the multiple genomic locations and difficulties of assembly, in situ hybridization to chromosomal preparation is essential to identify the genomic locations and specificity of repetitive motifs [16]. These approaches have been used to quantify the genome repetitive landscape in banana, radish, soybean and tobacco [17–21].

Common oat (*Avena sativa* L., 2*n* = 6*x* = 42, AACCDD) is a temperate crop (annual production of 23 million tons in 2017; http://faostat.fao.org) with the approved health claim as a ‘superfood’ because of oat beta-glucan, which helps reduce blood cholesterol level and heart disease risk [22, 23]. Genomic resource development of common oat, important for breeding and improvement, has lagged behind other major crops [24, 25]. There are several genetic maps of diploid and hexaploid species [26, 27] but no draft genome sequences for the hexaploid crop (6*x* genome size 12,600 Mb/1C) [28] or its diploid relatives. The oat genome contains numerous families of repeats and apparently frequent chromosome translocations [29, 30]. In recent phylogenetic analyses, common oat was inferred to experience ancient allotetraploidy and recent allohexaploidy events involving C-, A- and D-genome ancestors [31, 32], while the genome reshuffling obscures contributions of different candidate maternal A-genome progenitors (bipaternal genome definition referred to [32]).

Here, we aimed to elucidate structure, organization, and relationship of all major repetitive DNA classes in diploid and hexaploid oats, examine their chromosomal locations, and understand the significance of repeatome in genome and chromosome evolution of *Avena* in the context of genomic, bioinformatic and cytogenetic evidence. The complete picture of repetitive DNAs provides new evidence for events occurring during evolution and speciation in the genus, including hybridization and chromosomal translocation events.

## Results

### Graph-based clustering and repeat composition of *Avena*

Raw sequence reads (Illumina 250 bp paired end) obtained from *Avena sativa*, *A. brevis*, *A. hirtula*, and *A. strigosa* averaged 43.23% GC (guanine-cytosine) content (Additional file 1: Figure S1, Additional file 13: Tables S1, Additional file 14: Table S2a). For graph-based clustering of reads, a 1.72 to 2.87 Gb subset were analysed using RepeatExplorer [11] (Additional file 14: Table S2b). In total, more than 70% of reads were assigned into just 200 graph clusters of highly related sequence reads (Additional file 2: Figure S2, Table 1), with 12 to 18 clusters (depending on species) representing more than 1% of all the reads (Additional file 15: Table S3b).

**Table 1:** Repetitive DNA percentage composition by RepeatExplorer analyses of four *Avena* species genomes

Species	Number of clusters representing > 0.01% of genome	Genome percentage in > 0.01% clusters	Genome % in top 15 clusters	Number of clusters > 1% of genome	% of genome annotated as Gypsy	Copia	LINEs non-LTR retroelements	DNA trans-posons	EnSpm	GC% of > 0.01% clusters
*A. sativa*	214	71.0%	17.8%	12	43.1%	16.0%	0.32%	5.5%	4.0%	44.0%
*A. brevis*	214	71.6%	19.5%	16	42.8%	15.9%	0.32%	5.4%	4.0%	43.3%
*A. hirtula*	198	72.5%	21.2%	14	40.5%	17.3%	0.45%	5.9%	5.0%	44.2%
*A. strigosa*	195	71.6%	23.7%	18	40.8%	17.4%	0.42%	5.9%	5.2%	44.0%

**Fig. 1:**
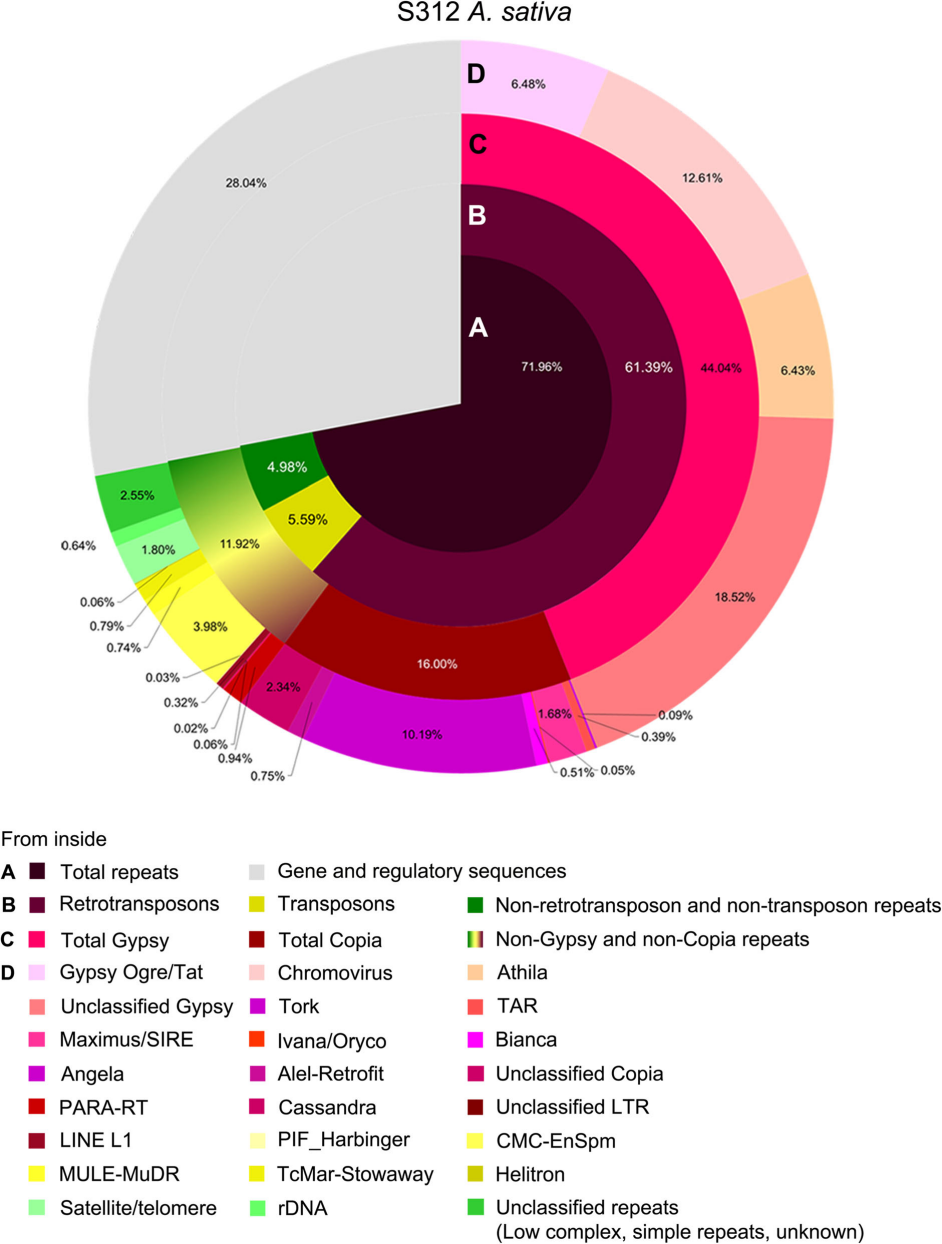
Frequency of major repetitive DNA classes in *Avena sativa.* Repeats were identified by graph-based clustering (RepeatExplorer) and in abundant k-mer motifs, and classified by nucleotide domain hits and database Blast searches. Concentric circles show increasing information from center; results were similar for diploid genomes (Additional file 17: Table S5)

After manual verification by checking domain homology or satellite motifs (Additional file 16: Table S4), 65% of *Avena* genome reads were related to transposable elements [TEs, including class I retrotransposons (average 60.80%) and class II DNA transposons (average 5.78%)], and tandem repeats [satellite DNA (satDNA), rDNA, and telomere; average 2.70%] (Table 1, Additional file 17: Table S5 [33]). Long terminal repeat (LTR) retrotransposons dominated in class I retrotransposons, with Ty3-Gypsy (Metaviridae; average 42.8%; gene order reverse transcriptase-RnaseH-integrase, RT-RH-INT) and Ty1-Copia (Pseudoviridae; average 16.6%; gene order INT-RT-RH) superfamilies, while non-LTR retrotransposons including long interspersed nuclear elements (LINEs) represented less than 0.5%. Class II DNA transposons, including Helitrons, included up to 5.2% enhancer suppressor mutator (EnSpm) elements. There were no notable differences of repetitive DNA composition among four *Avena* genomes (Additional file 17: Table S5). The clustering analysis groups solo-LTRs and SINEs with their parental elements; cluster graphs showed the greater abundance of LTRs compared to the coding sequences (Fig. 1h). The Blast results identified less than 20 chloroplast sequence clusters (abundance ranking 46–168, removed from further analysis), and about 11 rDNA clusters (Additional file 16: Table S4). Only a small proportion (2.44–4.49%) of clusters were unclassified (Additional file 17: Table S5), showing little similarity to characterized sequences, but some had adenine-thymine-rich domains (Additional file 18: Table S6 [34, 35]). Our analyses were not designed to identify most microsatellite arrays (including telomeric sequences), typically shorter than 10-mers.

**Fig. 2:**
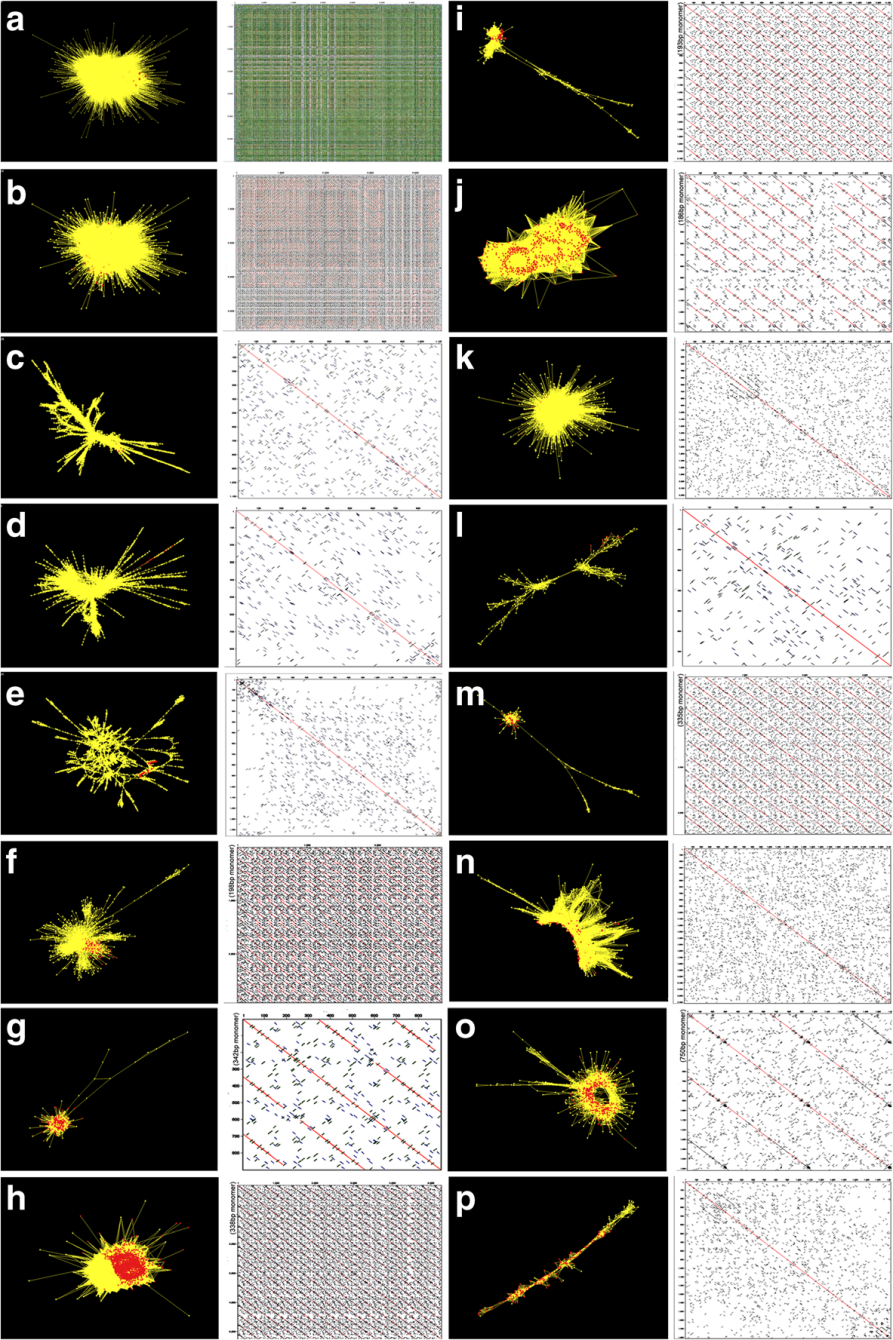
Repeat cluster graphs and dotplots of selected repeats in *Avena* species. For each repeat, the RepeatExplorer cluster is shown (left; yellow nodes represent all assembled contigs within the repeat cluster; red nodes represent members of the contig analysed in greater detail, including by amplification and in situ hybridization. A self-dotplot of the selected contig is shown in the right panel; parallel diagonals show tandem repeats. **a** As_16mer43bp, identified in cluster 289CL8C154. **b** AF226603_45bp in cluster 289CL8C248. **c** Cluster Ab-R18. **d** Ab-R19. **e** Ast-R87. **f** Ab-T145. **g** Ah-T118. **h** As-T119. **i** As-T153. **j** As-T175. **k** pAs120a in cluster 312CL17C141. **l** Ast-R171. **m** Ast-T116. **n** Ah-R31. **o** Ab-T159. **p** Ast-R176. Repeat names include species origin of the exemplar family member: Ab, *Avena brevis*; Ah, *A. hirtula*; Ast, *A. strigosa*; As, *A. sativa* and repeat type: T, tandem; R, retrotransposon

Clusters showed characteristic graph patterns (Fig. 2a-p) that we used to classify the repeat families. As examples, 0.15% of reads formed the star-shape cluster graph for the tandemly repeated satellite DNA As-T119 (Fig. 2h), 0.77% of reads formed the ray-shape retrotransposon repeat Ah-R31 (Fig. 2n), 0.04% of reads formed the circular-shape repeat Ab-T159 (Fig. 2o), and 0.02% of reads forming line-shape simple repeat Ast-R176 (Additional file 19: Table S7). Eight of fragments used for in situ hybridization had 335-360 bp monomers (Additional file 20: Table S8a [36–38]). 312CL151C2 (no PCR products without designation, Additional file 20: Table S8b) was unique as it showed a higher order repeat structure of 232 bp dimer consisting of two closely related 116 bp monomers.

### k-mer analyses of *Avena*

**Fig. 3:**
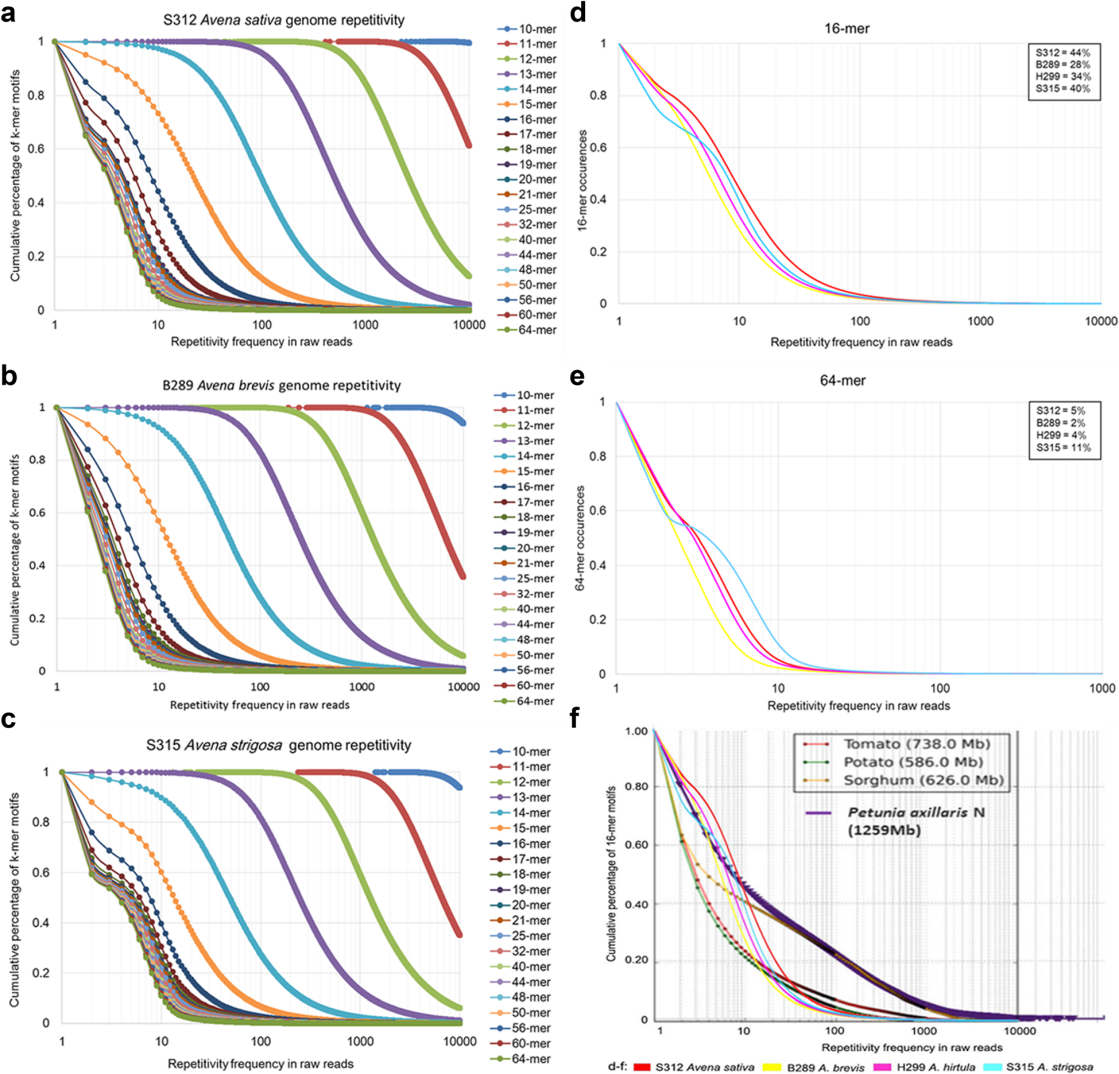
K-mer repetitivity frequencies in *Avena* genome raw reads. Cumulative percentages of k-mer motifs are plotted against frequencies of different k-mers in *Avena sativa* (**a**), *A. brevis* (**b**) and *A. strigosa* (**c**). Comparison of 16-mer (**d**) and 64-mer (**e**) frequencies in four *Avena* genomes. Inset box gives percentages of k-mer occurrence with ≥10 per genome equivalent. Cumulative 16-mer frequency (**f**) of *Avena* genomes in comparison with *Petunia axillaris* (Bombarely et al. [21]; their Additional file 10: their Figure S10), potato (Xu et al. [40]), sorghum (Paterson et al. [39]), and tomato (TGC [41]; their Supplementary Fig S42)

For cumulative repetitivity frequency plots of 10- to 64-mers, the steeper slope indicated the faster cumulative percentage changes, which varied relatively gentle for short k-mers (10- to 17-mers) and gradually increased steep-slope for longer k-mers (18- to 64-mers) (Fig. 2a-c). For the same repetitivity frequency, a shorter k-mer motif has a higher cumulative percentage (and higher frequency in raw reads; Fig. 3a-c). Among the four *Avena* species, 16-mer motifs occurring ≥10 times accounted for 44% of *A. sativa* genome, somewhat higher than other species (28% of *A. brevis*, 34% of *A. hirtula*, and 40% of *A. strigosa*; Fig. 3d). The 64-mer motifs occurring ≥10 times accounted for 11% of *A. strigosa* genome (Fig. 3e), higher than other species (5% of *A. sativa*, 2% of *A. brevis*, and 4% of *A. hirtula*) genomes. Overall, the graphs were consistent with the RepeatExplorer analysis, with a group of very abundant sequences representing about a quarter of the genome (inflection in e.g. the 16-mer graph, Fig. 3d), and other abundant sequences representing about 70%, before a flatter region of the graph with motifs represented less than 10 times per genome. For 16-mer motifs occurring ≥10 times, the cumulative percentage of common oat was nearly equivalent to *Petunia axillaris*, followed by *A. strigosa*, sorghum, *A. hirtula*, *A. brevis*, tomato, and potato (Fig. 3f [21, 39–41]); it was notable that the cumulative percentage of 16-mer motifs occurring ≥1000 times converged for *Avena* species, tomato, and potato.

### Chromosomal location and genome specificity of highly repetitive motifs

#### Repetitive fragments used as FISH probes

**Fig. 4:**
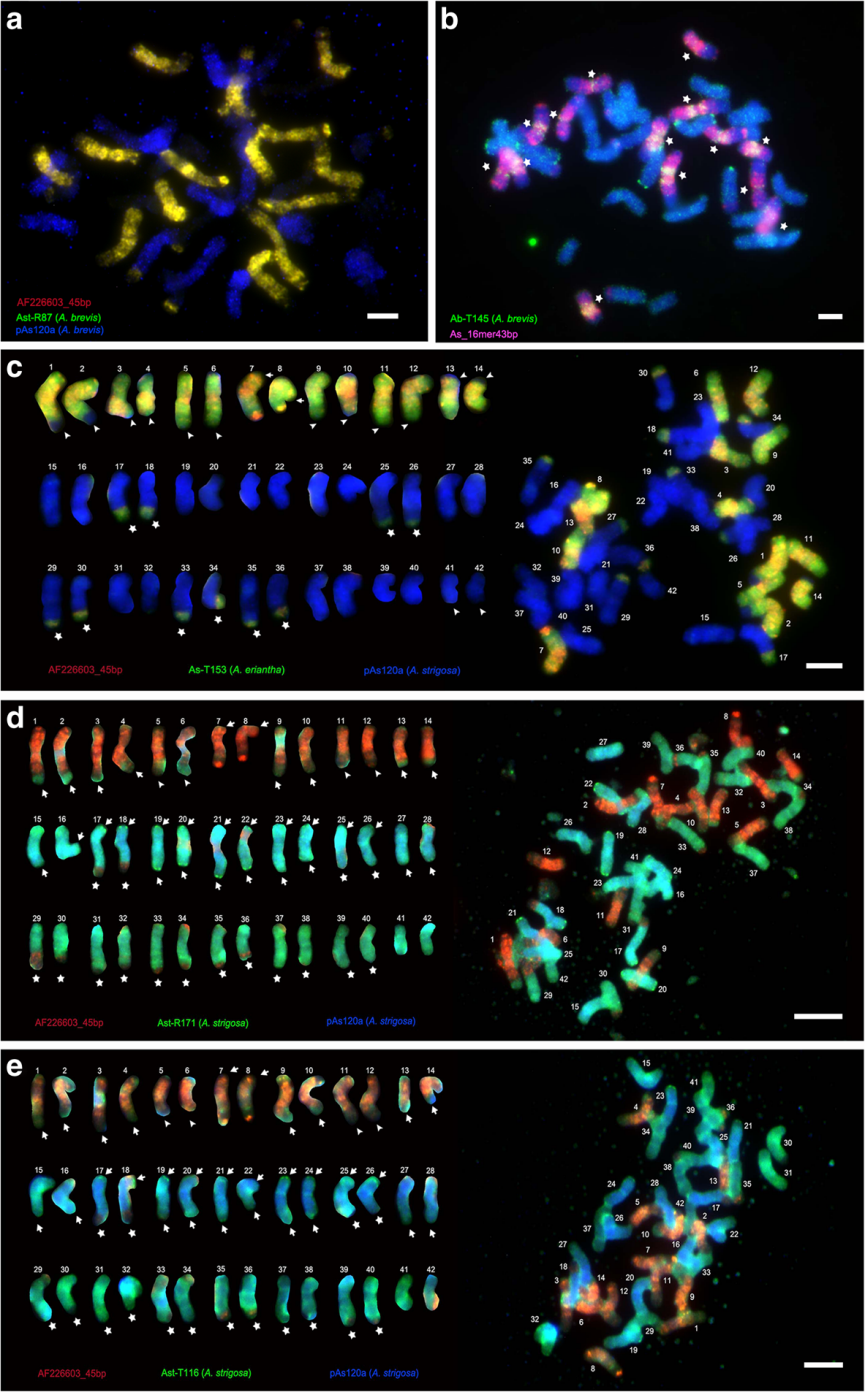
Localization of repetitive sequences on *A. sativa* metaphase chromosomes by in situ hybridization. Root tip chromosomes fluoresce cyan with DAPI (UV excitation) stain; probe hybridization sites are detected by fluorescence shown in red, green and/or blue (false colour from near-infra-red fluorescence). (A-C) C-genome specific probes; (D-E) D-genome specific probes. **a** Probes AF226603_45bp (red), Ast-R87 (green; yellow where probes overlapping) and pAs120a (blue) from *A. brevis*. **b** Probes As_16mer43bp (red) and Ab-T145 (green). (**c**-**e**) Metaphases (right) and chromosomes arranged as karyotypes showing genome origin (left) with chromosomes approximately paired by probe pattern and size (Chromosomes 1–14 C-genome; 15–28 A-genome; 29–42 D-genome). White asterisks, arrows, and arrowheads indicate notable C-, A-, and D-chromosome signals. **c** Probes AF226603_45bp (red), As-T153 (amplified from *A. eriantha*; green) and pAs120a (blue). **d** Probes AF226603_45bp (red), Ast-R171 (green), and pAs120a (blue) both amplified from *A. strigosa*. **e** Probes AF226603_45bp (red), Ast-T116 (green), and pAs120a (blue). Scale bars = 5 μm

**Fig. 5:**
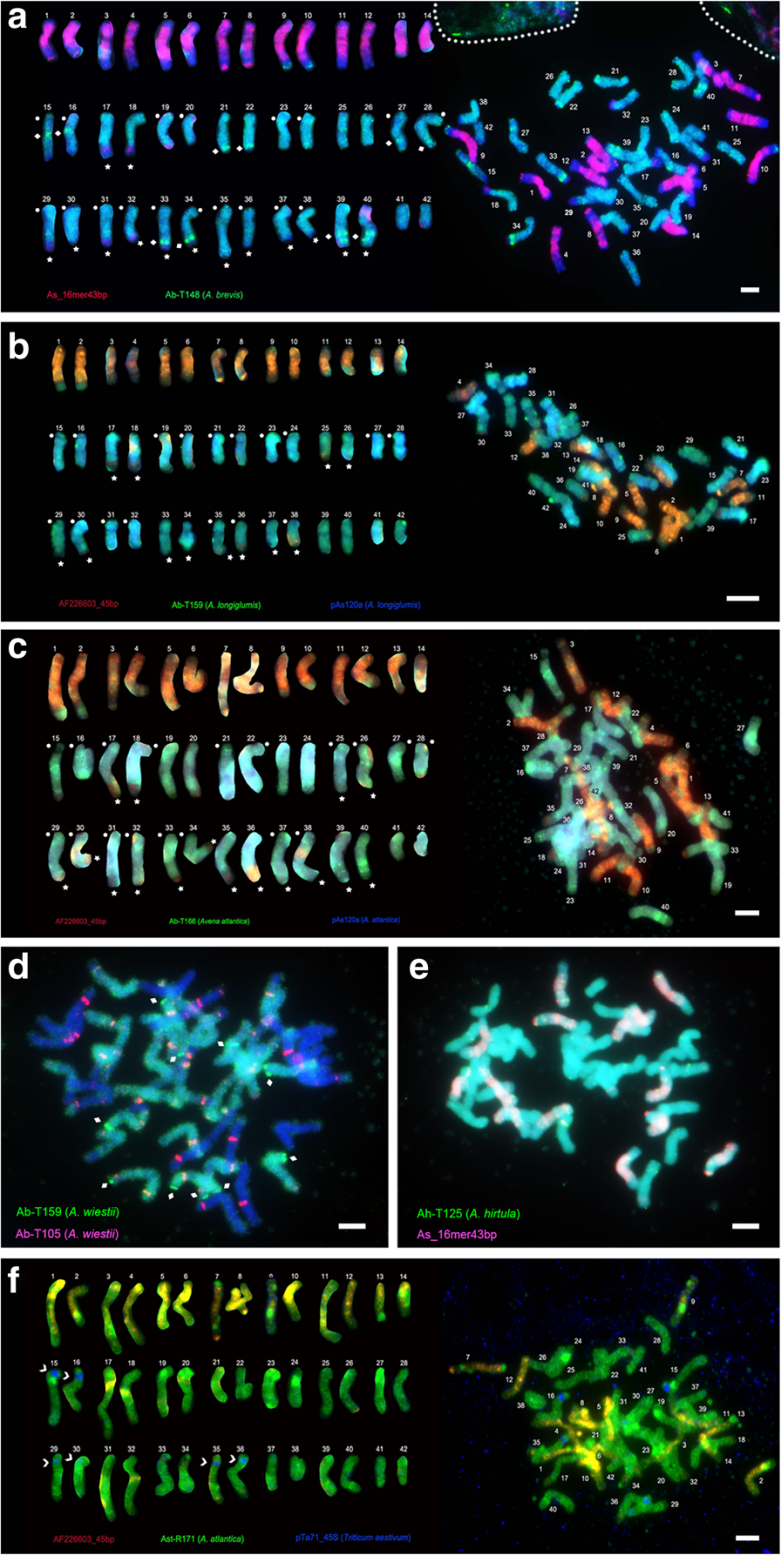
Localization of repetitive sequences on *A. sativa* metaphase chromosomes. Details of colours and arrangements as Fig. 4. **a** Probe As_16mer43bp (red) and A + D-genome specific probe Ab-T148 (green). **b** Probes AF226603_45bp (red), pAs120a (blue) and A + D-genome specific probe Ab-T159 (green). **c** AF226603_45bp (red), A/D-genome specific Ab-T166 (green), and pAs120a from *A. atlantica*. (blue). (F) Probes AF226603_45bp (red), Ab-T148 (green) from *A. atlantica* and 45S rDNA probe pTa71 (blue, denoted by white angle brackets). **d** Probes Ab-T159 (green) and Ab-T105 from *A. wiestii*. (red). **e** Probes Ah-T125 (green) from *A. hirtula* and As_16mer43bp (red). Scale bars = 5 μm

To localise repeats on *Avena sativa* chromosomes (Figs. 4 and 5, Additional files 3-10: Figures S3-S10), 25 probes were designed from representative sequences identified by k-mer and RepeatExplorer analyses to use for in situ hybridization, including nine satellite, one DNA transposon, four LTR-Gypsy retroelements and eleven unclassified sequences (Additional file 19: Table S7). Except for AF226603_45bp and pAs120a [36, 37], we selected sequences with little or no homology to repetitive elements (TEs or tandem repeats) previously isolated by PCR or cloning strategies [30, 42]. 45S and 5S rDNA [34, 35] were used to identify some chromosomes (Additional file 18: Table S6).

**Fig. 6:**
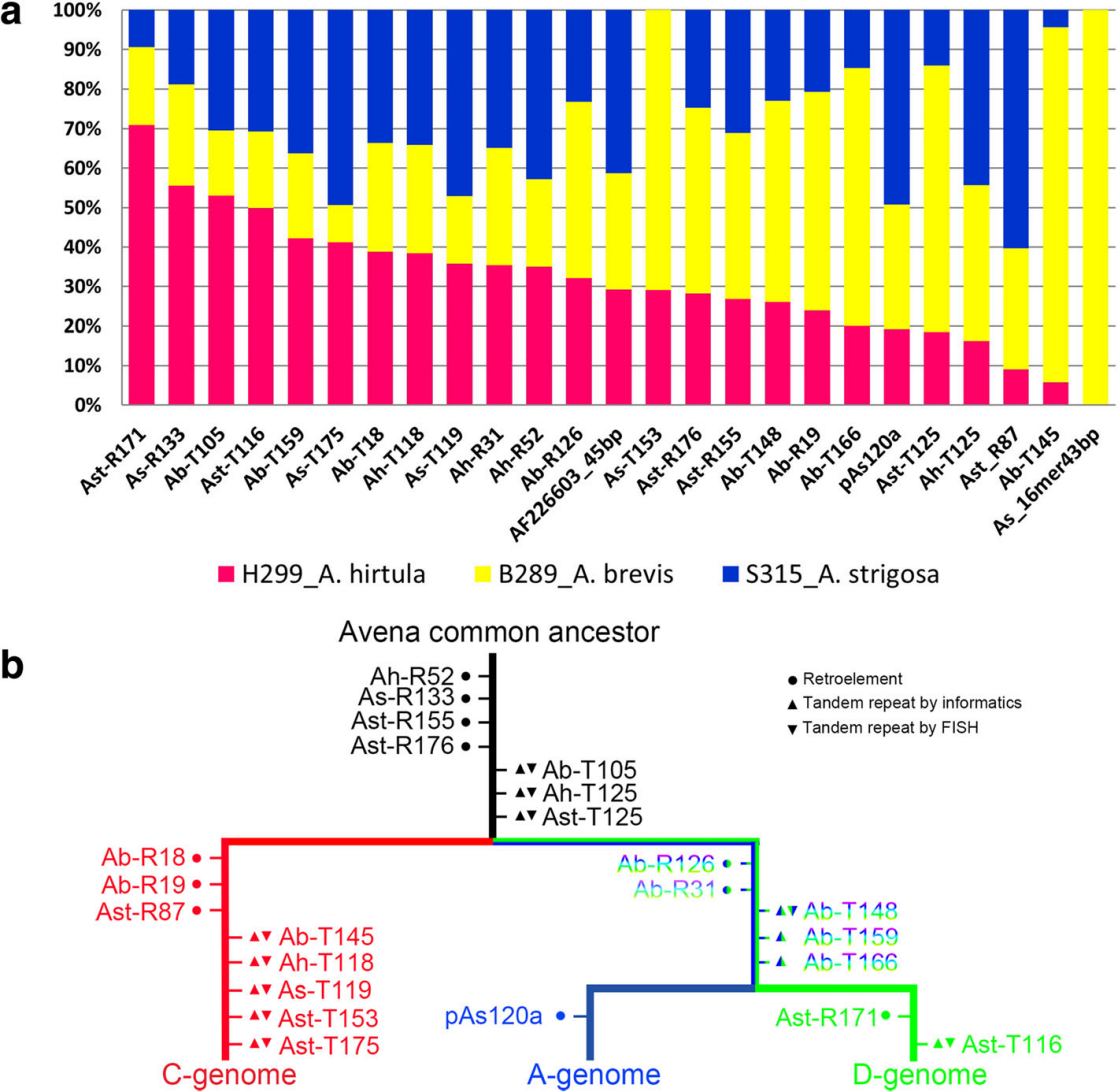
Relative proportions and an evolutionary model of repetitive DNA motifs in common oat genomes. **a** Relative proportion of repeats in *A*. *hirtula*, *A. brevis*, and *A. strigosa* genomes denoted by pink, yellow, and blue columns, respectively. **b** Evolutionary model of repetitive DNA motifs. Red, blue, and light green characters denote C-, A-, and D-genome specific repeats, respectively; blue-green gradient characters denote A + D-genome specific repeats, and black characters denote FISH probes labelling C-, A- and D-genomes. Ab, *A*. *brevis*; Ah, *A. hirtula*; Ast, *A. strigosa*; As, *A. sativa*. T, tandem; R, retrotransposon. ●: retroelement; ▲: tandem repeat verified by bioinformatics; ▼: tandem repeats verified by in situ hybridization; NCBI blast results of tandem repeat families see Additional files 22: Table S10

Copy numbers and relative proportion of the selected probes were analysed in silico in *A. sativa* and three A-genome diploids (Additional file 20: Table S8a) to check abundance and genome specificity. Most repeats (92%) were present in all four genomes (Additional file 20: Table S8a), with expected variation from over 3 million copies per genome (As-16mer43bp in *A*. *sativa*) to being almost undetectable. Eight of the fragments used for in situ hybridization had 335-360 bp monomers (Additional file 18: Tables S6, Additional file 19: Table S7, Additional file 20: Table S8a). The monomer number shown in dotplots (Fig. 2f, j, m and o) was a consequence of variability between monomers and clustering algorithm, and not related to genome structures: tandem repeat counts require very long reads (e.g. Nanopore or PacBio Sequel) or chromosome walking (e.g. BAC clones). Repeat copy numbers in the three diploid A genome species analysed was not the same (Fig. 6a), showing the whole spectrum of distribution and indicating differential amplification or loss after evolutionary separation.

No repeat was predominant in *A. strigosa*, a species where repeats As-T153 and As-16mer43bp were also absent. One repeat family (Ast-R171) was much more abundant in *A. hirtula*, and four families (As-T153, Ab-T166, Ast-T125 and Ab-T145) were dominant in the *A. brevis* genome (Fig. 6a, Additional file 20: Table S8a). The As-16mer43bp repeat was only abundant in *A. brevis*, being absent in *A. strigosa* and only 260 copies in *A. hirtula*.

For in situ hybridization, sequence fragments were synthesized as end-labelled oligonucleotides, or amplified by PCR from genomic DNA of *Avena* (A-genome species *A. brevis*, *A. hirtula*, *A. strigosa*, *A. atlantica*, *A. wiestii*, and *A. longiglumis* and C-genome species *A. eriantha*; Additional file 18: Table S6). Results from in situ hybridization of 25 repetitive sequences identified here to *A. sativa* metaphase chromosomes using two or three probes simultaneously are summarized in Additional file 21: Table S9, Figs. 4 and 5, Additional files 3-10: Figures S3-S10). As predicted from k-mer and RepeatExplorer analyses (Additional file 20: Table S8a), all probes gave hybridization signals and signal strength was generally in accordance with in silico copy number estimated.

*Avena sativa* chromosomes were assigned to genomes based on karyotype [43] and probe knowledge (particularly the A-genome specific probe pAs120a [35], C-genome specific AF226603_45bp [37], 45S and 5S rDNA). Chromosomes were numbered by descending order of sizes and arranged in pairs using morphology and hybridization patterns: chromosomes 1–14, 15–28 and 29–42 belong to C-, A-, D-genomes, respectively (Figs. 4c-e and 5a-c). Both in situ hybridization patterns and bioinformatic copy number counting allowed us to classify repeat sequences into five categories depending on genome specificity: C-, A-, and D-genome specific repeats showed stronger hybridization to chromosomes of one genome (Fig. 4a-e, Additional files 4, 5 and 6: Figures S4-S6); additional repeats were A + D-genome specific or present with similar strength in all three genomes (Fig. 5a-e, Additional file 7, 8 and 9: Figures S7-S9).

#### C-genome specific repeats

Ten non-homologous in situ hybridization signals were detected at intercalary, pericentromeric, and subtelomeric regions on 14 C-genome-origin chromosomes. Four unlabelled terminal regions we seen on pairs 5/6 & 11/12 indicative of C-D and C-A translocations, respectively (Fig. 4d-e, Additional file Additional files 4, 5 and 6: Figures S4-S6).

Repeat As_16mer43bp is highly abundant with over 3 million copies, representing 1.45% of the *Avena sativa* genome (Fig. 2a, Additional file 20: Table S8a; a synthetic labelled oligonucleotide was used as probe), and showed strong signals, being dispersed along all C-genome chromosome arms with stronger signals at most pericentromeric regions, and weak dispersed signals on distal regions of 14 A or D-chromosome long arms (17/18 & 29–40; Fig. 5a). The less abundant repeat AF226603_45bp (0.33% of *A. sativa* genome; Additional file 20: Table S8a) showed a similar distribution pattern: abundant on 14 C-chromosomes (755,507 copies; Fig. 2b, Additional file 20: Table S8a), with dispersed signals along arms and pericentromeric regions except for ends of 12 C-chromosomes 1–6 & 9–14 (Fig. 4c-e). AF226603_45bp signals were also present at the long arm terminals of 10 A + D-chromosomes with distinctive signals on nearly half of pair 17/18 long arm and one third in chromosomes 25/26, 29–40 (Fig. 4c-e, Additional file 4: Figure S4a-4f, Additional file 5: Figure S5a-S5f Additional file 6: Figure S6a-S6 f). Additionally, nucleolus organizer regions (NORs) were observed using probes Ab-T148, Ab-T159, and Ast-T116 on 20 A + D-chromosomes 15/16, 19–24 & 27–38 (Figs. 4e and 5a, b, Additional file 5: Figure S5a-S5f, Additional file 9: Figure S9a-S9f).

Several retrotransposon repeats, Ab-R18, Ab-R19 and Ast-R87 (Fig. 2c, d and e), but also tandem repeats Ab-T145, As-T153 and Ah-T118 (Fig. 2f, g and i) showed dispersed signals, with high abundance on 14 C-chromosomes (Fig. 4b, Additional file 3: Figure S3a-S3b) and much less or no signals on A- and D-chromosomes (Fig. 4a and c).

Other probes labelled only some C-genome chromosomes and showed additionally more uniform signals on all chromosomes indicating large tandem arrays of at least 20 kb to see FISH signals as double- or more-dots (Additional file 3: Figure S3d and S3e), e.g. As-T175 and As-T119 (Fig. 2h and j).

#### A-genome specific repeats

Retrotransposon-related probes (RepeatExplorer Clusters 289CL22, 299CL11, 312CL17 and 315CL22 harboured homologous sequences to the published pAs120a [36]; Fig. 2k). In situ hybridization with probes from all six diploids showed similar and uniform signals on all A-chromosomes with several ends missing, but additional signals at ends of some other chromosomes indicating A-C (17/18 & 25/26; Fig. 4c) and A-D (15/16, 19–24 & 27/28; Fig. 4d, e, Additional file 4: Figure S4a-S4f, Additional file 5: Figure S5b-S5c and S5e-S5f) translocations.

#### D-genome specific repeats

Retrotransposon Ast-R171 (Fig. 2l) and tandem repeat Ast-T116 (Fig. 2m) showed strong signals predominantly on D-chromosomes (Fig. 4d, e, Additional files 4-5: Figures. S4a-S4f and S5a-S5f), with several bands on A-chromosomes and minimal signals on C-chromosomes (Additional file 4: Figure S4b-S4c and S4e-S4f, Additional file 5: Figure S5b-S5c and S5e-S5f). Ast-R171 showed D-C translocations with subterminal double-dot signals on 12 D-chromosomes (29–40; Additional file 4: Figure S4a-S4f), but missing terminal signals on 12 D-chromosomes (29–40) that in turn show C genomic repeats (Fig. 4d, Additional file 4: Figure S4a-S4f). Similarly, Ast-T116 was missing from 12 D-chromosome long arm terminals (chromosomes 29–40; Additional file 5: Figure S5a-S5f) that either showed A- or C-genome-specificity. Ast-T116 showed typical signals of tandem repeats, i.e., double-dots on one or both terminals of A- chromosomes 17–26 (Fig. 4e, Additional file 5: Figure S5b and S5e-S5f). Sometimes, weak translocation signals were observed on long arm terminals of chromosomes 31/32 & 37–40 in Fig. 4d and Additional file 6: Figure S6a-S6c, and 29–34 & 37/38 in Fig. 4e and Additional file 6: Figure S6d-S6 f.

#### A + D-genome specific repeats and repeats labelling C-, A- and D- genomes

Signals of five in situ hybridization probes were almost uniform on A + D-chromosomes but relatively weak on C-chromosomes (Fig. 5a-d, Additional files 7, 8 and 9: Figures S7-S9). Probes for a retrotransposon Ab-R126, Ah-R31 (Fig. 2n), and tandem repeat Ab-T148 produced dispersed signals on A + D-chromosomes with 16 unlabelled A + D-chromosome terminals (17/18, 25/26 & 29–40; Additional files 7, 8 and 9: Figures S7-S9), and some unevenly intercalary bands across A + D-chromosome arms (Fig. 5a, Additional file 7: Figure S7e, Additional file 8: Figure S8e, Additional file 9: Figure S9c-S9e). Tandem repeat Ab-T159 (Fig. 2o) and Ab-T166 showed evenly dispersed and weak pericentromeric band signals on A + D-chromosomes; intergenomic translocations were observed on 12 A + D-chromosome terminals (17/18, 25/26, 29–32 & 35–38) and on 12 C-chromosome terminals (1–6 & 9–14), with dispersed signals on A + D-chromosomes like retrotransposons (Fig. 5b-d, Additional file 6: Figure S6i).

Seven probes showed dispersed signals along C-, A- and D-chromosomes (Additional file 21: Table S9). Retrotransposons Ah-R52 and repeats As-R133, Ah-T125, Ast-R155 and Ast-R176 showed dispersed signals on C-, A- and D-chromosomes (Figs. 2p and 5d-e, Additional file 10: Figure S10a-S10f). Sequence Ab-T105 and Ast-T125 showed intercalary double-dots on C-, A- and D-chromosomes (Fig. 5d, Additional file 10: Figure S10f).

#### rDNA sites

45S and 5S rDNA sequences occupied about 0.5% of the *Avena* genome (Additional file 17: Table S5). Tandem repeat 45S rDNA (9059 bp) contained 3197 bp, 1811 bp, 260 bp, 168 bp, 216 bp and 3407 bp of ETS, 18S, ITS1, 5.8S, ITS2 and 26S, respectively, while tandem repeat 5S rDNAs (544 bp) contained 180 bp coding sequences and 158-206 bp intergenic spacers (Additional file 11: Figure S11a-S11b). Six 45S rDNA loci were identified on six NORs of A and D-chromosomes (15/16, 29/30 & 35/36), and fourteen 5S rDNA loci were identified on six NORs plus eight intercalary positions of A and D-chromosomes (15/16, 29/30, 31/32 & 35/36), respectively (Fig. 5f, Additional file 11: Figure S11c-S11e). The 5S rDNA signals collocalized with 45S signals on six NORs of A- and D-chromosomes (Fig. 5f, Additional file 11: Figure S11e), plus extra pairs of 5S intercalary signals located on pair 31/32 (Additional file 11: Figure S11d-S11e). Extended chromatin and hybridization signals were visible on six NOR A- and D-chromosomes (Additional file 11: Figure S11e) indicating the NOR-rDNA transcription was more active on A- and D- compared to C-chromosomes.

## Discussion

### Identification and abundance of repetitive DNAs

#### Genome wide

Analysis of unprocessed *Avena* genomic DNA sequence reads using motif counting (k-mer analysis) and graph-based clustering shows that repetitive DNA sequences represent some 72% of the genome (Fig. 1, Table 1). Combining the in silico analysis with molecular cytogenetics on chromosomes in situ, we could identify the nature of the motifs and measure their abundance to give a comprehensive survey and evolutionary relationships of the repeat landscape of oat (Figs. 1 and 6). Notably, 96% of the sequences examined here could be classified as being related to either transposable elements or a relatively small number of tandemly repeated motifs (Figs. 1 and 2). Our strategy would not expect to reveal microsatellite motifs, short runs of dinucleotide or trinucleotide repeats with unique flanking regions, known to have an uneven distribution across the genome [44]. While there are increasing reports of genome-wide repeat surveys [13, 45, 46], most sequence assemblies collapse repeats to variable extents [21, 47], while library screening or PCR amplification with primers are selective. Thus detailed comparisons between our results and many published analyses using whole genome assemblies, reference repeats (e.g. RepeatMasker), or targeted screening may not be valid. Furthermore, classification of “families” within major groups of repeats is flexible, with some distinct families, and others where there are intermediates between sequences that would otherwise be regarded as distinct. Many of the major families of repeats identified here have been identified previously in selective screens of DNA libraries [30, 36], although these studies could not quantify their abundance in the various diploid and the hexaploid genomes. Importantly, unlike the analysis of unprocessed random reads here, selective screens cannot show that all the repetitive components of the genome have been surveyed.

The 16-mers identified by our k-mer analysis with more than 10 copies per genome correspond to the figures from potato and tomato (see Fig. 3f). The 16-mers occurring less than 10 times represented between 24 and 40% of the oat genome (Fig. 3), indicate a relatively high variation within repeat sequence motifs, and these families may not be detected by reassociation kinetics (experimental) or graph-clustering (bioinformatics). Overall, the proportion of 16-mers occurring 10 or less times in *Avena* is similar to the 30% in *Petunia* or *Sorghum* (Fig. 3f). However, the variation between four *Avena* species (24, 28, 34 and 40%; Fig. 3) is hard to explain but may suggest greater homogenization in *A. strigosa*. A change in slope, as seen in *A. strigosa* for k-mers longer than 16 bp (Fig. 3), could be related to the frequencies of different repetitive DNA classes or their homogenization, but there were no conspicuous differences in repeat classes between *A. strigosa* and the other diploids (Additional file 14: Table S2). *A. sativa* shows a weaker change in graph slope (Fig. 3), consistent with addition of the genomes from one *A. strigosa*-like and two other species. LTR retrotransposons are largely responsible for the dramatic differences in genome sizes between related plant species, e.g. six-fold size difference between maize and rice genomes [48, 49] so they could equally play a role here.

#### Transposable elements

Major families of LTR retrotransposons represented 60% of the genome and 85% of the repeatome in *Avena* (Fig. 2), a value that varies widely in different species. In *Brachypodium distachyon* (genome size 272 Mb) retrotransposons represent 21.6% of the genome or 26% in rice (430 Mb [50]), or 85% in bread wheat (6*x*, 16,000 Mb [51]) and ‘over 80%’ reported in barley (2*x* = 14, 5100 Mb [46]). In *Avena*, Ty3-Gypsy like LTR-retrotransposons (retroelements) were 2.3 to 2.7 times more abundant than Ty1-Copia like sequences (Figs. 1 and 2, Additional file 17: Table S5). These values are closely similar to the three wheat genomes (ratio 2.5 to 2.9 [51]), although differs in other species: total repetitive DNA occupies 73% of the *Coix aquatica* genome (1.7 Mb), but the Gypsy/Copia is only 0.74 [52]. DNA transposons, shorter in element length than retroelements, represented 5.5% of the *Avena* genome, with CMC-EnSpm like sequences most abundant. This relatively high value is similar to *Brassica oleracea* HDEM (4.69% [53]) and *Petunia* species (4.64–5.21% [21]), although other species are much lower including another *Brassica*, *B. rapa* Z1 (1.72% [53]), and the Solanaceous *Solanum* and *Nicotiana* species (0.6–1.51% [21]).

All genomes have mechanisms controlling TE amplification. Schorn et al. [54] have shown in *Arabidopsis* how RNA-driven DNA methylation is responsible for silencing, as is most likely the case for pararetrovirus sequences [55]. Large genomes bear higher proportions of TE sequence, and Lyu et al. [56] suggested that TE load reduction is the most important driver of genome diminution in mangroves. Here, it is notable that oat retrotransposon-related repetitive sequences families vary in abundance between diploid species, and some are essentially specific to one or two of the genomes (Fig. 6), suggesting loss and gain of particular families and directed turnover.

#### Tandem/satellite repeats

Tandem repeats or satellite DNA is a feature of most eukaryotic genomes. Here, we found 12 tandem repeat families, in eight of which the monomer lengths were 335 bp–360 bp (Additional file 22: Table S10). This has been noted as a monomer length required to wrap around two nucleosomes (~ 150 bp DNA for a single nucleosome) spaced by a variable unwrapping linker region of ~ 30–60 bp [16, 57]. Structural interactions between nucleosomes and DNA repeats can impact chromatin dynamics [58, 59] and the stable wrapping of tandem repeats could be important for genome stability and methylation of domains leading to silencing. Tandem repeat probes show discrete signals on common oat chromosome arms (Additional file 3: Figure S3d-3e, Additional file 5: Figure S5e-S5f) indicating large arrays of at least 20 kb, but some also show dispersed signals at intercalary sites (Fig. 5b-d), likely to represent multiple smaller arrays.

Submotifs of a repeat family can be used as genome-specific probes for in situ hybridization, e.g. the *Brassica* C-genome specific CACTA transposon [60]. Here, probe As_16mer43bp motif was found 3,346,757 reads in *A. sativa*, but was absent in *A. strigosa* (Additional file 20: Table S8a). Another repeat AF226603_45bp motif was first identified by Southern blot analysis [61] as being abundant. In contrast, two short 45 bp motifs of unknown family produced uniformly dispersed signals on common oat C-chromosomes (Fig. 4a, c-e). They are a relatively unusual length for plant repeat motifs, although c. 60 bp-length minisatellites are common in mammals [62].

rDNA was used as chromosome-specific probes. Based on phylogenetic evidence, in *Avena*, two NORs (45S rDNA sites) per haploid chromosome set were ancestral characters, while chromosome complements with 4 or more NORs were derived characters [63]. Structurally, the elimination of C-genome rDNAs and partial elimination of A-genome rDNAs following a hexaploidization event in *A. sativa* indicates that rDNA from one ancestor (the paternal genome (see [31]) might be silenced and lost following hexaploidization in *A. sativa*. Similar rapid loss of 45S rDNA sites is also seen in the tetraploid wheat-relative *Aegilops ventricosa* (with DDNN genome designation), where D-genome 45S rDNA sites are lost [64].

### Evolution of repeats

#### Diploid speciation and repetitive DNAs

The karyotypes with repetitive sequence locations provide a fresh perspective in understanding evolution in *Avena*. A-genome specific pAs120a was isolated long ago [36]. They discussed the repeat length and existence of four monomers inserted within pAs120a, suggesting cautiously that the pAs120a sequence could be classified as a satellite DNA sequence. In contrast, our sequence clusters homologous to pAs120a showed high similarity to Ogre/Tat and chromovirus retrotransposons (Fig. 2k) indicating that this repeat originated from a retroelement, and might have been generated as a tandem repeat from a retroelement subregion by rolling circle amplification. However, twenty years later, we still share the uncertainty of Linares et al. [36] about the evolution of this sequence to become A-genome-specific. Other sequence families also show differential amplification or reduction in individual *Avena* A-genomes (Fig. 6a), and in the hexaploid compared to the diploid ancestral genomes; e.g. the higher abundance of C-chromosome specific motifs identified in *A. sativa* genome (e.g. As-T153) or high abundance of D-chromosome specific motifs identified in *A. hirtula* (Ast-T116; Fig. 6a, Additional file 20: Table S8a).

From molecular dating analyses, the crown age of the C-genome diploid lineage was ~ 20 Mya, older than the crown age of A-genome polyploid lineages [31, 32]. This is supported by greater proportion of C-genome specific motifs, diverging from the common ancestors before the radiation of A- and D-genome specific motifs, as the A- and D-genome specific motifs amplified independently in common oat (Fig. 6b). This evolutionary scenario is also supported by repeats common to the A- and D-genomes or all three genomes, but no repeats were found to be specific for the C- and A- or C- and D- genomes. Retrotransposons may have a role in genome behaviour by acting as nuclei for RNA-dependent DNA methylation (as [65]), leading to position effect variegation via heterochromatinization around repetitive elements affecting adjacent gene expression [66, 67].

### Distal chromosome regions and translocations

The repetitive sequences used as probes here show major genomic changes through chromosome translocations in common oat [43]. In situ hybridization is unique as a method to show the nature and extend of these translocations, and the use of the repetitive probes (Figs. 2 and 3) isolated here show three kinds of intergenomic translocations:

(a) **C-genome segments translocated onto A- and D-chromosomes** — four A-C translocations (pairs 17/18 & 25/26) identified by the AF226603_45bp probe (Fig. 4c, Additional file 9: Figure S9a and S9d-S9e), and 12 D-C translocations (chromosomes 29–40) identified by AF226603_45bp probe (Additional file 3: Figure S3a, S3c and S3f, Additional file 5: Figure S5a-S5f);

(b) **A- or D-genome segments translocated onto C-chromosomes** — four C-A translocations (pairs 5/6 & 11/12) identified by pAs120a probe (Additional file 4: Figure S4c-S4d, Additional file 5: Figure S5c-S5d); and 10 C-D translocations (chromosomes 1–4, 7–10 & 13/14) identified by Ast-T116 probe (Fig. 4d-e, Additional file 5: Figure S5a-S5c and S5f);

(c) **Between A- and D- genome translocations** — 10 A-D translocations (chromosomes 15/16, 19–24 & 27/28) identified by Ast-R171 and Ast-T116 probes (Additional file 4: Figure S4b-S4c and S4e-S4f, Additional file 5: Figure S5b-S5c and S5e-S5f), and two D-A translocations (pair 41/42) identified by pAs120a probe (Additional file 5: Figure S5b and S5f, Additional file 7: Figure S7b and S7f).

Translocations can alter chromosome recombination frequencies, and can lead to genetic and evolutionary isolation of new hybrids. The result also suggests the opportunity for introduction of genetic variation as small chromosome segments from wild diploid *Avena* species into the hexaploid, with recombinant segments involving any genome, or potentially more distant diploid relatives. The oat intergenomic translocations contrast with wheat, where there are no reports of stable intra-genomic translocations with the possible exception of the 4A and 4B linkage groups, with the 4A chromosome differing substantially in repeat content from other chromosomes in the A and B genomes. In oats, the rearranged chromosomes may have adaptive value by enabling different expression levels and modulation of expression of genes from the homoeologous genomes.

### Large scale genome organization and implications

Genomic repeats provide the physical basis for integrating different genomic regions for coordinating interdependent aspects of genome functions [68]. Common oat was inferred to have originated following ancient allotetraploidy (D- and C-genomes) and recent allohexaploidy (ACD-genome) events in the subfamily Pooideae [31]. Given the repeat abundance spanning A- and D-chromosomes, we speculate that the diverged repeats of *A. strigosa* (D-genome) and *A. atlantica*-*A. brevis*-*A. longiglumis*-*A. wiestii* (A-genome) might represent the basis of evolutionary separation of A- and D-genome progenitors, as in other species [69]). Genome-specific repeat amplification, followed by subgenome-function divergence, has been suggested to provide a mechanism driving cold acclimatization in *Avena* [70], and polyploidization with subgenome dominance may support rust resistance phenotypes that ultimately correspond to agronomic traits [26]. Many evolutionary models suggest that polyploid formation should be associated with a selective advantage, favouring parental genome divergence. Given that six of 13 retrotransposons plus three tandem repeats were more abundant in common oat than in diploid relatives (Additional file 20: Table S8a), it is reasonable to speculate that a burst of ancient repeat-associated genomic duplication may explain expansion of the oat genome size.

## Conclusion

Here, the complete repetitive DNA content of *Avena* has been surveyed in whole genome data, and the repeats make up 70% of all the DNA. The most abundant elements were previously described, and we show that a small number of repeat families, some not described before, contribute a high proportion of all the repetitive DNA. It is clear that repeat amplification and turnover of repeat families have been involved during evolutionary separation of the ancestors of common oat, and, in combination with frequent intergenomic translocations (not seen in other cereals) and further turnover events, have led to the rapid evolution seen in the hexaploid. Transposable elements are a major contributor to the genome, although the families present, and their relative abundances, differ in *Avena* from other species groups. Used as a source of DNA markers or chromosomal probes, retroelements have utility in crop breeding [71] and tracking chromosomes in hybrids and translocation lines. With increasing data coming from long-read technologies (including Nanopore, PacBio Sequel and high-throughput chromosome conformation capture), knowledge of the repeat landscape is useful in optimizing the approach to genome sequence assembly by accounting for the abundance and genome distribution of only a small number of repeat families.

## Methods

### Plant material

Eight *Avena* species (origin of samples is given in Additional file 13: Table S1, chromosome and genome designation see Liu et al. [31], seeds were obtained from CN-Saskatchewan and USDA-Beltsville Germplasm System) were used in this study. The 171.9 Gb of raw sequences representing 4.58× to 7.06× coverage of *Avena* genomes [*A. sativa* (66.1 Gb), *A. brevis* (34.6 Gb), *A. hirtula* (35.3 Gb), and *A. strigosa* (35.9 Gb)] were generated by whole-genome shotgun sequencing with 2 × 250 bp from 500 bp paired-end libraries (Nanjing Genepioneer Biotechnologies Co. Ltd., Illumina HiSeq2500 platform; Additional file 1: Figure S1, Additional file 14: Table S2a). Project data have been deposited at the National Center for Biotechnology Information (NCBI) under BioProject PRJNA407595 (SRR6056489–6056492).

### Repeat discovery

#### Graph-based clustering of sequences

Similarity-based clustering, repeat identification, and classification of a subset paired-end raw reads (1.72–2.87 GB occupied 2.60–8.29% of *Avena* genomes; Additional file 14: Table S2b) were performed by RepeatExplorer analysis (Additional file 15: Table S3). It was set with read overlaps containing ≥50% of length with 90% of similarity as edges to save the potential error of “bridge” reads with partial similarity among two unrelated communities [15]. The longest contigs in each of 821 clusters were analysed by BLAST search against NCBI database to check for repeat identification (Fig. 1, Additional file 16: Table S4) and repetitive DNA composition was summarized manually (Additional file 17: Table S5). Primer pairs were designed from one contig of each retroelement or tandem repeat belonging to clusters of no (or less) 1st-order neighbours [72] (Additional file 12: Figure S12a-S12 t, Additional file 18: Table S6, Additional file 19: Table S7), probe designations use genus and species abbreviation plus T for tandem or R for retrotransposon type followed by the cluster number. Primers designed from clones pTa71 [34] and pTa794 [35] were used for 45S and 5S rDNA amplification respectively (Additional file 18: Table S6). Cluster graphs, dotplots and FISH probe copy numbers were investigated by SeqGrapheR v.3.3.1 [15] and Geneious [38] (Fig. 2a-p, Additional file 20: Table S8).

#### k-mer analysis

Genome repetitivity was assessed via 10- to 64-mer frequencies by the program Jellyfish v.2.2.6 [14] for four *Avena* species (Fig. 3a-e). Frequencies were compared with *Petunia axillaris*, potato, sorghum, and tomato [21, 39–41] (Fig. 3f). The As_16mer43bp consensus sequence was used to design a synthetic oligonucleotide probe (Additional file 18: Table S6), its position was aligned to RepeatExplorer cluster graph layouts (Fig. 2a).

### Multicolour fluorescence in situ hybridization (FISH)

Root tips were fixed in 96% ethanol: glacial acetic acid (3:1) for at least 1.5 h and stored in the fixative at − 20 °C overnight. An enzyme solution with 0.2% Cellulase Onozuka R10 (Yakult Pharmaceutical, Tokyo), 2% Cellulase (C1184 Sigma-Aldrich) and 3% Pectinase (P4716; Sigma-Aldrich, St Louis, USA) was used to digest root tips for 90 min at 37 °C. Root tips were macerated in a drop of 60% acetic acid, and roots were squashed gently under a coverslip.

PCR amplification was performed in 25 μl reaction mixture containing 50–100 ng genomic DNA, 0.2 μM each primer, 0.4 mM deoxynucleotide triphosphate mix, 1 × PCR buffer, 2.5 mM Mg^2+^, and 0.5 U Taq DNA polymerase (Kapa Biosystems). PCR conditions were 95 °C for 3 min, followed by 35 cycles of 95 °C for 30 s, 51.3–68.0 °C for 30 s, and 72 °C for 1 min. PCR products were electrophoresed on 1.0% agarose gels, purified using an E.Z.N.A. Cycle Pure Kit (Omega), and then labelled with either biotin-16-dUTP or digoxigenin-11-dUTP (Roche Diagnostics) using a BioPrime Array Comparative genomic hybridization (CGH) Genomic Labelling System (Invitrogen).

FISH was performed as described by Schwarzacher and Heslop-Harrison [73]. The 34 μl of hybridization mixture (stringency 76%), containing 50% formamide, 2 × SSC (Saline Sodium Citrate buffer; 0.3 M NaCl, 0.03 M sodium citrate) 10% dextran sulphate, 0.125% SDS (sodium dodecyl sulphate), 0.125 mM EDTA (ethylenediamine-tetraacetic acid); 1 μg sheared salmon sperm DNA and up to 1000 ng of labelled probes were applied on each slide. After denaturation at 72 °C for 7 min, hybridization at 37 °C overnight in a ThermoHybaid HyPro-20, slides were washed in 0.1 × SSC at 42 °C,. FISH probe hybridization sites were detected via fluorescein isothiocyanate (FITC) conjugated anti-digoxigenin (200 μg/ml; Roche Diagnostics), Streptavidin Alexa Fluor 594 (200 μg/ml; Molecular Probes) or Streptavidin Alexa Fluor 647 (1 mg/ml; Molecular Probes) in 4 × SSC, 0.1% Tween 20, and 5% BSA (Bovine Serum Albumin). Slides were counterstained with 4′, 6-diamidino-2-phenylindole (DAPI; 3 mg/ml)-antifade solution (AF1, Citifluor, London, UK; 50%). FISH images were captured by a Nikon Eclipse 80i epifluorescent microscope fitted with appropriate sets of t band-pass filters to capture Alexa 647, Alexa 594, Tetrachlorofluorescein (TET), FITC and DAPI, a DS-QiMc monochromatic camera, and NIS-Elements v.2.34 (Nikon, Tokyo, Japan). For each metaphase, four single channel images (pseudo-coloured, yellow, red, green, and blue respectively) of 1280 × 1024 pixel size were analysed by Image J v.1.51j8 (Wayne Rasband, NIH, USA) and superimposed in Photoshop CS6 v.13.0 (Adobe System, San Jose, CA, USA). Total 269 slides including 2353 metaphases of 8 species (average 8 metaphases per slide) were observed for in situ hybridization analyses. Probe fragment copy numbers were determined by counting the number of reads (per genome equivalent) mapping to the probe fragment sequence (“Map to Reference”).

## Additional files

Additional file 1: **Figure S1.** Spikelet morphology of eight sampled *Avena* species. a *A. atlantica*: the dispersion units—the upper florets are attached to the lower floret and only the lower floret show disarticulation. b *A. brevis:* spikelets show persistent florets with bidenticulate lemma tips at maturity. c *A. hirtula:* a Mediterranean wild type with lemma bristles 6–10 mm. d *A. longiglumis:* 2–3 florets/spikelet, each floret is disarticulated; lemma back is covered with dense hairs. e *A. strigosa:* 2–3 florets/spikelet and persistent florets. f *A. wiestii*: desert and steppe wild type with lemma bristles 5–8 mm. g *A. eriantha:* glumes markedly unequal in size. h *A. sativa:* spikelets 1.5–4 cm with typically spread glumes at maturity. Scale bars = 1 cm. (TIF 4210 kb)

Additional file 2: **Figure S2.** Distribution of graph-based clusters. Hierarchical agglomeration of RepeatExplorer analyses of four *Avena* species genomes are shown. a *A*. s*ativa* S312. b *A. brevis* B289. c *A. hirtula*. H299. d *A. strigosa* S135. Coloured bars denote clusters ≥0.01% of genome: x-axis denotes the cumulative read number percentage while y-axis denotes the read numbers in the clusters. Bars coloured according to the repeat types of cluster annotation (Additional files 15: Table S3). (TIF 1895 kb)

Additional file 3: **Figure S3.** Localization of selected C-genome specific repetitive sequences on *Avena sativa* metaphase chromosomes by multicolour FISH. Probe signals were captured individually with a black and white CCD camera and then pseudo-coloured to create overlaid images. For detailed description of signal distribution see Additional file 21: Table S9. a AF226603_45bp (hybridization sites displayed in red), Ab-R18 amplified from *A. atlantica* (green), and pAs120a from *A. atlantica* (blue). Note that overlapping signals of the red and green probe give yellow signals. b Ab-R19 (red) and Ab-R126 (green), counterstain DAPI (blue) shown on all chromosomes. Note that overlapping signals of the red and green probe appear white, but show several chromosome ends not labelled by either probe appearing blue or show green double dots. c AF226603_45bp (red), Ah-T118 from *A. hirtula* (green), and pAs120a from *A. hirtula* (blue). Overlapping signals of the red and green probe appear yellow and show non-uniform labelling of chromosomes. An interphase nucleus is visible at the bottom of the image. d As-T119 (green), double-dots (starred) appearing in yellow on top of the red signal of As_16mer43bp. DAPI fluorescence shown in blue.e As-T175 (green, double-dots) and As_16mer43bp (red) showing large blocks of hybridization signal on C genome chromosomes (starred). DAPI fluorescence shown in blue. f TET labeled AF226603_45bp (red), As_16mer43bp (pink), and DAPI (blue). Scale bars = 5 μm. (TIF 6648 kb)

Additional file 4: **Figure S4.** Fluorescent in situ hybridization (FISH) karyotyping of *Avena sativa*. Probes are AF226603_45bp (direct TET, red) for the C genome, pAs120a (biotin, Alexa 594, blue) for the A genome, and Ast-R171 (digoxigenin, FITC, green) for the D genome. On the right the DAPI image of chromosomes is shown in white, in the middle the same metaphase shows hybridization signal from all three probes except in (d) where only AF226603_45bp in yellow and pAs120 in blue are visible. Probes were amplified from different diploid species. a *A*. *brevis*. b *A. atlantica.* c *A. strigosa*. d *A. wiestii*. e *A. longiglumis*. f *A. hirtula*. In the karyotypes (on the left), chromosomes are arranged in rows corresponding to genome origin: 1–14 C-genome, 15–28 A-genome, and 29–42 D-genome. White stars, arrows, and arrowheads denoted C-, A-, and D-chromosome regions, translocated to a different genome: there are C translocations on 12 D-chromosomes (29–40); A translocations on four C-chromosomes (5/6 & 11/12); D translocations on 10 A-chromosomes (15/16, 19–24 & 27/28). Scale bars = 5 μm. (TIF 7816 kb)

Additional file 5: **Figure S5.** Fluorescent in situ hybridization (FISH) karyotyping of *Avena sativa*. Probes are AF226603_45bp (direct TET, red) for the C genome, pAs120a (biotin, Alexa 594; blue) for the A genome, and Ast-T116 (digoxigenin, FITC, green) for the D genome. On the right the DAPI image of chromosomes is shown in white, in the middle the same metaphase shows hybridization signal from all three probes except in (d) and (e) where only AF226603_45bp in yellow and pAs120 in blue are visible. Probes were amplified from different diploid species. a *A*. *brevis*. b *A. atlantica.* c *A. strigosa*. d *A. wiestii*. e *A. longiglumis*. f *A. hirtula*. In the karyotypes (on the left), chromosomes are arranged in rows corresponding to genome origin: 1–14 C-genome, 15–28 A-genome, and 29–42 D-genome. White circles denote nucleolus organizer regions (NORs) signals. White stars, arrows, and arrowheads denoted C-, A-, and D-chromosome regions, translocated to a different genome: there are D translocations on ten C-chromosomes (1–4, 7–10 & 13/14); there are C translocations on 10 D-chromosomes (29–40); A translocations on four C-chromosomes (5/6 & 11/12); D translocations on 10 A-chromosomes (15/16, 19–24 & 27/28). Scale bars = 5 μm. (TIF 8170 kb)

Additional file 6: **Figure S6.** Fluorescent in situ hybridization (FISH) of *Avena sativa*. Single channel images of far-red (a, d, g), pseudocoloured blue (b, e, h), and green (c, f, i) dotted lines circling C, A and D-genome chromosomes analysed by Image J v.1.51j8 for Figs. 4a (a-c), 4e (d-f) and 5c (g-i). (a-c) FISH probes AF226603_45bp (direct TET, red), pAs120a (biotin, Alexa 594; blue) and Ast-R171 (digoxigenin, FITC, green) and from *A. strigosa*: separation of the overlapped chromosomes (a) 6, (b) 21 & 25, 16, 23 & 24, and (c) 29 & 42 and 31 & 41 for Fig. 4d. (d-f) FISH probes AF226603_45bp (far red), Ast-T116 (green) and pAs120a (pseudocoloured blue) from *A. strigosa*: separation of the overlapped chromosomes (d) 3, 6, 12 & 14, (e) 18 & 27, and (f) 33 & 42 for Fig. 4e. (g-i) AF226603_45bp (far red), A/D-genome specific Ab-T166 (green), and pAs120a (pseudocoloured blue) from *A. atlantica*: separation of the overlapped chromosomes (g) 7, 8 & 14, (h) 18, 23, 24, 25 & 26, and (i) 31 & 36 for Fig. 5c. (TIF 6215 kb)

Additional file 7: **Figure S7.** Fluorescent in situ hybridization (FISH) karyotyping of *Avena sativa*. Probes are AF226603_45bp (direct TET, red) for the C genome, pAs120a (biotin, Alexa 594; blue) for the A genome, and Ab-R126 (digoxigenin, FITC, green). On the right the DAPI image of chromosomes is shown in white, in the middle the same metaphase shows hybridization signal. Probes were amplified from different diploid species. a *A*. *brevis*. b *A. atlantica.* c *A. strigosa*. d *A. wiestii*. e *A. longiglumis*. f *A. hirtula*. In the karyotypes (on the left), chromosomes are arranged in rows corresponding to genome origin: 1–14 C-genome, 15–28 A-genome, and 29–42 D-genome. White stars, arrows, and arrowheads denoted C-, A-, and D-chromosome regions, translocated to a different genome. Scale bars = 5 μm. (TIF 8552 kb)

Additional file 8: **Figure S8.** Fluorescent in situ hybridization (FISH) karyotyping of *Avena sativa*. Probes are AF226603_45bp (direct TET, red) for the C genome, pAs120a (biotin, Alexa 594; blue) for the A genome, and Ah-R31 (digoxigenin, FITC, green). On the right the DAPI image of chromosomes is shown in white, in the middle the same metaphase shows hybridization signal. Probes were amplified from different diploid species. a *A*. *brevis*. b *A. atlantica.* c *A. strigosa*. d *A. wiestii*. e *A. longiglumis*. f *A. hirtula*. In the karyotypes (on the left), chromosomes are arranged in rows corresponding to genome origin: 1–14 C-genome, 15–28 A-genome, and 29–42 D-genome. White stars, arrows, and arrowheads denoted C-, A-, and D-chromosome regions, translocated to a different genome. Additionally, white diamonds denote strong green band signal in Additional file 8: Figure S8e). Scale bars = 5 μm. (TIF 7444 kb)

Additional file 9: **Figure S9.** Fluorescent in situ hybridization (FISH) karyotyping of *Avena sativa*. Probes are AF226603_45bp (direct TET, red) for the C genome, pAs120a (biotin, Alexa 594; blue) for the A genome, and Ab-T148 (digoxigenin, FITC, green). On the right the DAPI image of chromosomes is shown in white, in the middle the same metaphase shows hybridization signal. Probes were amplified from different diploid species. a *A*. *brevis*. b *A. atlantica.* c *A. strigosa*. d *A. wiestii*. e *A. longiglumis*. f *A. hirtula*. In the karyotypes (on the left), chromosomes are arranged in rows corresponding to genome origin: 1–14 C-genome, 15–28 A-genome, and 29–42 D-genome. White stars, arrows, and arrowheads denoted C-, A-, and D-chromosome regions, translocated to a different genome. Additionally, white diamonds denote strong green band signal in Additional file 9: Figure S9c-e). Scale bars = 5 μm. (TIF 8041 kb)

Additional file 10: **Figure S10.** Fluorescent in situ hybridization (FISH) of *Avena sativa*. Chromosomal distribution of biotin labelled As_16mer43bp (red) and selected fluorescence in situ hybridization (FISH) probes labelling C-, A- and D-genome chromosomes of common oat (*Avena sativa*). a Digoxigenin labeled Ah-R52 (green) from *A. hirtula*. b Digoxigenin labeled As-R133 (green) from *A. longiglumis*. c Digoxigenin labeled Ast-R155 (green) from *A. wiestii*. d Digoxigenin labeled Ast-R176 (green) from *A. hirtula*. e Digoxigenin labeled Ah-T125 (green) from *A. wiestii*. f Digoxigenin labeled Ast-T125 (green) from *A. wiestii*. Scale bars = 5 μm. (TIF 8865 kb)

Additional file 11: **Figure S11.** rDNA sequence assembly and FISH. (a-b) Alignment of cluster contig sequences to assemble the 45S (a) and 5S rDNA (b) of *Avena sativa.* (c-e) FISH karyotyping of *A. sativa* with AF226603_45bp (direct TET, red), rDNA, and others. On the right the DAPI image of chromosomes is shown in white, in the middle the same metaphase shows hybridization signal. In the karyotypes (on the left), chromosomes are arranged in rows corresponding to genome origin: 1–14 C-genome, 15–28 A-genome, and 29–42 D-genome. White angle brackets and pigeons denoted 45S and 5S rDNA signals, respectively. c Digoxigenin labeled Ab-T148 (green) from *A. wiestii* and biotin labeled pTa71_45S (blue). d Digoxigenin labeled Ab-T148 (green) from *A. wiestii* and biotin labeled pTa794_5S (blue). e Digoxigenin labeled pTa794_5S (green) and biotin labeled pTa71_45S (blue) from *T. aestivum*. Scale bars = 5 μm. (TIF 4200 kb)

Additional file 12: **Figure S12.** Analysis of neighboring RepeatExplorer clusters. The interactive graphs showing selected clusters harboring FISH probes used in the present study (red circles) with 1st order neighbors with at least one connection (green circles). Connection between two neighboring clusters shown by grey and black lines along with similar read number. a 312CL82. b 312CL83. c 312CL119. d 312CL125. e 312CL133. f 312CL151. g 312CL153. h 289CL18. i 289CL19. j 289CL93. k 289CL105. l 289CL126. m 289CL145. n 289CL159. o 289CL166. p 299CL31. q 299CL52. r 315CL87. s 315CL116. t 315CL125. Clusters with at least one neighbor shown in circle. Remaining clusters 289CL148, 289CL187, 299CL118, 299CL125, 299CL126, 312CL175, 315CL155, 315CL171 and 315CL176 without any one neighbor. Black letters denote tandem repeats (b, c, f-g, j-k, m-o, s-t) and brown-red letters denote linear cluster graphs of non-tandem repeats (a, d-e, h-i, l, p-r). (JPG 457 kb)

Additional file 13: **Table S1.** Material used in this study. Source and origin, species name, authority, chromosome number and genome designation are given for the eight *Avena* samples used in this study. Repeat Cluster and spikelet figures are also listed. (DOCX 27 kb)

Additional file 14: **Table S2.** Statistics of whole-genome shotgun sequencing reads and RepeatExplorer analyses of *Avena* species. Haploid genome size and whole-genome shotgun sequencing reads statistics of four *Avena* species are given in (a) and RepeatExplorer summary data in (b). (DOCX 17 kb)

Additional file 15: **Table S3.** RepeatExplorer analyses of four *Avena* species. Statistics of cluster, read number and genome proportion of repetitive DNA composition (a) and the cluster (greater than or equal to 1.00% of genome) annotation by RepeatExplorer analyses (b) of four *Avena* species are given. (DOCX 40 kb)

Additional file 16: **Table S4.** The NCBI blast results of 821 clusters of four *Avena* species. Cluster and the longest contigs within, description, query coverage and identity are listed for each cluster. (DOCX 92 kb)

Additional file 17: **Table S5.** Repetitive DNA composition of genomes of four *Avena* species. Genome portion (as percent) is listed for each species. Families and subfamilies of Transposable elements Class I (retrotransposons) and Class II (DNA transposons), and Tandem repeats are also given. (DOCX 22 kb)

Additional file 18: **Table S6.** Oligonucleotides used to generate FISH probes. Primer pairs, annealing temperatures, the expected fragment sizes and lables for repeats identified from k-mer or RepeatExplorer analyses of four *Avena* species. (DOCX 23 kb)

Additional file 19: **Table S7.** RepeatExplorer characterization of selected repeat clusters of four *Avena* species. Characterization of repetitive fragments used as FISH probes from k-mer or RepeatExplorer analyses of *Avena* species. Genome proportion, domain hits, contig length and FISH figure references are listed. (DOCX 24 kb)

Additional file 20: **Table S8.** Genomic copy number and relative proportion of selected repeats of *Avena* species. PairedEnd fasta data were used to for “Map to Reference” tool in Geneious v.10.0.7 (Kearse et al., 2012). (DOCX 25 kb)

Additional file 21: **Table S9.** FISH signal distribution patterns on *Avena sativa* chromosomes using probes for selected repeats. Descriptions are based on analysis of 269 slides and 2353 metaphase (avg. 8 metaphases per slide) were analysed and chromosomes assigned to genomes C, A or D according to Sanz et al. (2010). Ten probes are C genome specific, one probe A genome specific, two probe D genome specific, five probes A and D genome specific and seven probes hybridized to all three genomes. (DOCX 25 kb)

Additional file 22: **Table S10.** The NCBI blast results of tandem repeats in Fig. 6b. Genome specificity, satellite family designation, monomer lengths and NCBI accession of twelve tandem repeats are listed. (DOCX 28 kb)

## Abbreviations

Ab: *Avena brevis*
Ah: *Avena hirtula*
As: *Avena sativa*
Ast: *Avena strigosa*
BLAST: Basic local alignment search tool
BSA: Bovine serum albumin
CGH: Comparative genomic hybridization
DAPI: 4′, 6-diamidino-2-phenylindole
EDTA: Ethylenediamine-tetraacetic acid
EnSpm: Enhancer suppressor mutator
FISH: Fluorescent in situ hybridization
FITC: Fluorescein isothiocyanate
GC: Guanine-cytosine
INT: Integrase
LINE: Long interspersed nuclear element
LTR: Long terminal repeat
NCBI: National Center for Biotechnology Information
NOR: nucleolus organizer region
PCR: Polymerase chain reaction
rDNA: Ribosomal DNA
RH: RnaseH
RT: Reverse transcriptase
satDNA: Satellite DNA
SDS: Sodium dodecyl sulphate
SINEs: Short interspersed nuclear element
SSC: Saline sodium citrate
TE: Transposable element
TET: Tetrachlorofluorescein
WGD: Whole genome duplication
