# Supplementary figures and images for: The repetitive DNA landscape in Avena (Poaceae): chromosome and genome evolution defined by major repeat classes in whole-genome sequence reads
Source: BMC Plant Biol. 2019 May 30;19:226. doi: 10.1186/s12870-019-1769-z (PMC6543597; doi:10.1186/s12870-019-1769-z)

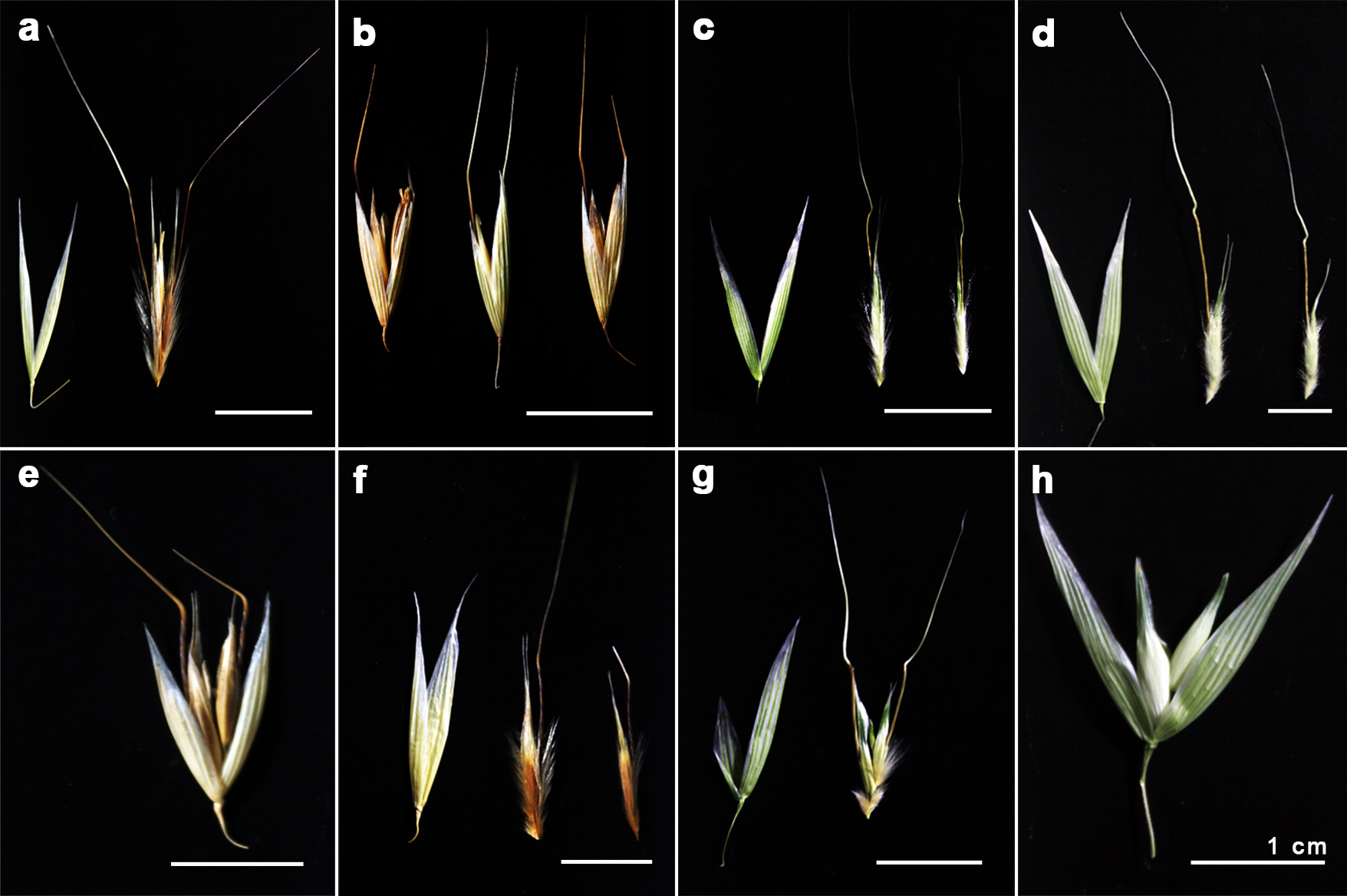

Supplement: Supplementary file 1 — Figure S1. Spikelet morphology of eight sampled Avena species. a A. atlantica: the dispersion units—the upper florets are attached to the lower floret and only the lower floret show disarticulation. b A. brevis: spikelets show persistent florets with bidenticulate lemma tips at maturity. c A. hirtula: a Mediterranean wild type with lemma bristles 6–10 mm. d A. longiglumis: 2–3 florets/spikelet, each floret is disarticulated; lemma back is covered with dense hairs. e A. strigosa: 2–3 florets/spikelet and persistent florets. f A. wiestii: desert and steppe wild type with lemma bristles 5–8 mm. g A. eriantha: glumes markedly unequal in size. h A. sativa: spikelets 1.5–4 cm with typically spread glumes at maturity. Scale bars = 1 cm. (TIF 4210 kb) [file 12870_2019_1769_MOESM1_ESM.tif]

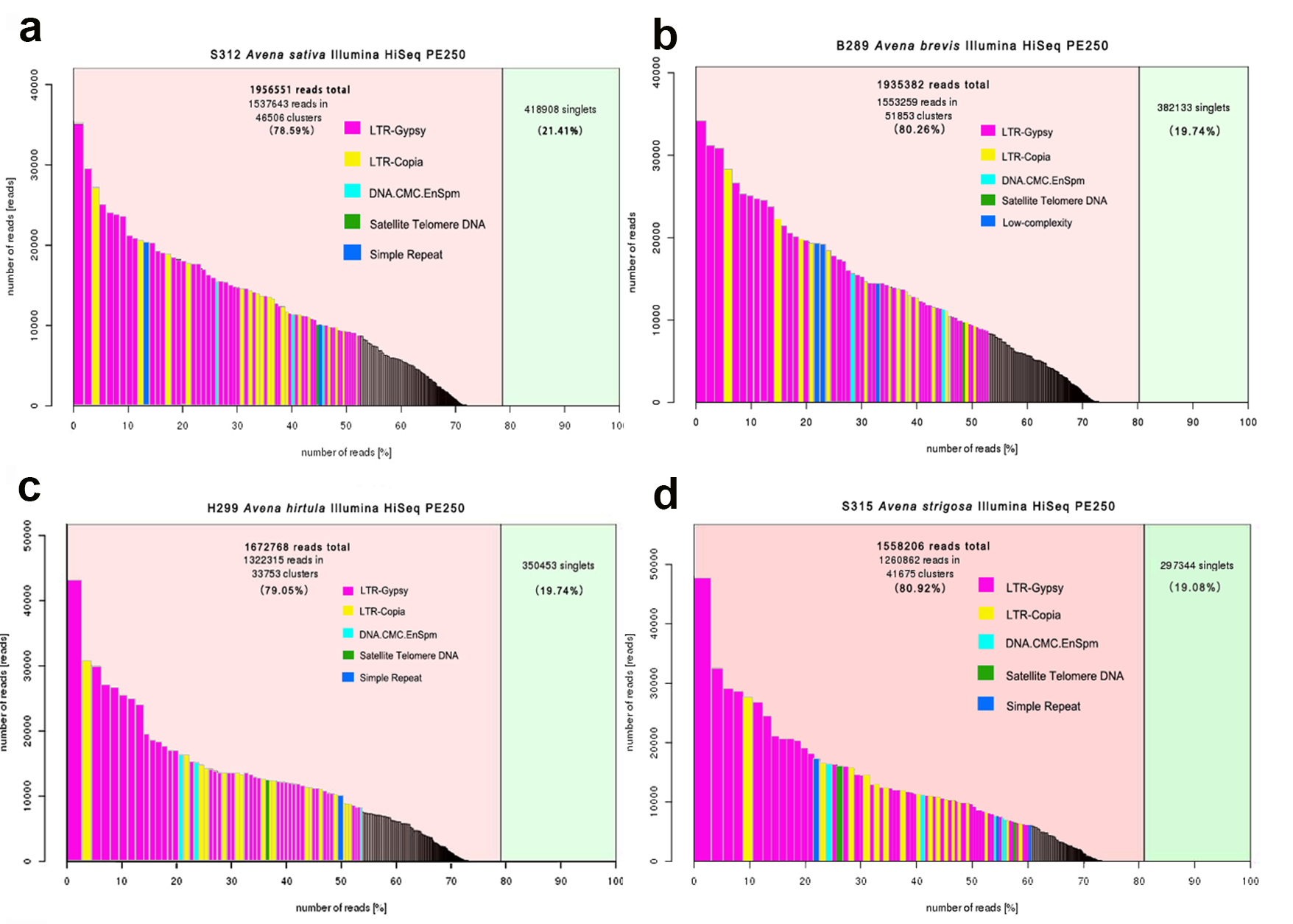

Supplement: Supplementary file 2 — Figure S2. Distribution of graph-based clusters. Hierarchical agglomeration of RepeatExplorer analyses of four Avena species genomes are shown. a A. sativa S312. b A. brevis B289. c A. hirtula. H299. d A. strigosa S135. Coloured bars denote clusters ≥0.01% of genome: x-axis denotes the cumulative read number percentage while y-axis denotes the read numbers in the clusters. Bars coloured according to the repeat types of cluster annotation (Additional files 15: Table S3). (TIF 1895 kb) [file 12870_2019_1769_MOESM2_ESM.tif]

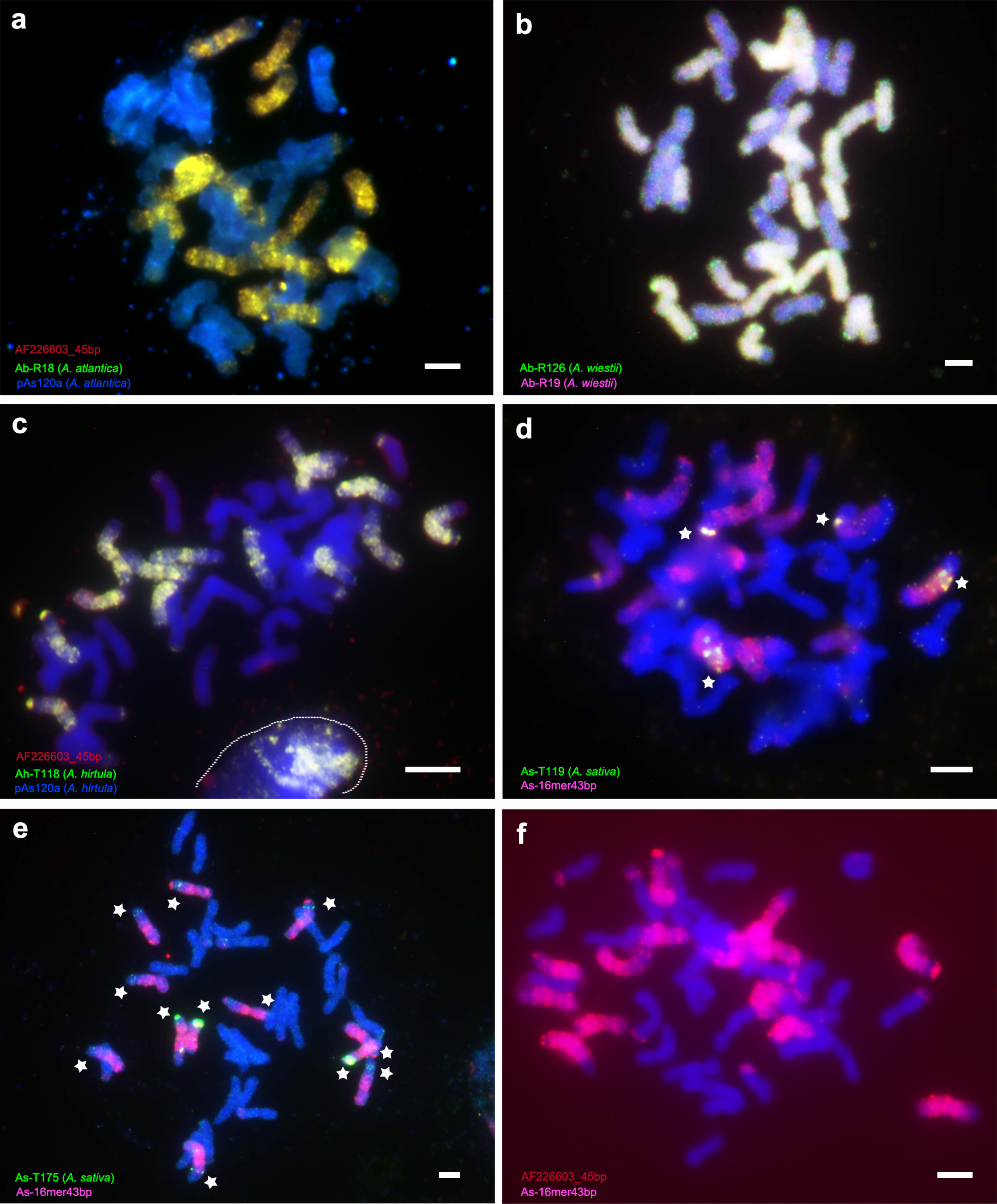

Supplement: Supplementary file 3 — Figure S3. Localization of selected C-genome specific repetitive sequences on Avena sativa metaphase chromosomes by multicolour FISH. Probe signals were captured individually with a black and white CCD camera and then pseudo-coloured to create overlaid images. For detailed description of signal distribution see Additional file 21: Table S9. a AF226603_45bp (hybridization sites displayed in red), Ab-R18 amplified from A. atlantica (green), and pAs120a from A. atlantica (blue). Note that overlapping signals of the red and green probe give yellow signals. b Ab-R19 (red) and Ab-R126 (green), counterstain DAPI (blue) shown on all chromosomes. Note that overlapping signals of the red and green probe appear white, but show several chromosome ends not labelled by either probe appearing blue or show green double dots. c AF226603_45bp (red), Ah-T118 from A. hirtula (green), and pAs120a from A. hirtula (blue). Overlapping signals of the red and green probe appear yellow and show non-uniform labelling of chromosomes. An interphase nucleus is visible at the bottom of the image. d As-T119 (green), double-dots (starred) appearing in yellow on top of the red signal of As_16mer43bp. DAPI fluorescence shown in blue.e As-T175 (green, double-dots) and As_16mer43bp (red) showing large blocks of hybridization signal on C genome chromosomes (starred). DAPI fluorescence shown in blue. f TET labeled AF226603_45bp (red), As_16mer43bp (pink), and DAPI (blue). Scale bars = 5 μm. (TIF 6648 kb) [file 12870_2019_1769_MOESM3_ESM.tif]

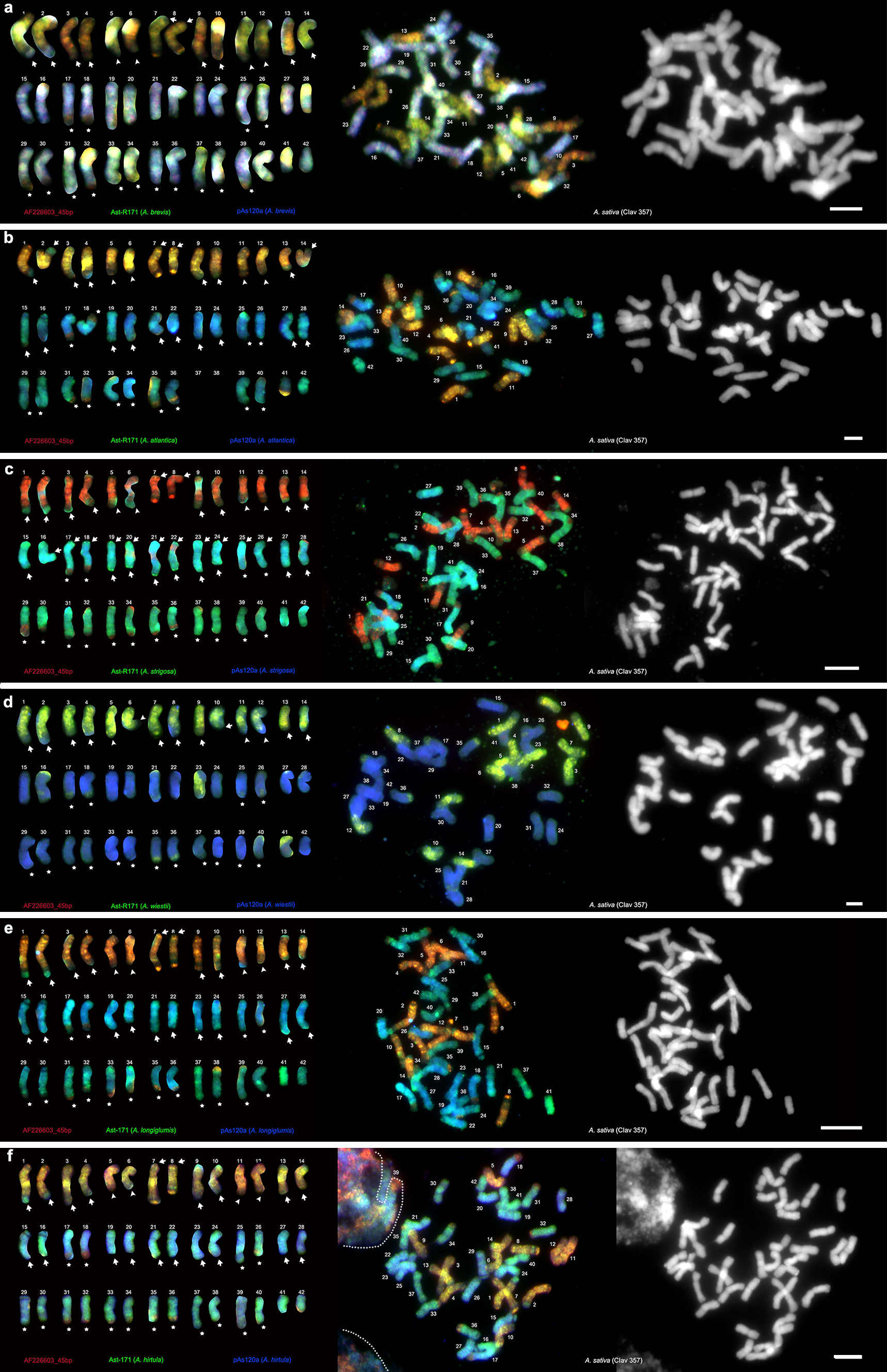

Supplement: Supplementary file 4 — Figure S4. Fluorescent in situ hybridization (FISH) karyotyping of Avena sativa. Probes are AF226603_45bp (direct TET, red) for the C genome, pAs120a (biotin, Alexa 594, blue) for the A genome, and Ast-R171 (digoxigenin, FITC, green) for the D genome. On the right the DAPI image of chromosomes is shown in white, in the middle the same metaphase shows hybridization signal from all three probes except in (d) where only AF226603_45bp in yellow and pAs120 in blue are visible. Probes were amplified from different diploid species. a A. brevis. b A. atlantica. c A. strigosa. d A. wiestii. e A. longiglumis. f A. hirtula. In the karyotypes (on the left), chromosomes are arranged in rows corresponding to genome origin: 1–14 C-genome, 15–28 A-genome, and 29–42 D-genome. White stars, arrows, and arrowheads denoted C-, A-, and D-chromosome regions, translocated to a different genome: there are C translocations on 12 D-chromosomes (29–40); A translocations on four C-chromosomes (5/6 & 11/12); D translocations on 10 A-chromosomes (15/16, 19–24 & 27/28). Scale bars = 5 μm. (TIF 7816 kb) [file 12870_2019_1769_MOESM4_ESM.tif]

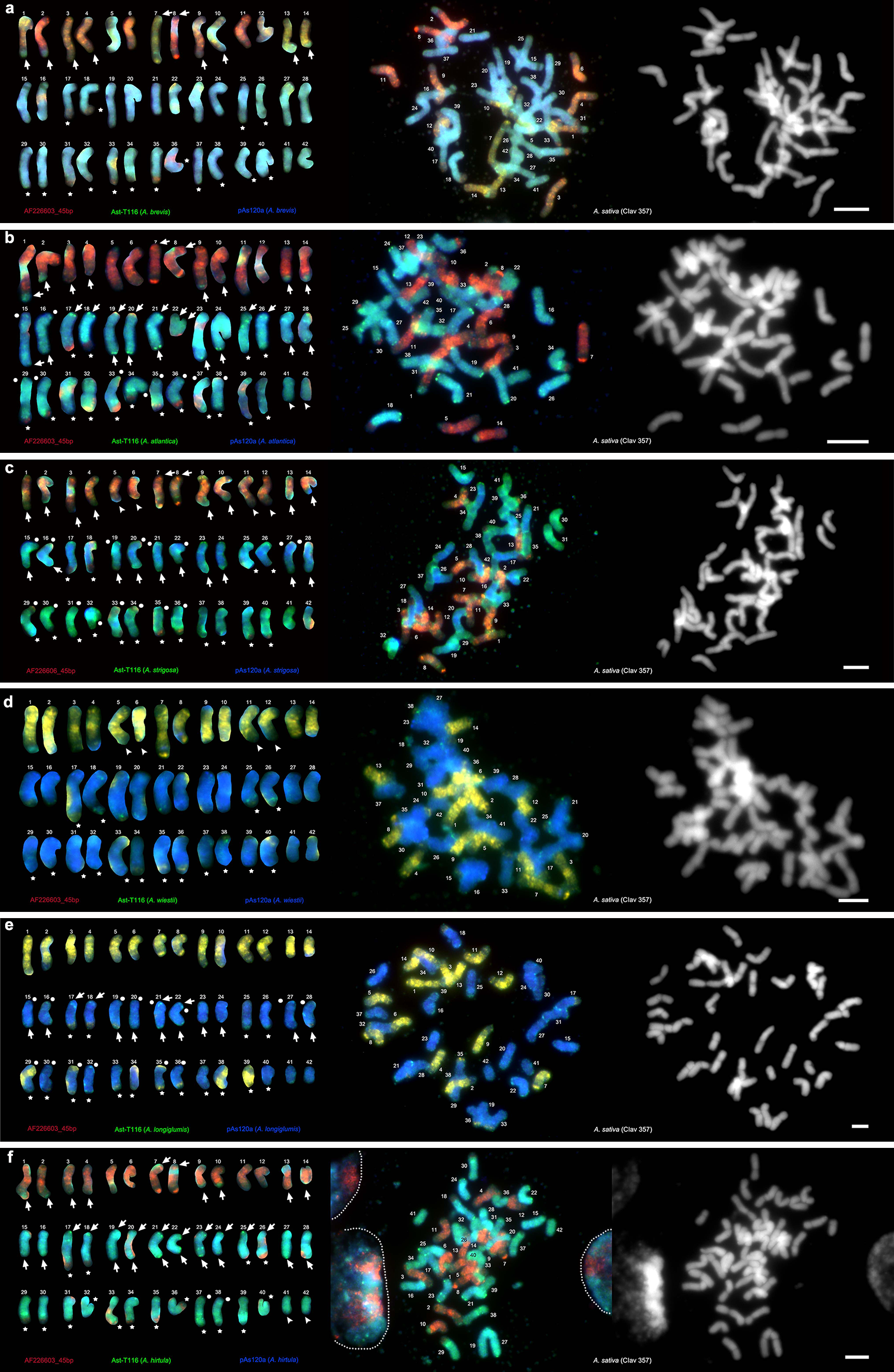

Supplement: Supplementary file 5 — Figure S5. Fluorescent in situ hybridization (FISH) karyotyping of Avena sativa. Probes are AF226603_45bp (direct TET, red) for the C genome, pAs120a (biotin, Alexa 594; blue) for the A genome, and Ast-T116 (digoxigenin, FITC, green) for the D genome. On the right the DAPI image of chromosomes is shown in white, in the middle the same metaphase shows hybridization signal from all three probes except in (d) and (e) where only AF226603_45bp in yellow and pAs120 in blue are visible. Probes were amplified from different diploid species. a A. brevis. b A. atlantica. c A. strigosa. d A. wiestii. e A. longiglumis. f A. hirtula. In the karyotypes (on the left), chromosomes are arranged in rows corresponding to genome origin: 1–14 C-genome, 15–28 A-genome, and 29–42 D-genome. White circles denote nucleolus organizer regions (NORs) signals. White stars, arrows, and arrowheads denoted C-, A-, and D-chromosome regions, translocated to a different genome: there are D translocations on ten C-chromosomes (1–4, 7–10 & 13/14); there are C translocations on 10 D-chromosomes (29–40); A translocations on four C-chromosomes (5/6 & 11/12); D translocations on 10 A-chromosomes (15/16, 19–24 & 27/28). Scale bars = 5 μm. (TIF 8170 kb) [file 12870_2019_1769_MOESM5_ESM.tif]

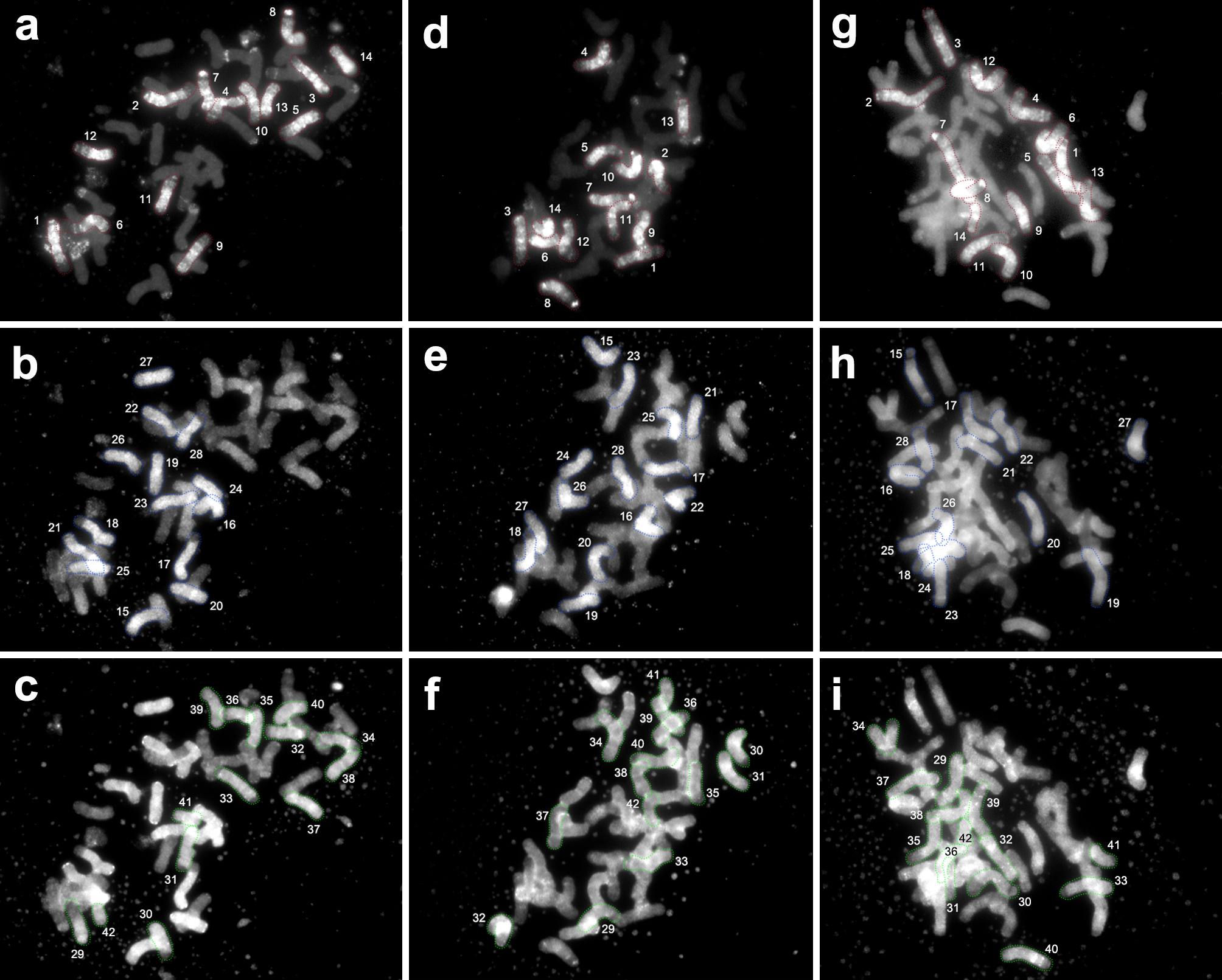

Supplement: Supplementary file 6 — Figure S6. Fluorescent in situ hybridization (FISH) of Avena sativa. Single channel images of far-red (a, d, g), pseudocoloured blue (b, e, h), and green (c, f, i) dotted lines circling C, A and D-genome chromosomes analysed by Image J v.1.51j8 for Figs. 4a (a-c), 4e (d-f) and 5c (g-i). (a-c) FISH probes AF226603_45bp (direct TET, red), pAs120a (biotin, Alexa 594; blue) and Ast-R171 (digoxigenin, FITC, green) and from A. strigosa: separation of the overlapped chromosomes (a) 6, (b) 21 & 25, 16, 23 & 24, and (c) 29 & 42 and 31 & 41 for Fig. 4d. (d-f) FISH probes AF226603_45bp (far red), Ast-T116 (green) and pAs120a (pseudocoloured blue) from A. strigosa: separation of the overlapped chromosomes (d) 3, 6, 12 & 14, (e) 18 & 27, and (f) 33 & 42 for Fig. 4e. (g-i) AF226603_45bp (far red), A/D-genome specific Ab-T166 (green), and pAs120a (pseudocoloured blue) from A. atlantica: separation of the overlapped chromosomes (g) 7, 8 & 14, (h) 18, 23, 24, 25 & 26, and (i) 31 & 36 for Fig. 5c. (TIF 6215 kb) [file 12870_2019_1769_MOESM6_ESM.tif]

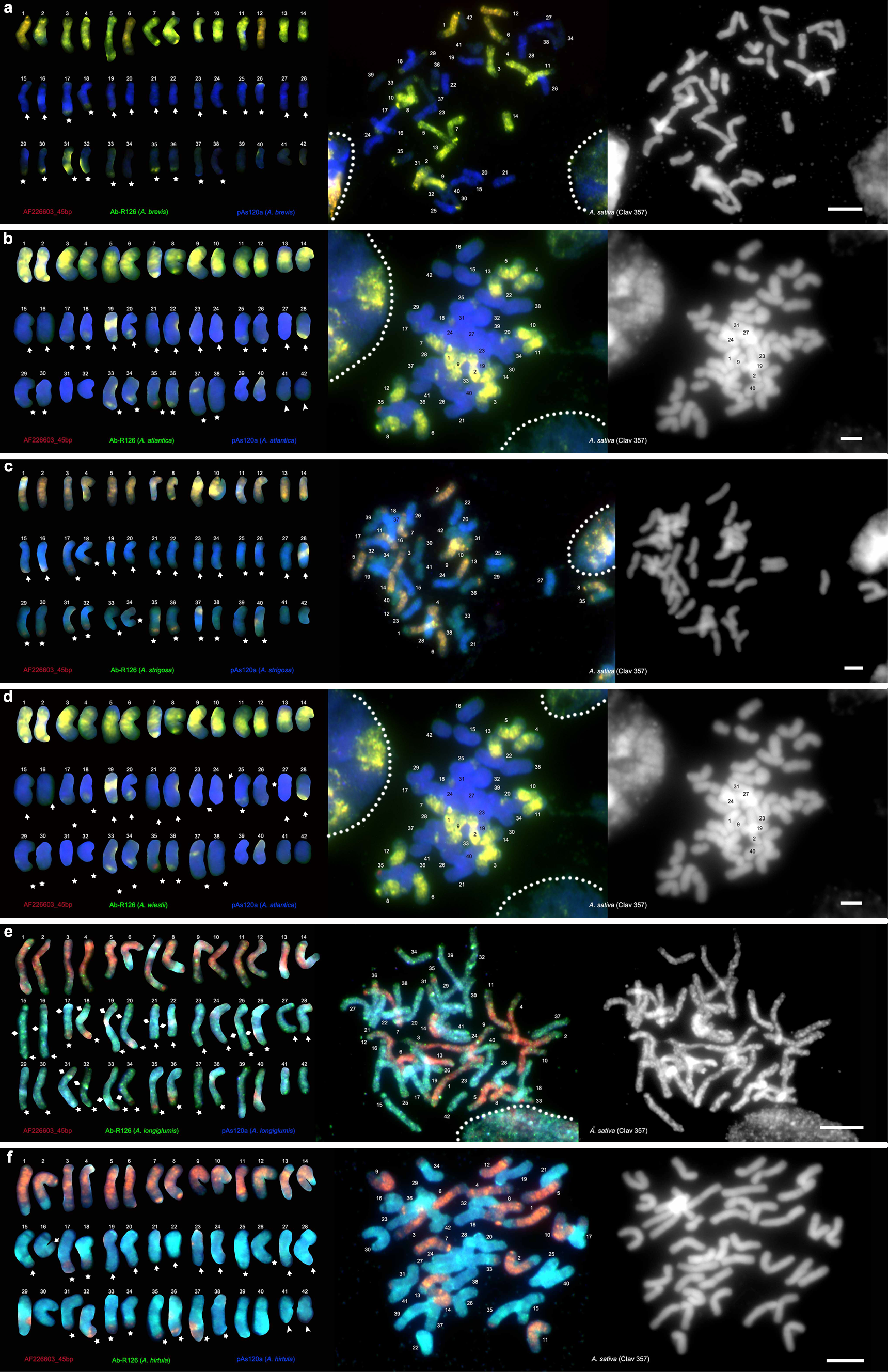

Supplement: Supplementary file 7 — Figure S7. Fluorescent in situ hybridization (FISH) karyotyping of Avena sativa. Probes are AF226603_45bp (direct TET, red) for the C genome, pAs120a (biotin, Alexa 594; blue) for the A genome, and Ab-R126 (digoxigenin, FITC, green). On the right the DAPI image of chromosomes is shown in white, in the middle the same metaphase shows hybridization signal. Probes were amplified from different diploid species. a A. brevis. b A. atlantica. c A. strigosa. d A. wiestii. e A. longiglumis. f A. hirtula. In the karyotypes (on the left), chromosomes are arranged in rows corresponding to genome origin: 1–14 C-genome, 15–28 A-genome, and 29–42 D-genome. White stars, arrows, and arrowheads denoted C-, A-, and D-chromosome regions, translocated to a different genome. Scale bars = 5 μm. (TIF 8552 kb) [file 12870_2019_1769_MOESM7_ESM.tif]

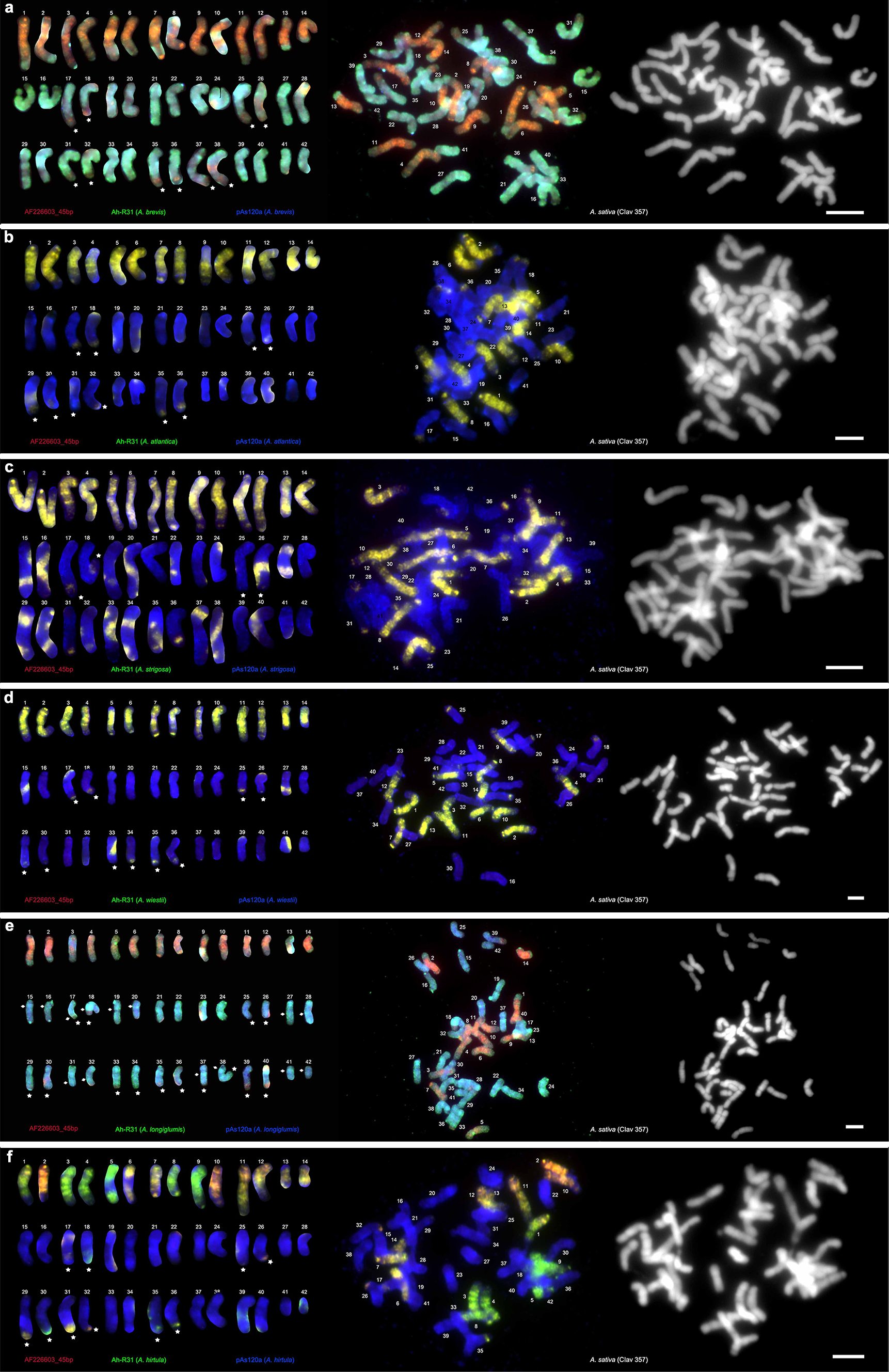

Supplement: Supplementary file 8 — Figure S8. Fluorescent in situ hybridization (FISH) karyotyping of Avena sativa. Probes are AF226603_45bp (direct TET, red) for the C genome, pAs120a (biotin, Alexa 594; blue) for the A genome, and Ah-R31 (digoxigenin, FITC, green). On the right the DAPI image of chromosomes is shown in white, in the middle the same metaphase shows hybridization signal. Probes were amplified from different diploid species. a A. brevis. b A. atlantica. c A. strigosa. d A. wiestii. e A. longiglumis. f A. hirtula. In the karyotypes (on the left), chromosomes are arranged in rows corresponding to genome origin: 1–14 C-genome, 15–28 A-genome, and 29–42 D-genome. White stars, arrows, and arrowheads denoted C-, A-, and D-chromosome regions, translocated to a different genome. Additionally, white diamonds denote strong green band signal in Additional file 8: Figure S8e). Scale bars = 5 μm. (TIF 7444 kb) [file 12870_2019_1769_MOESM8_ESM.tif]

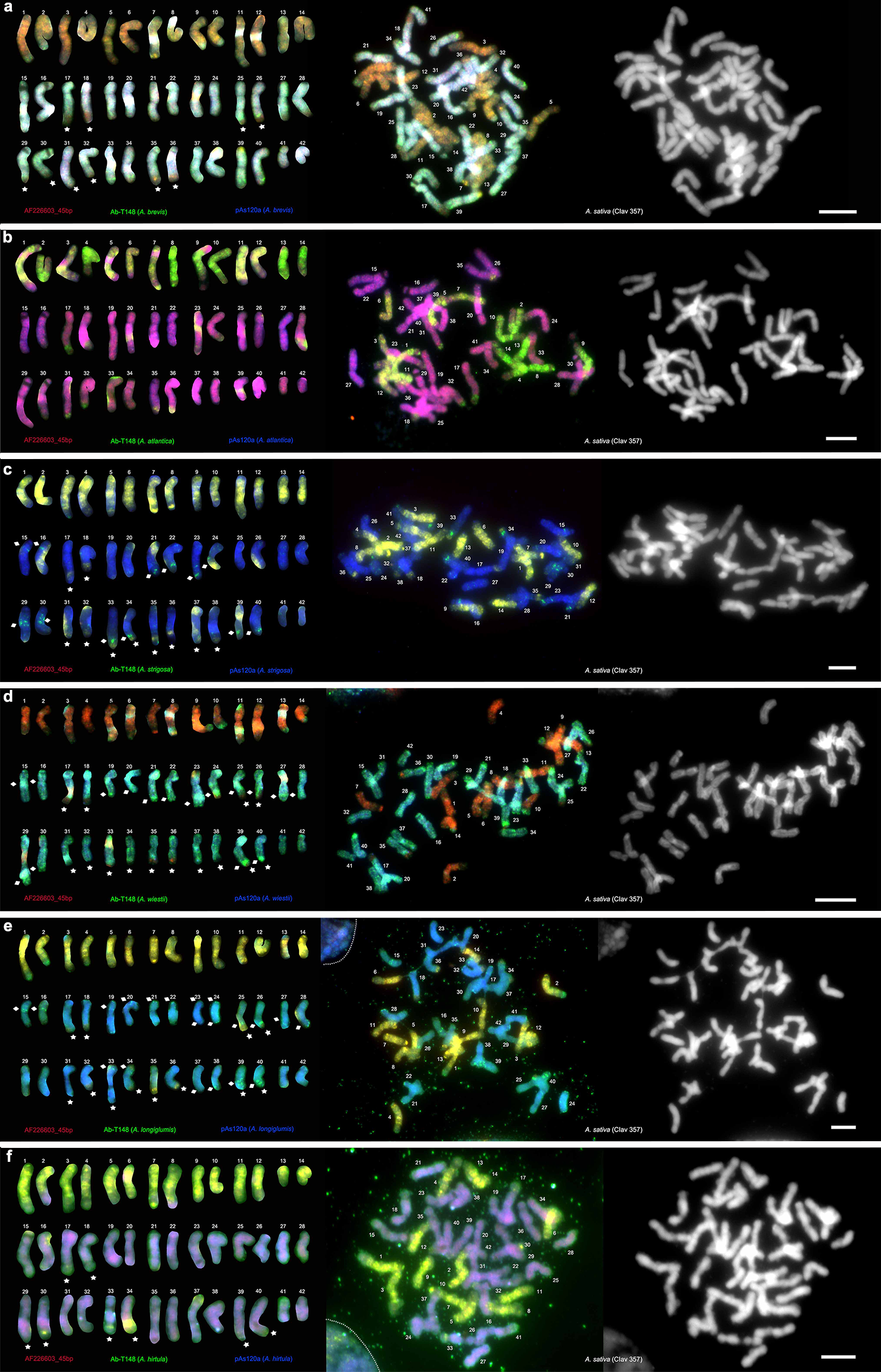

Supplement: Supplementary file 9 — Figure S9. Fluorescent in situ hybridization (FISH) karyotyping of Avena sativa. Probes are AF226603_45bp (direct TET, red) for the C genome, pAs120a (biotin, Alexa 594; blue) for the A genome, and Ab-T148 (digoxigenin, FITC, green). On the right the DAPI image of chromosomes is shown in white, in the middle the same metaphase shows hybridization signal. Probes were amplified from different diploid species. a A. brevis. b A. atlantica. c A. strigosa. d A. wiestii. e A. longiglumis. f A. hirtula. In the karyotypes (on the left), chromosomes are arranged in rows corresponding to genome origin: 1–14 C-genome, 15–28 A-genome, and 29–42 D-genome. White stars, arrows, and arrowheads denoted C-, A-, and D-chromosome regions, translocated to a different genome. Additionally, white diamonds denote strong green band signal in Additional file 9: Figure S9c-e). Scale bars = 5 μm. (TIF 8041 kb) [file 12870_2019_1769_MOESM9_ESM.tif]

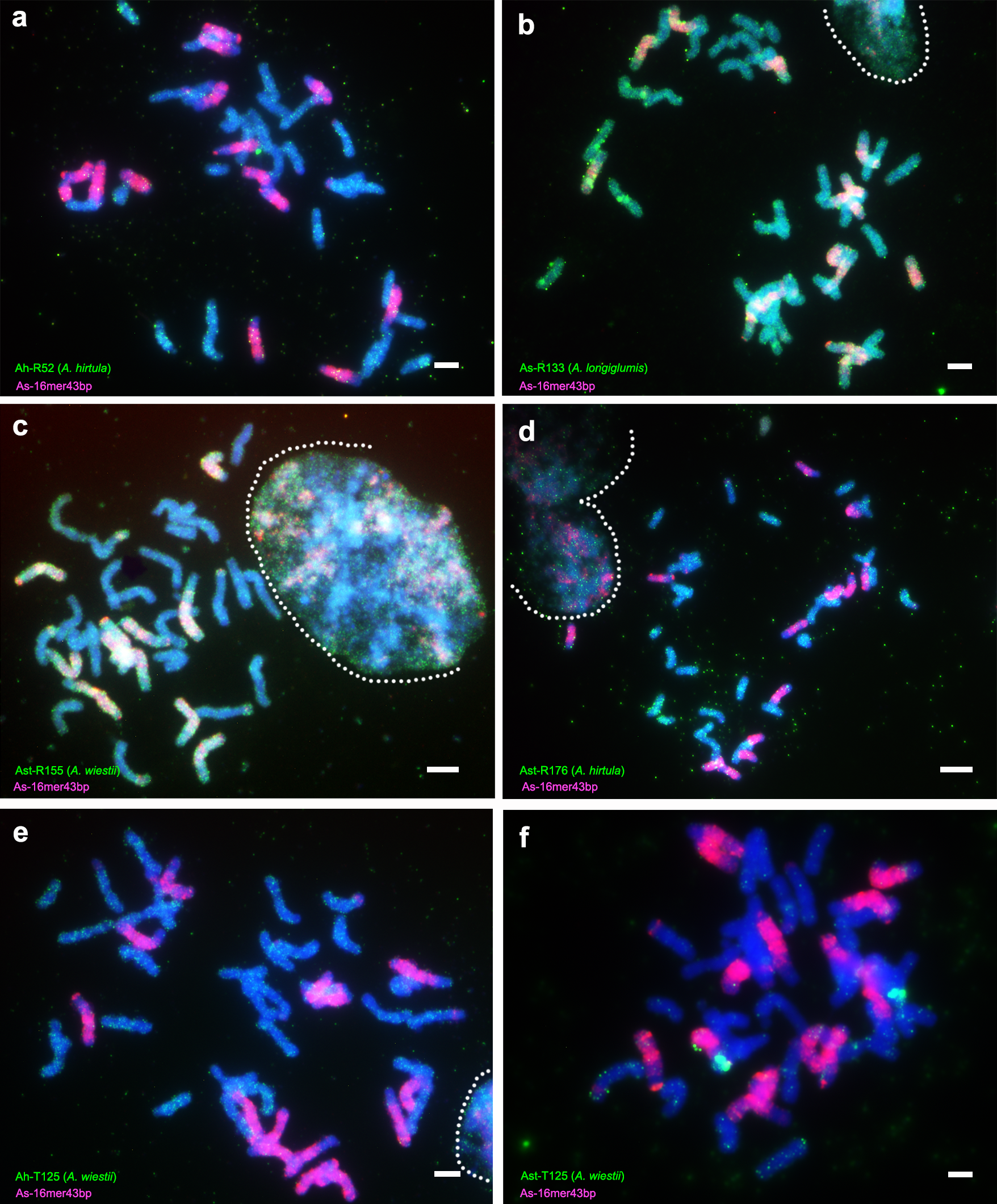

Supplement: Supplementary file 10 — Figure S10. Fluorescent in situ hybridization (FISH) of Avena sativa. Chromosomal distribution of biotin labelled As_16mer43bp (red) and selected fluorescence in situ hybridization (FISH) probes labelling C-, A- and D-genome chromosomes of common oat (Avena sativa). a Digoxigenin labeled Ah-R52 (green) from A. hirtula. b Digoxigenin labeled As-R133 (green) from A. longiglumis. c Digoxigenin labeled Ast-R155 (green) from A. wiestii. d Digoxigenin labeled Ast-R176 (green) from A. hirtula. e Digoxigenin labeled Ah-T125 (green) from A. wiestii. f Digoxigenin labeled Ast-T125 (green) from A. wiestii. Scale bars = 5 μm. (TIF 8865 kb) [file 12870_2019_1769_MOESM10_ESM.tif]

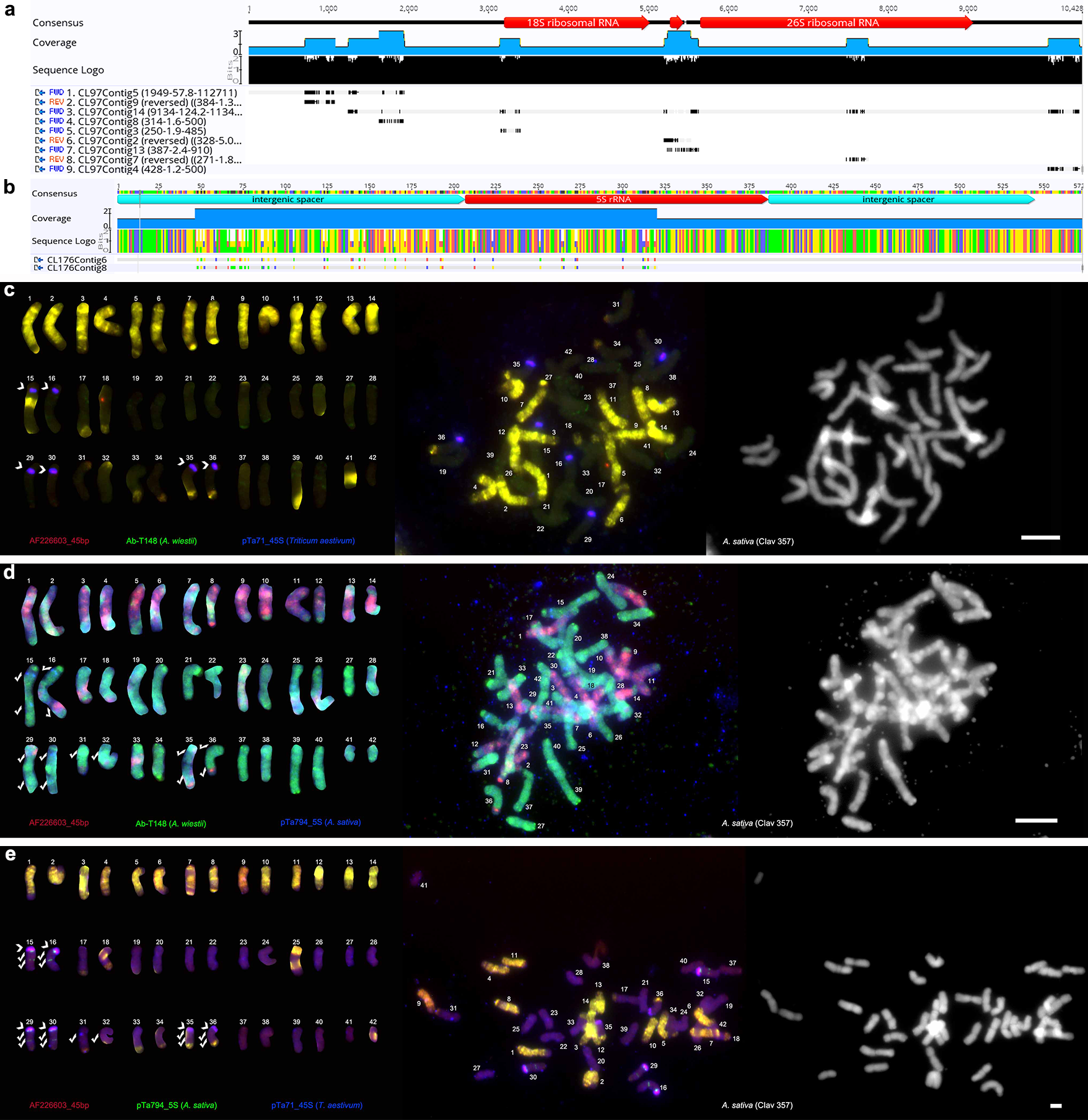

Supplement: Supplementary file 11 — Figure S11. rDNA sequence assembly and FISH. (a-b) Alignment of cluster contig sequences to assemble the 45S (a) and 5S rDNA (b) of Avena sativa. (c-e) FISH karyotyping of A. sativa with AF226603_45bp (direct TET, red), rDNA, and others. On the right the DAPI image of chromosomes is shown in white, in the middle the same metaphase shows hybridization signal. In the karyotypes (on the left), chromosomes are arranged in rows corresponding to genome origin: 1–14 C-genome, 15–28 A-genome, and 29–42 D-genome. White angle brackets and pigeons denoted 45S and 5S rDNA signals, respectively. c Digoxigenin labeled Ab-T148 (green) from A. wiestii and biotin labeled pTa71_45S (blue). d Digoxigenin labeled Ab-T148 (green) from A. wiestii and biotin labeled pTa794_5S (blue). e Digoxigenin labeled pTa794_5S (green) and biotin labeled pTa71_45S (blue) from T. aestivum. Scale bars = 5 μm. (TIF 4200 kb) [file 12870_2019_1769_MOESM11_ESM.tif]

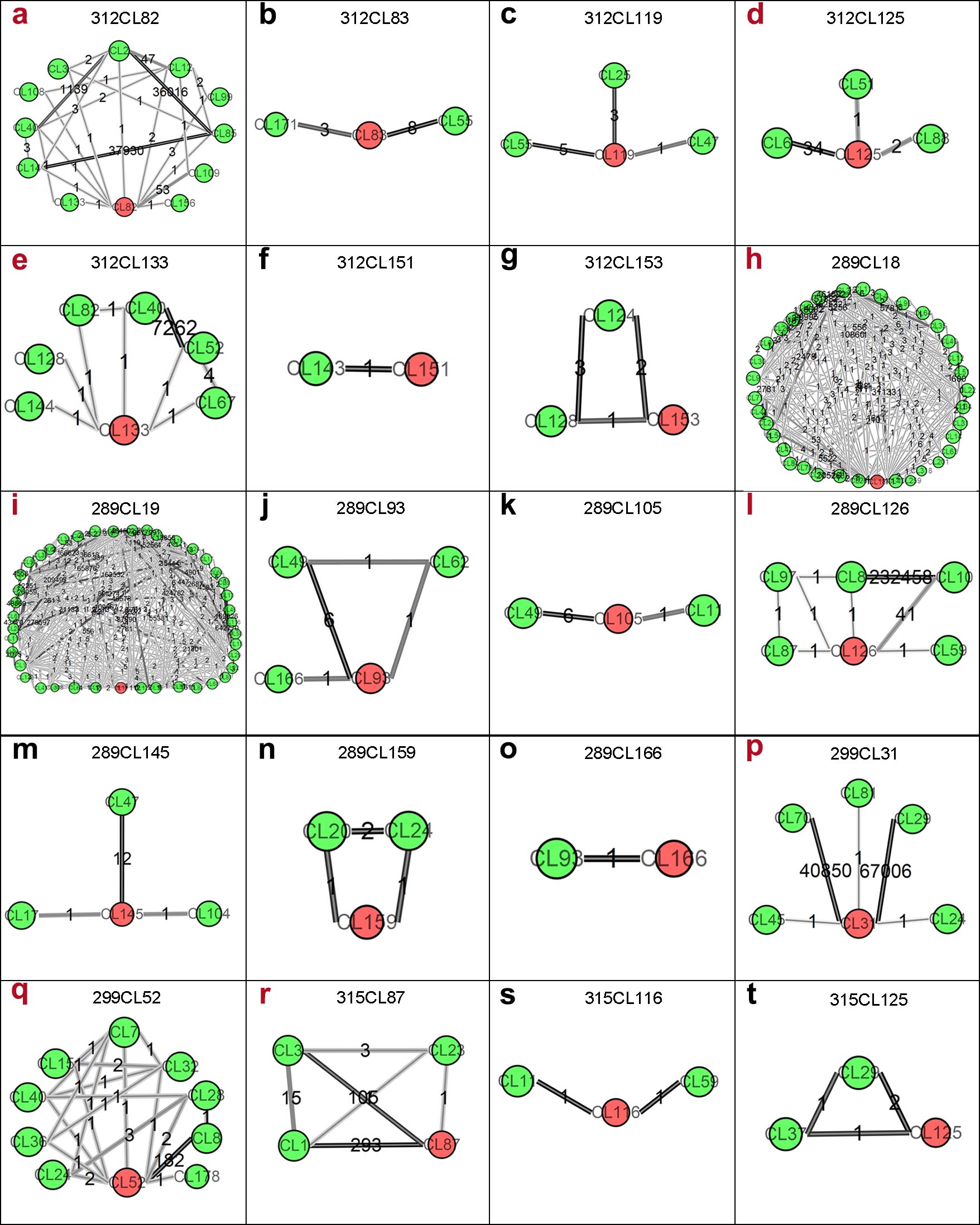

Supplement: Supplementary file 12 — Figure S12. Analysis of neighboring RepeatExplorer clusters. The interactive graphs showing selected clusters harboring FISH probes used in the present study (red circles) with 1st order neighbors with at least one connection (green circles). Connection between two neighboring clusters shown by grey and black lines along with similar read number. a 312CL82. b 312CL83. c 312CL119. d 312CL125. e 312CL133. f 312CL151. g 312CL153. h 289CL18. i 289CL19. j 289CL93. k 289CL105. l 289CL126. m 289CL145. n 289CL159. o 289CL166. p 299CL31. q 299CL52. r 315CL87. s 315CL116. t 315CL125. Clusters with at least one neighbor shown in circle. Remaining clusters 289CL148, 289CL187, 299CL118, 299CL125, 299CL126, 312CL175, 315CL155, 315CL171 and 315CL176 without any one neighbor. Black letters denote tandem repeats (b, c, f-g, j-k, m-o, s-t) and brown-red letters denote linear cluster graphs of non-tandem repeats (a, d-e, h-i, l, p-r). (JPG 457 kb) [file 12870_2019_1769_MOESM12_ESM.jpg]
