# Supplementary material for: The repetitive DNA landscape in Avena (Poaceae): chromosome and genome evolution defined by major repeat classes in whole-genome sequence reads
Source: BMC Plant Biol. 2019 May 30;19:226. doi: 10.1186/s12870-019-1769-z (PMC6543597; doi:10.1186/s12870-019-1769-z)
Supplement: Supplementary file 13 — Table S1. Material used in this study. Source and origin, species name, authority, chromosome number and genome designation are given for the eight Avena samples used in this study. Repeat Cluster and spikelet figures are also listed. (DOCX 27 kb) [file 12870_2019_1769_MOESM13_ESM.docx]

Table S1. Material used in this study.

| Taxa | Voucher (Source) | Origin country | Cluster | Purpose (NCBI accession number) | Spikelet figure |
| --- | --- | --- | --- | --- | --- |
| *Avena atlantica* B.R. Baum & Fedak  (2*x* = 14; AA) | Liu 437 (PI 657294; IBSC) | Morocco: Ain Aouda | - | FISH | S1a |
| *A. brevis* Roth  (2*x* = 14; AA) | Liu 289 (CN 1979; IBSC) | Canada: Ontario | 214 | WGS  (SRR6056491) |  |
|  | Liu 263 (CIav 1783, IBSC) | Germany: Lower Saxony | - | FISH | S1b |
| *A. hirtula* Lag.  (2*x* = 14; AA) | Liu 299 (PI 657464; IBSC) | Morocco: Al Houceima | 198 | WGS  (SRR6056492) |  |
|  | Liu 268 (PI 657464; IBSC) | Morocco: Al Houceima | - | FISH | S1c |
| *A. longiglumis* Durieu  (2*x* = 14; AA) | Liu 438 (PI 657389; IBSC) | Morocco: Moulay Bousselham | - | FISH | S1d |
| *A. strigosa* Schreb.  (2*x* = 14; AA) | Liu 315 (CN 21993; IBSC) | Portugal: - | 195 | WGS  (SRR6056490) |  |
|  | Liu 436 (CIav 1782; IBSC) | Russian Federation: Leningrad | - | FISH | S1e |
| *A. wiestii* Steud.  (2*x* = 14; AA) | Liu 439 (PI 657352; IBSC) | Morocco: Ain Aouda | - | FISH | S1f |
| *A. eriantha* Durieu  (2*x* = 14; CC) | Liu 435 (PI 657575; IBSC) | Morocco: Ain Leuh | - | FISH | S1g |
| *A. sativa* L.  (6*x* = 42; AACCDD) | Liu 312 (PI 51385; IBSC) | Spain: Soria | 214 | WGS  (SRR6056489) |  |
|  | Liu 440 (CIav 357; IBSC) | Greece: Thrace | - | FISH | S1h |

Taxa: Chromosome numbers based on http://mobot.mobot.org/W3T/Search/ipcn2.html; Genome assignment based on Liu *et al.* (2017). Voucher: CN, Plant Gene Resources at Saskatchewan, Canada; PI or CIav, Germplasm Resources Information Network of United States Department of Agriculture at Beltsville, USA; IBSC, South China Botanical Garden Herbarium. Cluster: -, unavailable. Purpose: FISH, fluorescence *in situ* hybridization; WGS, whole-genome shotgun sequencing.
