# Supplementary material for: The repetitive DNA landscape in Avena (Poaceae): chromosome and genome evolution defined by major repeat classes in whole-genome sequence reads
Source: BMC Plant Biol. 2019 May 30;19:226. doi: 10.1186/s12870-019-1769-z (PMC6543597; doi:10.1186/s12870-019-1769-z)
Supplement: Supplementary file 14 — Table S2. Statistics of whole-genome shotgun sequencing reads and RepeatExplorer analyses of Avena species. Haploid genome size and whole-genome shotgun sequencing reads statistics of four Avena species are given in (a) and RepeatExplorer summary data in (b). (DOCX 17 kb) [file 12870_2019_1769_MOESM14_ESM.docx]

**Table S2.** Statistics of whole-genome shotgun sequencing reads and RepeatExplorer analyses of *Avena* species.

1. Whole-genome shotgun sequencing reads statistics of four *Avena* species.

| Species | S312_*A. sativa* | S289_*A. brevis* | H299_*A. hirtula* | S315_*A. strigosa* |
| --- | --- | --- | --- | --- |
| Chromosome number | 2*n* = 6*x* = 42 | 2*n* = 2*x* =14 | 2*n* = 2*x* =14 | 2*n* = 2*x* =14 |
| Mean 2C× value ± SD (pg) | 25.70 ± 0.40 | 8.98 ± 0.25 | 9.08 ± 0.11 | 9.07 ± 0.22 |
| Haploid genome size (Mbp/1C) | 12567.30 | 4391.22 | 4440.12 | 4435.23 |
| NCBI accession number | SRR6056489 | SRR6056491 | SRR6056492 | SRR6056490 |
| Whole genome sequenced read number | 230,209,646 | 120,733,742 | 122,952,144 | 125,269,236 |
| Sequenced read length (bp) | 250 | 250 | 250 | 250 |
| PairedEnd nucleotides | 57,552,411,500 | 30,183,435,500 | 30,738,036,000 | 31,317,309,000 |
| GC-content of whole genome sequence | 43.27% | 43.25% | 43.19% | 43.21% |
| Illumina coverage | 4.58 × | 6.87 × | 6.92 × | 7.06 × |
| Raw sequences (GB) | 66.1 | 34.6 | 35.3 | 35.9 |

1. RepeatExplorer analyses data of four *Avena* species.

| Species | S312_*A. sativa* | S289_*A. brevis* | H299_*A. hirtula* | S315_*A. strigosa* |
| --- | --- | --- | --- | --- |
| Subset genome size upload to Repeat Explorer (GB) | 1.72 | 2.87 | 2.87 | 1.72 |
| Read number upload to RepeatExplorer analysis | 4,000,000 | 10,000,000 | 10,000,000 | 4,000,000 |
| Genome percentage upload to RepeatExplorer analysis | 2.60% | 8.29% | 8.13% | 4.79% |
| Number of clusters ≥ 0.01% of genome | 214 | 214 | 198 | 195 |
| Number of similarity hits | 669,648 | 674,159 | 602,950 | 549,442 |
