# Supplementary material for: The repetitive DNA landscape in Avena (Poaceae): chromosome and genome evolution defined by major repeat classes in whole-genome sequence reads
Source: BMC Plant Biol. 2019 May 30;19:226. doi: 10.1186/s12870-019-1769-z (PMC6543597; doi:10.1186/s12870-019-1769-z)
Supplement: Supplementary file 15 — Table S3. RepeatExplorer analyses of four Avena species. Statistics of cluster, read number and genome proportion of repetitive DNA composition (a) and the cluster (greater than or equal to 1.00% of genome) annotation by RepeatExplorer analyses (b) of four Avena species are given. (DOCX 40 kb) [file 12870_2019_1769_MOESM15_ESM.docx]

Table S3. RepeatExplorer analyses of four *Avena* species.

1. Repetitive DNA classification, composition of clusters, read numbers and genome proportions.

| Species | S312_*A. sativa* | | | B289_*A. brevis* | | | H299_*A. hirtula* | | | S315_*A. strigosa* | | |
| --- | --- | --- | --- | --- | --- | --- | --- | --- | --- | --- | --- | --- |
| Repetitive DNA composition | Cluster number | Read number | Genome proportion | Cluster number | Read number | Genome proportion | Cluster number | Read number | Genome proportion | Cluster number | Read number | Genome proportion |
| LTR.Gypsy | 94 | 861821 | 44.04% | 94 | 828977 | 43.04% | 78 | 704454 | 41.88% | 76 | 671843 | 42.31% |
| LTR.Copia | 41 | 312964 | 16.00% | 36 | 308563 | 15.74% | 38 | 294330 | 17.39% | 39 | 271823 | 17.24% |
| LTR.Cassandra | 1 | 1210 | 0.06% | 1 | 1906 | 0.10% | 1 | 726 | 0.04% | 1 | 955 | 0.06% |
| LINE.L1 | 7 | 6669 | 0.32% | 6 | 6940 | 0.32% | 7 | 8120 | 0.45% | 6 | 6719 | 0.42% |
| DNA.PIF.Harbinger | 1 | 535 | 0.03% | 1 | 855 | 0.04% | 1 | 832 | 0.05% | 1 | 536 | 0.03% |
| DNA-CMC.EnSpm | 25 | 77952 | 3.98% | 23 | 76671 | 3.96% | 25 | 83294 | 4.98% | 26 | 80940 | 5.20% |
| DNA.MULE.MuDR | 8 | 14428 | 0.74% | 8 | 18019 | 0.93% | 9 | 9700 | 0.58% | 12 | 6079 | 0.39% |
| DNA.TcMar.Stowaway | 5 | 15440 | 0.79% | 4 | 9547 | 0.49% | 1 | 4652 | 0.28% | 4 | 3974 | 0.26% |
| RC.Helitron | 1 | 1116 | 0.06% | 0 | 0 | 0.00% | 1 | 2326 | 0.14% | 1 | 2706 | 0.17% |
| Satellite | 12 | 35178 | 1.80% | 16 | 33761 | 1.74% | 18 | 39725 | 2.38% | 13 | 40709 | 2.61% |
| rDNA | 2 | 12507 | 0.64% | 6 | 14138 | 0.73% | 1 | 7382 | 0.44% | 3 | 7046 | 0.45% |
| Low_complexity | 1 | 226 | 0.01% | 7 | 57166 | 2.95% | 2 | 14578 | 0.76% | 0 | 0 | 0.00% |
| Simple_repeat | 13 | 48461 | 2.48% | 3 | 8145 | 0.42% | 16 | 45974 | 2.75% | 11 | 37659 | 2.41% |
| Unknown | 3 | 1106 | 0.06% | 11 | 22241 | 1.12% | 0 | 0 | 0.00% | 2 | 401 | 0.03% |
| Total | 214 | 1389613 | 71.96% | 214 | 1386929 | 71.62% | 198 | 1216093 | 73.60% | 195 | 1131390 | 72.90% |
| Average | 1 | 6494 | 0.34% | 1 | 6481 | 0.34% | 1 | 6142 | 0.37% | 1 | 5802 | 0.37% |

1. Clusters greater than or equal to 1.00% of genome, annotation in RepeatExplorer.

| Species | Cluster | Read number | Genome proportion (%) | RepeatMasker annotation |
| --- | --- | --- | --- | --- |
| *Avena sativa* L. (312) | CL1 | 35054 | 1.79 | LTR.Gypsy (14746hits, 21.4%) |
|  | CL2 | 29549 | 1.51 | LTR.Gypsy (12613hits, 22.4%) |
|  | CL3 | 27162 | 1.39 | LTR.Copia (25510hits, 78.6%) |
|  | CL4 | 25005 | 1.28 | LTR.Gypsy (17388hits, 63.4%) |
|  | CL5 | 23970 | 1.23 | LTR.Gypsy (20158hits, 56.5%) |
|  | CL6 | 23663 | 1.21 | LTR.Gypsy (22hits, 0.0242%) |
|  | CL7 | 23483 | 1.20 | LTR.Gypsy (85hits, 0.0746%) |
|  | CL8 | 20949 | 1.07 | LTR.Gypsy (9476hits, 30.6%) |
|  | CL9 | 20795 | 1.06 | LTR.Gypsy (16918hits, 62.9%) |
|  | CL10 | 20529 | 1.05 | LTR.Copia (32hits, 0.0472%) |
|  | CL11 | 20339 | 1.04 | Simple_repeat (762hits, 0.483%) |
|  | CL12 | 20184 | 1.03 | LTR.Gypsy (11208hits, 24.8%) |
| Total | 12 | 290682 | 14.86 | - |
| Average | 1 | 24223 | 1.24 | - |
| *A. brevis* Roth (289) | [CL1](file:///D:\2016\2016访问生活\Lab%20Works\Primer%20for%20Avena\Liu289_xaa_HTML\CL0001\cluster.html) | 33976 | 1.76 | LTR.Gypsy (14780hits, 22.4%) |
|  | [CL2](file:///D:\2016\2016访问生活\Lab%20Works\Primer%20for%20Avena\Liu289_xaa_HTML\CL0002\cluster.html) | 31164 | 1.61 | LTR.Gypsy (10456hits, 22%) |
|  | [CL3](file:///D:\2016\2016访问生活\Lab%20Works\Primer%20for%20Avena\Liu289_xaa_HTML\CL0003\cluster.html) | 30819 | 1.59 | LTR.Gypsy (20668hits, 35.3%) |
|  | [CL4](file:///D:\2016\2016访问生活\Lab%20Works\Primer%20for%20Avena\Liu289_xaa_HTML\CL0004\cluster.html) | 28108 | 1.45 | LTR.Copia (26225hits, 77.6%) |
|  | [CL5](file:///D:\2016\2016访问生活\Lab%20Works\Primer%20for%20Avena\Liu289_xaa_HTML\CL0005\cluster.html) | 26454 | 1.37 | LTR.Gypsy (5744hits, 10.2%) |
|  | [CL6](file:///D:\2016\2016访问生活\Lab%20Works\Primer%20for%20Avena\Liu289_xaa_HTML\CL0006\cluster.html) | 25142 | 1.30 | LTR.Gypsy (22466hits, 75.2%) |
|  | [CL7](file:///D:\2016\2016访问生活\Lab%20Works\Primer%20for%20Avena\Liu289_xaa_HTML\CL0007\cluster.html) | 25072 | 1.30 | LTR.Gypsy (16898hits, 46.6%) |
|  | [CL8](file:///D:\2016\2016访问生活\Lab%20Works\Primer%20for%20Avena\Liu289_xaa_HTML\CL0008\cluster.html) | 24554 | 1.27 | LTR.Gypsy (100hits, 0.0851%) |
|  | [CL9](file:///D:\2016\2016访问生活\Lab%20Works\Primer%20for%20Avena\Liu289_xaa_HTML\CL0009\cluster.html) | 24525 | 1.27 | LTR.Gypsy (16891hits, 61.9%) |
|  | [CL10](file:///D:\2016\2016访问生活\Lab%20Works\Primer%20for%20Avena\Liu289_xaa_HTML\CL0010\cluster.html) | 23623 | 1.22 | LTR.Gypsy (33hits, 0.0369%) |
|  | [CL11](file:///D:\2016\2016访问生活\Lab%20Works\Primer%20for%20Avena\Liu289_xaa_HTML\CL0011\cluster.html) | 21991 | 1.14 | LTR.Copia (12337hits, 41.3%) |
|  | [CL12](file:///D:\2016\2016访问生活\Lab%20Works\Primer%20for%20Avena\Liu289_xaa_HTML\CL0012\cluster.html) | 21399 | 1.11 | LTR.Gypsy (14544hits, 57.3%) |
|  | [CL13](file:///D:\2016\2016访问生活\Lab%20Works\Primer%20for%20Avena\Liu289_xaa_HTML\CL0013\cluster.html) | 20421 | 1.06 | LTR.Gypsy (11381hits, 25.5%) |
|  | [CL14](file:///D:\2016\2016访问生活\Lab%20Works\Primer%20for%20Avena\Liu289_xaa_HTML\CL0014\cluster.html) | 20073 | 1.04 | LTR.Gypsy (7514hits, 30%) |
|  | [CL15](file:///D:\2016\2016访问生活\Lab%20Works\Primer%20for%20Avena\Liu289_xaa_HTML\CL0015\cluster.html) | 19607 | 1.01 | LTR.Copia (27hits, 0.0397%) |
|  | [CL16](file:///D:\2016\2016访问生活\Lab%20Works\Primer%20for%20Avena\Liu289_xaa_HTML\CL0016\cluster.html) | 19448 | 1.00 | LTR.Gypsy (16777hits, 62.2%) |
| Total | 16 | 396376 | 20.50 | - |
| Average | 1 | 24773 | 1.28 | - |
| *A. hirtula* Lag. (299) | CL1 | 43093 | 2.58 | LTR.Gypsy (19325hits, 22.7%) |
|  | CL2 | 30604 | 1.83 | LTR.Copia (27850hits, 76.6%) |
|  | CL3 | 29872 | 1.79 | LTR.Gypsy (23413hits, 71.2%) |
|  | CL4 | 26957 | 1.61 | LTR.Gypsy (3424hits, 3.52%) |
|  | CL5 | 26613 | 1.59 | LTR.Gypsy (8401hits, 18.7%) |
|  | CL6 | 25484 | 1.52 | LTR.Gypsy (3082hits, 4.16%) |
|  | CL7 | 24868 | 1.49 | LTR.Gypsy (11792hits, 22.2%) |
|  | CL8 | 23930 | 1.43 | LTR.Gypsy. (1770hits, 3.41%) |
|  | CL9 | 19263 | 1.15 | LTR.Gypsy (13618hits, 46.1%) |
|  | CL10 | 18539 | 1.11 | Simple_repeat (278hits, 0.216%) |
|  | CL11 | 18158 | 1.09 | LTR.Gypsy (2591hits, 8.21%) |
|  | CL12 | 17453 | 1.04 | LTR.Gypsy (8015hits, 36.8%) |
|  | CL13 | 16949 | 1.01 | LTR.Gypsy (3925hits, 12.3%) |
|  | CL14 | 16915 | 1.01 | LTR.Gypsy (8801hits, 44.6%) |
| Total | 14 | 338698 | 20.25 | - |
| Average | 1 | 24193 | 1.45% | - |
| *A. strigosa* Schreb. (315) | CL1 | 47321 | 3.04 | LTR.Gypsy (17828hits, 19%) |
|  | CL2 | 32481 | 2.08 | LTR.Gypsy (15290hits, 27%) |
|  | CL3 | 28968 | 1.86 | LTR.Gypsy (3644hits, 3.65%) |
|  | CL4 | 28557 | 1.83 | LTR.Gypsy (21550hits, 68.7%) |
|  | CL5 | 27460 | 1.76 | LTR.Copia (26308hits, 79.9%) |
|  | CL6 | 26698 | 1.71 | LTR.Gypsy (1199hits, 1.8%) |
|  | CL7 | 24428 | 1.57 | LTR.Gypsy (16800hits, 47%) |
|  | CL8 | 20978 | 1.35 | LTR.Gypsy (9720hits, 21.6%) |
|  | CL9 | 20533 | 1.32 | LTR.Gypsy (13956hits, 51.5%) |
|  | CL10 | 20527 | 1.32 | LTR.Gypsy. (1370hits, 3.07%) |
|  | CL11 | 20280 | 1.30 | LTR.Gypsy (1292hits, 2.76%) |
|  | CL12 | 19116 | 1.23 | LTR.Gypsy (9634hits, 43.7%) |
|  | CL13 | 18099 | 1.16 | LTR.Gypsy (9579hits, 44.6%) |
|  | CL14 | 17353 | 1.11 | Simple_repeat (235hits, 0.19%) |
|  | CL15 | 16356 | 1.05 | LTR.Copia (17142hits, 98.5%) |
|  | CL16 | 16305 | 1.05 | DNA.CMC.EnSpm (15646hits, 78.1%) |
|  | CL17 | 16218 | 1.04 | LTR.Gypsy (7270hits, 36.3%) |
|  | CL18 | 16074 | 1.03 | Satellite.telo (388hits, 1.25%) |
| Total | 18 | 417752 | 26.81 | - |
| Average | 1 | 23208 | 1.49% | - |
