# Supplementary material for: The repetitive DNA landscape in Avena (Poaceae): chromosome and genome evolution defined by major repeat classes in whole-genome sequence reads
Source: BMC Plant Biol. 2019 May 30;19:226. doi: 10.1186/s12870-019-1769-z (PMC6543597; doi:10.1186/s12870-019-1769-z)
Supplement: Supplementary file 16 — Table S4. The NCBI blast results of 821 clusters of four Avena species. Cluster and the longest contigs within, description, query coverage and identity are listed for each cluster. (DOCX 92 kb) [file 12870_2019_1769_MOESM16_ESM.docx]

**Table S4.** The NCBI blast results of 821 clusters of four *Avena* species.

| Cluster and the longest contig within | Contig length (bp) | Description (Highly similar sequences (megablast)) | Quary coverage | Sort by identity |
| --- | --- | --- | --- | --- |
| ***Avena sativa* L. (312; 6*x* = 42; AACCDD)** | | | | |
| CL1C25 | 2605 | Avena strigosa beta-amyrin synthase (Sad1) and cytochrome P450 CYP51H10 (Sad2) genes | 99% | 89% |
| CL2C103 | 1613 | Avena strigosa beta-amyrin synthase (Sad1) and cytochrome P450 CYP51H10 (Sad2) genes, complete cds | 92% | 85% |
| CL3C110 | 1526 | Avena strigosa beta-amyrin synthase (Sad1) and cytochrome P450 CYP51H10 (Sad2) genes, complete cds | 98% | 92% |
| CL4C316 | 4632 | Triticum aestivum chromosome 3B, genomic scaffold, cultivar Chinese Spring | 74% | 75% |
| CL4C316 | 4632 | Hordeum vulgare subsp. vulgare genes for putative iron-deficiency specific 4 protein and putative ethylene-responsive transcription factor, complete cds | 64% | 73% |
| CL5C138 | 1959 | Lolium perenne retrotransposon Lotte_7D23_1 and transposon retrotransposon Camilla_7D23_1, complete sequence; and VRN1 (Lp_7D23_1) gene, complete cds | 59% | 88% |
| CL6C32 | 14229 | Avena sativa DNA, satellite sequence | 67% | 81% |
| CL6C32 | 14229 | Avena sativa clone COS46-BamH-88 C genome-specific repeat sequence | 65% | 86% |
| CL7C56 | 8791 | Avena sativa DNA, satellite sequence | 99% | 89% |
| CL7C56 | 8791 | 1. murphyi pAM1 gene | 99% | 90% |
| CL8C7 | 1379 | Avena strigosa beta-amyrin synthase (Sad1) and cytochrome P450 CYP51H10 (Sad2) genes, complete cds | 81% | 91% |
| CL9C26 | 3572 | Avena strigosa pAs93 repetitive DNA sequence | 18% | 94% |
| CL10C187 | 5360 | Avena strigosa pAs93 repetitive DNA sequence | 18% | 94% |
| CL11C292 | 1791 | Avena strigosa beta-amyrin synthase (Sad1) and cytochrome P450 CYP51H10 (Sad2) genes, complete cds | 92% | 94% |
| CL12C85 | 1666 | Avena strigosa cytochrome P450 CYP51H11 (Cyp51H11) gene, complete cds | 73% | 82% |
| CL13C205 | 2046 | No significant similarity found | 0% | 0% |
| CL13C205 | 2046 | Triticum turgidum A genome HMW glutenin A gene locus, sequence (Somewhat similar sequences (blastn)) | 13% | 82% |
| CL14C85 | 2669 | Avena sativa isolate Pc68LrkC4 sequence containing retrotransposon and repetitive DNA linked to receptor kinase gene | 37% | 86% |
| CL15C7 | 1865 | Avena sativa clone OP45 receptor kinase gene cluster, complete sequence | 70% | 85% |
| CL16C357 | 2980 | No significant similarity found | 0% | 0% |
| CL16C357 | 2980 | Oryza sativa Indica Group cultivar Shuhui498 chromosome 5 sequence(Somewhat similar sequences (blastn)) | 4% | 81% |
| CL17C141 | 2240 | Avena sativa clone OP45 receptor kinase gene cluster, complete sequence | 60% | 87% |
| CL18C40 | 5191 | Thinopyrum intermedium repetitive DNA sequence 1 | 8% | 74% |
| CL19C4 | 2007 | Avena strigosa beta-amyrin synthase (Sad1) and cytochrome P450 CYP51H10 (Sad2) genes, complete cds | 99% | 89% |
| CL20C162 | 1937 | Avena sativa clone OP45 receptor kinase gene cluster, complete sequence | 41% | 82% |
| CL21C139 | 5813 | Avena sativa receptor-like kinase extracellular domain rlk2a13 pseudogene, complete sequence | 11% | 81% |
| CL22C199 | 1253 | Avena strigosa beta-amyrin synthase (Sad1) and cytochrome P450 CYP51H10 (Sad2) genes, complete cds | 71% | 83% |
| CL23C144 | 2609 | Triticum aestivum chromosome 3B, genomic scaffold, cultivar Chinese Spring | 3% | 87% |
| CL24C44 | 2515 | Triticum aestivum chromosome 3B, genomic scaffold, cultivar Chinese Spring | 59% | 72% |
| CL25C10 | 6357 | Avena strigosa beta-amyrin synthase (Sad1) and cytochrome P450 CYP51H10 (Sad2) genes, complete cds | 82% | 83% |
| CL26C42 | 1768 | Avena strigosa beta-amyrin synthase (Sad1) and cytochrome P450 CYP51H10 (Sad2) genes, complete cds | 22% | 94% |
| CL27C167 | 4999 | Triticum aestivum chromosome 3B, genomic scaffold, cultivar Chinese Spring | 33% | 70% |
| CL28C22 | 1488 | Avena strigosa cytochrome P450 CYP51H11 (Cyp51H11) gene, complete cds | 81% | 91% |
| CL29C72 | 2282 | Avena strigosa beta-amyrin synthase (Sad1) and cytochrome P450 CYP51H10 (Sad2) genes, complete cds | 94% | 90% |
| CL30C153 | 3859 | Avena sativa receptor-like kinase extracellular domain rlk2a13 pseudogene, complete sequence | 18% | 96% |
| CL31C15 | 3345 | Triticum monococcum subsp. monococcum cultivar DV92 Sr35 region, genomic sequence | 55% | 77% |
| CL32C20 | 4168 | Avena strigosa pAs121 repetitive DNA sequence | 2% | 97% |
| CL33C215 | 4024 | Triticum aestivum chromosome 3B, genomic scaffold, cultivar Chinese Spring | 81% | 81% |
| CL33C215 | 4024 | Lolium perenne retrotransposon Camilla_74D14_1, partial sequence; VRN1 (Lp_74D14_1) and unknown (Lp_74D14_2) genes | 90% | 77% |
| CL34C83 | 1858 | Avena strigosa beta-amyrin synthase (Sad1) and cytochrome P450 CYP51H10 (Sad2) genes, complete cds | 99% | 82% |
| CL35C26 | 2619 | Avena strigosa beta-amyrin synthase (Sad1) and cytochrome P450 CYP51H10 (Sad2) genes, complete cds | 100% | 87% |
| CL35C26 | 2619 | Avena strigosa Ty1-copia retrotransposon TAS1:GAG, AP and IN regions | 76% | 87% |
| CL36C42 | 2030 | Avena sativa clone OP45 receptor kinase gene cluster, complete sequence | 52% | 86% |
| CL37C32 | 7986 | Triticum aestivum chromosome 3B, genomic scaffold, cultivar Chinese Spring | 43% | 71% |
| CL38C10 | 4072 | Avena sativa LTR-retrotransposon OARE-1 gag-pol pseudogene for polyprotein | 100% | 98% |
| CL39C55 | 2221 | Avena strigosa DNA for dispersed repeat region, clone As22 | 33% | 98% |
| CL40C49 | 1440 | Avena strigosa beta-amyrin synthase (Sad1) and cytochrome P450 CYP51H10 (Sad2) genes, complete cds | 90% | 89% |
| CL41C64 | 5452 | Triticum aestivum chromosome 3B, genomic scaffold, cultivar Chinese Spring | 7% | 81% |
| CL42C132 | 1299 | Avena strigosa beta-amyrin synthase (Sad1) and cytochrome P450 CYP51H10 (Sad2) genes, complete cds | 67% | 92% |
| CL43C1 | 2087 | Lolium perenne partial putative non-autonomous helitron with gi pseudogene fragment, clone Lp-psGI.2 | 31% | 85% |
| CL44C75 | 2736 | Avena strigosa beta-amyrin synthase (Sad1) and cytochrome P450 CYP51H10 (Sad2) genes, complete cds | 96% | 84% |
| CL45C238 | 4557 | Oryza sativa Indica Group cultivar Shuhui498 chromosome 5 sequence | 1% | 94% |
| CL46C154 | 4238 | Triticum aestivum chromosome 3B, genomic scaffold, cultivar Chinese Spring | 33% | 71% |
| CL46C154 | 4238 | Secale vavilovii isolate VA20H.2 retrotransposon gypsy-like, partial sequence | 18% | 71% |
| CL47C32 | 7506 | Brachypodium distachyon retrotransposon Copia-like REBd3, complete sequence | 93% | 91% |
| CL48C27 | 3086 | Triticum aestivum chromosome 3B, genomic scaffold, cultivar Chinese Spring | 8% | 77% |
| CL49C115 | 3252 | Avena strigosa beta-amyrin synthase (Sad1) and cytochrome P450 CYP51H10 (Sad2) genes, complete cds | 98% | 85% |
| CL50C14 | 1515 | Avena strigosa beta-amyrin synthase (Sad1) and cytochrome P450 CYP51H10 (Sad2) genes, complete cds | 98% | 85% |
| CL51C195 | 2688 | No significant similarity found | 0% | 0% |
| CL51C195 | 2688 | Triticum aestivum chromosome 3B, genomic scaffold, cultivar Chinese Spring(Somewhat similar sequences (blastn)) | 8% | 78% |
| CL52C169 | 1197 | Avena strigosa cytochrome P450 CYP51H11 (Cyp51H11) gene, complete cds | 66% | 82% |
| CL53C17 | 4347 | No significant similarity found | 0% | 0% |
| CL53C17 | 4347 | Triticum aestivum chromosome 3B, genomic scaffold, cultivar Chinese Spring (Somewhat similar sequences (blastn)) | 99% | 70% |
| CL53C17 | 4347 | Secale cereale clone BAC 84C15 retrotransposons Gypsy-13_TA-I, TREP1573, Gypsy-13_TA-I, and TREP3345, complete sequence; and satellite pSc119 sequence | 8% | 74% |
| CL54C119 | 15991 | Triticum aestivum chromosome 3B, genomic scaffold, cultivar Chinese Spring | 99% | 70% |
| CL55C32 | 8235 | No significant similarity found | 0% | 0% |
| CL55C32 | 8235 | Festuca pratensis satellite TR4 sequence (Somewhat similar sequences (blastn)) | 24% | 75% |
| CL56C122 | 2127 | Triticum aestivum chromosome 3B, genomic scaffold, cultivar Chinese Spring | 1% | 97% |
| CL57C41 | 5746 | Triticum aestivum chromosome 3B, genomic scaffold, cultivar Chinese Spring | 1% | 88% |
| CL58C81 | 1704 | Avena vaviloviana partial dispersed repeat, clone pAvKB32 | 9% | 93% |
| CL59C127 | 3047 | Lolium multiflorum gypsy-type retrotransposon RYRE1 DNA, partial cds | 14% | 81% |
| CL60C56 | 3393 | Strongyloides stercoralis genome assembly S_stercoralis_PV0001 ,scaffold SSTP_contig0000150 | 1% | 92% |
| CL61C2 | 3497 | Triticum aestivum chromosome 3B, genomic scaffold, cultivar Chinese Spring | 13% | 90% |
| CL61C2 | 3497 | Triticum turgidum subsp. durum clone BAC 1836I21 hypothetical protein and plastid acetyl-CoA carboxylase (Acc-1) genes, complete cds | 2% | 86% |
| CL62C12 | 7856 | Hordeum vulgare Ty3/gypsy retrotransposon cereba gag-pol polyprotein gene, complete cds | 72% | 81% |
| CL63C3 | 2429 | Brachypodium distachyon clone BAC DH037O21, complete sequence | 90% | 82% |
| CL64C127 | 4001 | Triticum aestivum chromosome 3B, genomic scaffold, cultivar Chinese Spring | 31% | 84% |
| CL65C53 | 2537 | Lolium perenne partial putative non-autonomous helitron with gi pseudogene fragment, clone Lp-psGI.1 | 6% | 90% |
| CL66C86 | 4103 | Triticum aestivum chromosome 3B, genomic scaffold, cultivar Chinese Spring | 84% | 78% |
| CL67C52 | 3161 | Triticum aestivum chromosome 3B, genomic scaffold, cultivar Chinese Spring | 23% | 77% |
| CL68C39 | 1430 | Avena sativa LTR-retrotransposon OARE-1 gag-pol pseudogene for polyprotein | 100% | 98% |
| CL69C119 | 1245 | Avena strigosa pAs14 repetitive DNA sequence | 15% | 84% |
| CL70C140 | 2622 | Avena strigosa beta-amyrin synthase (Sad1) and cytochrome P450 CYP51H10 (Sad2) genes, complete cds | 78% | 83% |
| CL71C51 | 2185 | Avena sativa LTR-retrotransposon OARE-1 gag-pol pseudogene for polyprotein | 100% | 96% |
| CL72C83 | 1461 | Avena sativa clone OP45 receptor kinase gene cluster, complete sequence | 84% | 88% |
| CL73C87 | 3181 | PREDICTED: Kryptolebias marmoratus neurotrophic receptor tyrosine kinase 3 (ntrk3), transcript variant X2, mRNA | 1% | 89% |
| CL74C48 | 1342 | Avena strigosa beta-amyrin synthase (Sad1) and cytochrome P450 CYP51H10 (Sad2) genes, complete cds | 76% | 74%s |
| CL75C124 | 2995 | Triticum aestivum chromosome 3B, genomic scaffold, cultivar Chinese Spring | 93% | 70% |
| CL76C9 | 35830 | Avena sterilis chloroplast, complete genome | 100% | 99% |
| CL77C23 | 2149 | Phyllostachys edulis clone 00004 putative retrotransposon protein gene, partial cds | 3% | 86% |
| CL78C75 | 3516 | Brachypodium distachyon clone BAC DH037O21, complete sequence | 29% | 73% |
| CL79C166 | 6449 | Triticum aestivum chromosome 3B, genomic scaffold, cultivar Chinese Spring | 74% | 86% |
| CL80C136 | 2803 | Agrostis transcaspica clone AtransGAAT21_insert microsatellite AgrosSSR9 sequences | 2% | 82% |
| CL81C39 | 1446 | Triticum aestivum chromosome 3B, genomic scaffold, cultivar Chinese Spring | 3% | 100% |
| CL81C39 | 1446 | Triticum aestivum cultivar Chinese Spring clone BAC 400N24 cytokinin oxidase/dehydrogenase (CKX2.3), hypothetical protein (400.2), putative pentatricopeptide protein (400.3), and hypothetical protein (400.4) genes, complete cds | 2% | 97% |
| CL82C115 | 3992 | No significant similarity found | 0% | 0% |
| CL82C115 | 3992 | Triticum aestivum chromosome 3B, genomic scaffold, cultivar Chinese Spring (Somewhat similar sequences (blastn)) | 20% | 69% |
| CL82C115 | 3992 | PREDICTED: Aegilops tauschii subsp. tauschii DNA topoisomerase 2-like (LOC109755816), transcript variant X7, mRNA | 6% | 68% |
| CL83C5 | 1853 | No significant similarity found | 0% | 0% |
| CL83C5 | 1853 | Festuca pratensis satellite TR4 sequence | 20% | 72% |
| CL84C23 | 6486 | Brachypodium distachyon retrotransposon Gypsy CRBd1, complete sequence | 26% | 76% |
| CL85C84 | 1048 | Hordeum vulgare clone HV_Mba574-B01, complete sequence | 12% | 85% |
| CL86C219 | 1853 | Triticum aestivum chromosome 3B, genomic scaffold, cultivar Chinese Spring | 8% | 92% |
| CL86C219 | 1853 | Phyllostachys edulis clone 00049 hypothetical protein genes, complete cds | 4% | 87% |
| CL87C40 | 4866 | Triticum aestivum cultivar Chinese Spring hexose carrier, LR34, cytochrome P450, lectin receptor kinases, and cytochrome P450 genes, complete cds | 63% | 81% |
| CL88C9 | 7068 | Zea mays BAC clone CH201-151G9 from chromosome 5, complete sequence | 80% | 81% |
| CL88C9 | 7068 | Brachypodium distachyon retrotransposon Copia-like REBd3, complete sequence | 62% | 77% |
| CL89C11 | 4244 | PREDICTED: Alligator mississippiensis apoptosis associated tyrosine kinase (AATK), transcript variant X3, mRNA | 0% | 100% |
| CL90C111 | 3125 | No significant similarity found (Somewhat similar sequences (blastn)) | 0% | 0% |
| CL90C111 | 3125 | Leptomonas pyrrhocoris putative mitochondrial hypothetical protein mRNA | 1% | 94% |
| CL91C29 | 2943 | Triticum aestivum chromosome 3B, genomic scaffold, cultivar Chinese Spring | 13% | 73% |
| CL92C44 | 5624 | Avena strigosa beta-amyrin synthase (Sad1) and cytochrome P450 CYP51H10 (Sad2) genes, complete cds | 91% | 90% |
| CL93C3 | 2260 | Lolium perenne retrotransposon Lotte_7D23_1 and transposon retrotransposon Camilla_7D23_1, complete sequence; and VRN1 (Lp_7D23_1) gene, complete cds | 1% | 98% |
| CL94C12 | 2351 | No significant similarity found | 0% | 0% |
| CL94C12 | 2351 | Triticum aestivum cultivar Chinese Spring clone BAC 400N24 cytokinin oxidase/dehydrogenase (CKX2.3), hypothetical protein (400.2), putative pentatricopeptide protein (400.3), and hypothetical protein (400.4) genes, complete cds (Somewhat similar sequences (blastn)) | 1% | 91% |
| CL95C29 | 3412 | Triticum monococcum phosphatidylserine decarboxylase, ZCCT2, ZCCT1, and SNF2P genes, complete cds; nucellin pseudogene, complete sequence; putative transposase, phosphatidylinositol phosphatidylcholine transfer protein sec14 cytosolic-like protein, and phytochrome P450-like protein genes, complete cds; and unknown genes | 75% | 74% |
| CL96C84 | 6985 | Triticum turgidum subsp. durum ethylene response factor 1 (ERF1) gene, complete cds | 1% | 79% |
| CL97C14 | 9134 | Secale cereale external transcribed spacer, 18S ribosomal RNA gene, internal transcribed spacer 1, 5.8S ribosomal RNA gene, and internal transcribed spacer 2, complete sequence; and 26S ribosomal RNA gene, partial sequence | 63% | 97% |
| CL98C70 | 2005 | Avena clauda receptor-like kinase extracellular domain lrka18a4 pseudogene, partial sequence | 29% | 87% |
| CL99C55 | 5451 | No significant similarity found | 0% | 0% |
| CL99C55 | 5451 | Aegilops tauschii chromosome 1Ds prolamin gene locus, complete sequence (Somewhat similar sequences (blastn)) | 9% | 68% |
| CL100C13 | 2089 | No significant similarity found | 0% | 0% |
| CL100C13 | 2089 | PREDICTED: Peromyscus maniculatus bairdii zinc finger and BTB domain containing 4 (Zbtb4), transcript variant X4, mRNA (Somewhat similar sequences (blastn)) | 2% | 86% |
| CL101C42 | 1007 | Avena strigosa beta-amyrin synthase (Sad1) and cytochrome P450 CYP51H10 (Sad2) genes, complete cds | 100% | 94% |
| CL102C12 | 1571 | No significant similarity found | 0% | 0% |
| CL102C12 | 1571 | Avena strigosa beta-amyrin synthase (Sad1) and cytochrome P450 CYP51H10 (Sad2) genes, complete cds (Somewhat similar sequences (blastn)) | 68% | 74% |
| CL103C163 | 2619 | No significant similarity found | 0% | 0% |
| CL103C163 | 2619 | Sorghum bicolor hypothetical protein, mRNA (Somewhat similar sequences (blastn)) | 5% | 69% |
| CL104C37 | 2668 | Hordeum vulgare clone HV_Mba727-J05, complete sequence | 1% | 95% |
| CL105C58 | 1992 | No significant similarity found | 0% | 0% |
| CL105C58 | 1992 | Sporisorium scitamineum strain SscI8 genome assembly, scaffold: SPSC_scaffold_18 (Somewhat similar sequences (blastn)) | 2% | 84% |
| CL106C59 | 1951 | Avena strigosa beta-amyrin synthase (Sad1) and cytochrome P450 CYP51H10 (Sad2) genes, complete cds | 26% | 79% |
| CL107C2 | 37497 | Avena sativa voucher CAN:Saarela 775 plastid, complete genome | 99% | 99% |
| CL108C21 | 10141 | Hordeum vulgare subsp. vulgare eIF4E gene locus, complete sequence | 12% | 74% |
| CL109C91 | 2656 | Avena sativa DNA, satellite sequence | 4% | 87% |
| CL110C37 | 1747 | Helobdella robusta hypothetical protein partial mRNA | 1% | 100% |
| CL111C21 | 4541 | Hordeum vulgare subsp. vulgare Rpg4 gene, complete sequence; RGA1 (RGA1) gene, complete cds; Rpg5 gene, complete sequence; PP2C (PP2C) gene, complete cds; and ADF3 gene, complete sequence | 38% | 75% |
| CL112C37 | 4515 | Agropyron cristatum SCAR marker AcSCC5.815 genomic sequence | 17% | 43% |
| CL113C76 | 1669 | Avena strigosa beta-amyrin synthase (Sad1) and cytochrome P450 CYP51H10 (Sad2) genes, complete cds | 19% | 89% |
| CL114C25 | 1246 | Avena strigosa beta-amyrin synthase (Sad1) and cytochrome P450 CYP51H10 (Sad2) genes, complete cds | 78% | 84% |
| CL115C28 | 2195 | No significant similarity found | 0% | 0% |
| CL115C28 | 2195 | Secale cereale clone BAC 84C15 retrotransposons Gypsy-13_TA-I, TREP1573, Gypsy-13_TA-I, and TREP3345, complete sequence; and satellite pSc119 sequence (Somewhat similar sequences (blastn)) | 13% | 79% |
| CL116C72 | 2305 | Hordeum vulgare subsp. vulgare clone BAC 519K7 transposon gypsy-like LTR-retrotransposon Ashbury-1 and CACTA transposon Caspar-1, partial sequence; Tourist MITE and transposon copia-like LTR-retrotransposon HORPIA-2, complete sequence | 32% | 79% |
| CL117C30 | 6308 | Panicum virgatum clone PV_ABa107-B06, complete sequence | 51% | 78% |
| CL118C26 | 3910 | Avena sativa clone OP9 receptor kinase gene cluster, complete sequence | 4% | 82% |
| CL119C15 | 5259 | No significant similarity found | 0% | 0% |
| CL119C15 | 5259 | Festuca pratensis satellite TR4 sequence (Somewhat similar sequences (blastn)) | 97% | 69% |
| CL120C21 | 1249 | Avena strigosa beta-amyrin synthase (Sad1) and cytochrome P450 CYP51H10 (Sad2) genes, complete cds | 98% | 79% |
| CL121C91 | 2080 | Oryza minuta clone OM__Ba0145B16, complete sequence | 2% | 94% |
| CL122C22 | 1162 | Avena fatua dormancy-associated clone AFD2 putative ORF1 mRNA, 3'UTR | 32% | 94% |
| CL123C90 | 3020 | No significant similarity found | 0% | 0% |
| CL123C90 | 3020 | Oryza sativa Indica Group cultivar Shuhui498 chromosome 6 sequence (Somewhat similar sequences (blastn)) | 9% | 84% |
| CL124C55 | 3187 | No significant similarity found | 0% | 0% |
| CL124C55 | 3187 | Avena sativa clone COS204-Sau3AI-193c C genome-specific repeat sequence (Somewhat similar sequences (blastn)) | 56% | 75% |
| CL125C11 | 3269 | Avena sativa SCAR marker BG2 genomic sequence | 36% | 96% |
| CL126C47 | 3872 | Aegilops tauschii chromosome 1Ds prolamin gene locus, complete sequence | 75% | 77% |
| CL127C8 | 3855 | Avena strigosa beta-amyrin synthase (Sad1) and cytochrome P450 CYP51H10 (Sad2) genes, complete cds | 99% | 97% |
| CL128C30 | 2767 | Triticum aestivum chromosome 3B, genomic scaffold, cultivar Chinese Spring | 3% | 88% |
| CL129C21 | 2511 | Hordeum vulgare cultivar Cepada capa Rym4 and MCT-1 genes, complete cds | 34% | 79% |
| CL130C55 | 1836 | Lolium perenne partial putative non-autonomous helitron with gi pseudogene fragment, clone Lp-psGI.1 | 13% | 86% |
| CL131C35 | 3080 | Triticum aestivum chromosome 3B, genomic scaffold, cultivar Chinese Spring | 34% | 83% |
| CL132C9 | 5637 | Triticum aestivum chromosome 3B, genomic scaffold, cultivar Chinese Spring | 90% | 87% |
| CL133C12 | 2155 | Triticum aestivum clone 1716-E15 genomic sequence | 3% | 79% |
| CL134C2 | 4501 | Avena strigosa beta-amyrin synthase (Sad1) and cytochrome P450 CYP51H10 (Sad2) genes, complete cds | 99% | 96% |
| CL135C7 | 4696 | Brachypodium sylvaticum hypothetical protein (57h21.1), methyl sulfoxide reductase (57h21.2), hypothetical protein (57h21.3), oxidoreductase (57h21.4), auxin efflux carrier (57h21.6), CCR4 associated factor-like protein (57h21.7), ribosomal subunit 8E protein (57h21.8), expressed protein (57h21.9), expressed protein (57h21.10), acid phosphatase (57h21.11), polygalacturonase inhibitor (57h21.12), proteasome (57h21.13), leucine carboxyl methyl transferase (57h21.14), homeobox (57h21.15), serine threonine kinase (57h21.16), hypothetical protein (57h21.17), GTP binding protein (57h21.18), zinc carboxy peptidase (57h21.20), DNA repair protein (57h21.21), coatomer complex subunit (57h21.22), sulfate transporter (57h21.23), hypothetical protein (57h21.24), hypothetical protein (57h21.25), hypothetical protein (57h21.26), acyl-ATP thioesterase (57h21.27), hypothetical protein (57h21.28), hypothetical protein (57h21.29), superoxide dismutase (57h21.30), expressed protein (57h21.31), protein kinase (57h21.32), early nodulin protein (57h21.33), early nodulin protein (57h21.34), hypothetical protein (57h21.36), hypothetical protein (57h21.37), and expressed protein (57h21.38) genes, complete cds | 94% | 84% |
| CL136C13 | 3912 | Triticum aestivum chromosome 3B, genomic scaffold, cultivar Chinese Spring | 44% | 75% |
| CL137C7 | 2864 | Triticum aestivum chromosome 3B, genomic scaffold, cultivar Chinese Spring | 87% | 74% |
| CL138C16 | 2525 | Hordeum vulgare subsp. spontaneum voucher OUH602, partial sequence | 59% | 71% |
| CL139C35 | 3943 | Hordeum vulgare subsp. vulgare eIF4E gene locus, complete sequence | 81% | 75% |
| CL140C1 | 5130 | Triticum aestivum chromosome 3B, genomic scaffold, cultivar Chinese Spring | 95% | 82% |
| CL141C1 | 1663 | Triticum aestivum chromosome 3B, genomic scaffold, cultivar Chinese Spring | 17% | 73% |
| CL142C1 | 15763 | Avena sativa voucher CAN:Saarela 775 plastid, complete genome | 100% | 99% |
| CL143C29 | 1727 | Triticum aestivum chromosome 3B, genomic scaffold, cultivar Chinese Spring | 24% | 89% |
| CL144C1 | 3617 | PREDICTED: Hydra vulgaris uncharacterized LOC105845024 (LOC105845024), mRNA | 1% | 89% |
| CL145C49 | 1765 | Triticum aestivum chromosome 3B, genomic scaffold, cultivar Chinese Spring | 99% | 71% |
| CL146C26 | 4552 | Triticum turgidum subsp. durum Pm3 locus, genomic sequence | 55% | 75% |
| CL147C5 | 1758 | Triticum aestivum chromosome 3B-specific BAC library, contig ctg0954b | 47% | 78% |
| CL148C17 | 5197 | Triticum monococcum subsp. aegilopoides clone BAC TbBAC5, complete sequence | 9% | 72% |
| CL149C4 | 1659 | Lolium perenne retrotransposon Lotte_7D23_1 and transposon retrotransposon Camilla_7D23_1, complete sequence; and VRN1 (Lp_7D23_1) gene, complete cds | 96% | 83% |
| CL150C26 | 1258 | Avena sativa retrotransposon Cassandra, complete sequence | 99% | 89% |
| CL151C5 | 2048 | Festuca pratensis satellite TR4 sequence | 21% | 75% |
| CL152C53 | 2141 | Apteryx australis mantelli genome assembly AptMant0, scaffold scaffold3 | 1% | 90% |
| CL153C14 | 2529 | Avena sativa clone COS204-Sau3AI-194a C genome-specific repeat sequence | 97% | 96% |
| CL154C31 | 1613 | Brachypodium sylvaticum genomic construct, BACs 73P1 and 17E7 | 2% | 96% |
| CL155C34 | 4518 | Triticum aestivum chromosome 3B, genomic scaffold, cultivar Chinese Spring | 87% | 76% |
| CL156C20 | 3589 | Avena sativa clone OP9 receptor kinase gene cluster, complete sequence | 3% | 82% |
| CL157C16 | 2153 | Triticum aestivum chromosome 3B, genomic scaffold, cultivar Chinese Spring | 74% | 76% |
| CL158C13 | 1334 | Avena strigosa beta-amyrin synthase (Sad1) and cytochrome P450 CYP51H10 (Sad2) genes, complete cds | 84% | 79% |
| CL159C2 | 9532 | Avena sativa voucher CAN:Saarela 775 plastid, complete genome | 99% | 99% |
| CL160C15 | 3752 | Triticum aestivum chromosome 3B, genomic scaffold, cultivar Chinese Spring | 77% | 81% |
| CL161C16 | 5070 | Triticum aestivum chromosome 3B, genomic scaffold, cultivar Chinese Spring | 14% | 77% |
| CL162C6 | 5414 | Hordeum vulgare subsp. spontaneum MKK3 gene for mitogen-activated protein kinase kinase 3, complete cds, cultivar: OUH602 | 25% | 81% |
| CL163C40 | 994 | Avena sativa receptor-like kinase extracellular domain rlk6a2 pseudogene, complete sequence | 99% | 91% |
| CL164C11 | 4678 | Triticum aestivum chromosome 3B, genomic scaffold, cultivar Chinese Spring | 86% | 87% |
| CL165C14 | 1324 | Triticum aestivum chromosome 3B, genomic scaffold, cultivar Chinese Spring | 8% | 63% |
| CL166C37 | 1814 | PREDICTED: Sesamum indicum protein FAF-like, chloroplastic (LOC105170077), mRNA | 1% | 92% |
| CL167C6 | 3046 | Triticum aestivum chromosome 3B, genomic scaffold, cultivar Chinese Spring | 96% | 70% |
| CL168C1 | 7505 | Avena sativa voucher CAN:Saarela 775 plastid, complete genome | 100% | 99% |
| CL169C16 | 4261 | Triticum aestivum chromosome 3B, genomic scaffold, cultivar Chinese Spring | 61% | 73% |
| CL170C9 | 1627 | Hordeum vulgare subsp. vulgare clone BAC 519K7 transposon gypsy-like LTR-retrotransposon Ashbury-1 and CACTA transposon Caspar-1, partial sequence; Tourist MITE and transposon copia-like LTR-retrotransposon HORPIA-2, complete sequence; ATPase 1 pseudogene, partial sequence; ATPase 2 and hypothetical protein genes, complete cds; Stowaway MITE Damocles, Tourist MITE, Stowaway MITE Icarus and Stowaway MITE Hades-1, complete sequence; hordoindoline b-2 and hordoindoline b-1 genes, complete cds; CACTA deletion derivative, gypsy-like LTR retrotransposon BAGY-2, gypsy-like LTR-retrotransposon Sabrina-1, gypsy-like LTR-retrotransposon Lolaog, copia-like LTR-retrotransposon BARE-1-1, and copia-like LTR-retrotransposon Inga, MITE, and CACTA transposon Caspar-2, complete sequence; hordoindoline a gene, complete cds; MITEs, complete sequence; ATPase 3 and putative chalcone synthase genes, complete cds; Tourist MITE, complete sequence; putative synaptobrevin/VAMP, putative N-acetylglucosaminyltransferase, and grain softness protein genes, complete cds; transposons copia-like LTR-retrotransposon Maximus, copia-like LTR-retrotransposon BARE-2-1, copia-like LTR-retrotransposon BARE-2-2, gypsy-like LTR-retrotransposon Vagabond, copia-like LTR-retrotransposon BARE-1 2, gypsy-like LTR-retrotransposon Sabrina-2, gypsy-like LTR-retrotransposon HORGY, gypsy-like LTR-retrotransposon Haight, and copia-like LTR-retrotransposon BARE-1-3, tRNA-Arg gene, and Tourist MITE, complete sequence; hypothetical protien gene, complete cds; Stowaway MITE Hades-2, tRNA-Arg gene, MITE, transposons gypsy-like LTR-retrotransposon Ashbury-2 and copia-like LTR-retrotransposon BARE-1-4, Stowaway MITE, Stowaway MITE Thalos and Stowaway MITE Athos, complete sequence; and transposon copia-like LTR-retrotransposon BARE-2-3, partial sequence | 66% | 78% |
| CL171C13 | 2171 | Festuca pratensis satellite TR4 sequence | 23% | 77% |
| CL172C23 | 3129 | Triticum aestivum chromosome 3B, genomic scaffold, cultivar Chinese Spring | 59% | 73% |
| CL173C8 | 2030 | Oryza sativa Indica Group cultivar Shuhui498 chromosome 12 sequence | 8% | 91% |
| CL174C12 | 4397 | Triticum aestivum chromosome 3B, genomic scaffold, cultivar Chinese Spring | 58% | 80% |
| CL175C6 | 1368 | Festuca pratensis satellite TR4 sequence | 74% | 77% |
| CL176C5 | 3413 | Avena fatua clone Afat26-PI545459 5S ribosomal RNA gene, complete sequence | 98% | 100% |
| CL177C14 | 1499 | Triticum aestivum chromosome 3B, genomic scaffold, cultivar Chinese Spring | 87% | 80% |
| CL178C15 | 2897 | Triticum aestivum chromosome 3B, genomic scaffold, cultivar Chinese Spring | 55% | 78% |
| CL179C13 | 1875 | Hordeum vulgare subsp. vulgare Lks2 gene for putative short internodesfamily transcription factor, complete cds | 93% | 74% |
| CL180C10 | 1299 | Lolium perenne retrotransposon Lotte_7D23_1 and transposon retrotransposon Camilla_7D23_1, complete sequence; and VRN1 (Lp_7D23_1) gene, complete cds | 16% | 78% |
| CL181C5 | 1903 | Triticum aestivum chromosome 3B, genomic scaffold, cultivar Chinese Spring | 16% | 84% |
| CL182C17 | 1450 | Avena sativa receptor-like kinase extracellular domain rlk6a2 pseudogene, complete sequence | 77% | 76% |
| CL183C9 | 2114 | No significant similarity found | 0% | 0% |
| CL183C9 | 2114 | Triticum aestivum chromosome 3B, genomic scaffold, cultivar Chinese Spring (Somewhat similar sequences (blastn)) | 3% | 89% |
| CL184C18 | 2434 | Lolium perenne LINE retrotransposon DNA, endonuclease region of PRLN3 | 5% | 76% |
| CL185C13 | 1074 | No significant similarity found | 0% | 0% |
| CL185C13 | 1074 | Triticum aestivum chromosome 3B, genomic scaffold, cultivar Chinese Spring (Somewhat similar sequences (blastn)) | 27% | 72% |
| CL186C2 | 2255 | Oryza minuta clone OM__Ba0177M23, complete sequence | 74% | 87% |
| CL187C6 | 1283 | No significant similarity found | 0% | 0% |
| CL187C6 | 1283 | 1. sativa rDNA spacer (Somewhat similar sequences (blastn)) | 87% | 64% |
| CL188C11 | 1185 | Triticum aestivum chromosome 3B, genomic scaffold, cultivar Chinese Spring | 67% | 78% |
| CL189C12 | 844 | Hordeum vulgare clone HV_Mba329-C16, complete sequence | 5% | 96% |
| CL190C12 | 2290 | PREDICTED: Aegilops tauschii subsp. tauschii pseudouridylate synthase 7 homolog (LOC109740888), transcript variant X7, mRNA | 11% | 75% |
| CL191C4 | 2133 | Triticum aestivum clone pTa-885 FISH-positive repetitive sequence | 86% | 72% |
| CL192C15 | 1085 | Triticum aestivum chromosome 3B, genomic scaffold, cultivar Chinese Spring | 30% | 78% |
| CL193C6 | 2302 | Triticum aestivum chromosome 3B, genomic scaffold, cultivar Chinese Spring | 11% | 76% |
| CL194C8 | 3109 | Aegilops tauschii chromosome 1Ds prolamin gene locus, complete sequence | 33% | 85% |
| CL195C16 | 1203 | Phakopsora pachyrhizi clone JGIAFNA-1989A17, complete sequence | 3% | 89% |
| CL196C4 | 1267 | PREDICTED: Halyomorpha halys insulin-like growth factor-binding protein complex acid labile subunit (LOC106687647), transcript variant X2, mRNA | 2% | 96% |
| CL197C2 | 972 | Aeromonas media WS, complete genome | 2% | 96% |
| CL198C2 | 1947 | Triticum aestivum chromosome 3B, genomic scaffold, cultivar Chinese Spring | 32% | 78% |
| CL199C11 | 1635 | PREDICTED: Aegilops tauschii subsp. tauschii E3 ubiquitin-protein ligase SINA-like 4 (LOC109769695), mRNA | 4% | 80% |
| CL200C5 | 1274 | PREDICTED: Brachypodium distachyon uncharacterized LOC104581508 (LOC104581508), transcript variant X3, ncRNA | 4% | 92% |
| CL201C5 | 3487 | Triticum aestivum chromosome 3B, genomic scaffold, cultivar Chinese Spring | 55% | 73% |
| CL202C7 | 2726 | Solanum lycopersicum chromosome ch01, complete genome | 1% | 97% |
| CL203C3 | 763 | Oryza sativa Indica Group cultivar Shuhui498 chromosome 12 sequence | 8% | 91% |
| CL204C9 | 1722 | PREDICTED: Canis lupus familiaris gap junction protein, alpha 4, 37kDa (GJA4), transcript variant X2, mRNA | 1% | 100% |
| CL205C13 | 1021 | Triticum aestivum chromosome 3B, genomic scaffold, cultivar Chinese Spring | 2% | 97% |
| CL206C1 | 1497 | Triticum aestivum chromosome 3B, genomic scaffold, cultivar Chinese Spring | 75% | 86% |
| CL207C5 | 3482 | Triticum aestivum chromosome 3B, genomic scaffold, cultivar Chinese Spring | 57% | 80% |
| CL208C5 | 2746 | Hordeum vulgare clone HV_Mba152-O10, complete sequence | 100% | 93% |
| CL209C2 | 2866 | MACACA MULATTA BAC clone CH250-168M9 from chromosome unknown, complete sequence | 14% | 86% |
| CL210C6 | 3669 | Triticum aestivum chromosome 3B, genomic scaffold, cultivar Chinese Spring | 84% | 76% |
| CL211C2 | 567 | Triticum aestivum chromosome 3B, genomic scaffold, cultivar Chinese Spring | 38% | 84% |
| CL212C8 | 1250 | Triticum aestivum chromosome 3B, genomic scaffold, cultivar Chinese Spring | 9% | 82% |
| CL213C3 | 1141 | Avena strigosa beta-amyrin synthase (Sad1) and cytochrome P450 CYP51H10 (Sad2) genes, complete cds | 9% | 82% |
| CL214C3 | 1454 | Baudoinia panamericana UAMH 10762 hypothetical protein mRNA | 2% | 97% |
| ***Avena brevis* Roth (289; 2*x* = 14; AA)** | | | | |
| CL1C543 | 3470bp | Avena strigosa beta-amyrin synthase (Sad1) and cytochrome P450 CYP51H10 (Sad2) genes, complete cds | 39% | 86% |
| CL2C173 | 2153 | Avena strigosa beta-amyrin synthase (Sad1) and cytochrome P450 CYP51H10 (Sad2) genes, complete cds | 40% | 78% |
| CL3C266 | 1874 | Avena strigosa beta-amyrin synthase (Sad1) and cytochrome P450 CYP51H10 (Sad2) genes, complete cds | 71% | 86% |
| CL4C136 | 1542 | Avena strigosa beta-amyrin synthase (Sad1) and cytochrome P450 CYP51H10 (Sad2) genes, complete cds | 97% | 92% |
| CL5C253 | 1961 | Avena sativa clone OP45 receptor kinase gene cluster, complete sequence | 35% | 88% |
| CL6C181 | 4928 | Triticum aestivum chromosome 3B, genomic scaffold, cultivar Chinese Spring | 60% | 73% |
| CL7C261 | 1709 | Avena strigosa beta-amyrin synthase (Sad1) and cytochrome P450 CYP51H10 (Sad2) genes, complete cds | 47% | 88% |
| CL8C394 | 17092 | Avena sativa clone COS46-BamH-88 C genome-specific repeat sequence | 86% | 90% |
| CL9C420 | 5236 | Triticum aestivum chromosome 3B, genomic scaffold, cultivar Chinese Spring | 66% | 75% |
| CL10C94 | 16547 | Avena sativa clone COS46-BamH-88 C genome-specific repeat sequence | 65% | 85% |
| CL11C246 | 2613 | Avena sativa clone OP45 receptor kinase gene cluster, complete sequence | 59% | 92% |
| CL12C171 | 4172 | Triticum aestivum chromosome 3B, genomic scaffold, cultivar Chinese Spring | 1% | 91% |
| CL13C98 | 1664 | Lolium multiflorum gene for cold responsive protein, complete cds | 65% | 79% |
| CL14C276 | 6308 | Avena sativa receptor-like kinase extracellular domain rlk2a13 pseudogene, complete sequence | 10% | 81% |
| CL15C119 | 8714 | Avena sativa DNA, satellite sequence | 37% | 81% |
| CL16C69 | 2914 | Avena sativa isolate Pc68LrkC4 sequence containing retrotransposon and repetitive DNA linked to receptor kinase gene | 34% | 86% |
| CL17C112 | 1847 | Avena sativa clone OP45 receptor kinase gene cluster, complete sequence | 73% | 85% |
| CL18C282 | 3718 | Triticum aestivum chromosome 3B, genomic scaffold, cultivar Chinese Spring | 2% | 83% |
| CL19C781 | 2338 | No significant similarity found | 0% | 0% |
| CL19C781 | 2338 | Triticum aestivum chromosome 3B, genomic scaffold, cultivar Chinese Spring (Somewhat similar sequences (blastn)) | 20% | 86% |
| CL20C18 | 2257 | Avena strigosa beta-amyrin synthase (Sad1) and cytochrome P450 CYP51H10 (Sad2) genes, complete cds | 99% | 97% |
| CL21C328 | 5363 | Triticum aestivum chromosome 3B, genomic scaffold, cultivar Chinese Spring | 48% | 65% |
| CL22C143 | 2269 | Avena sativa clone OP45 receptor kinase gene cluster, complete sequence | 53% | 91% |
| CL23C122 | 1286 | Avena strigosa beta-amyrin synthase (Sad1) and cytochrome P450 CYP51H10 (Sad2) genes, complete cds | 99% | 90% |
| CL24C237 | 2448 | Avena strigosa pAs14 repetitive DNA sequence | 21% | 79% |
| CL25C15 | 5803 | Avena strigosa beta-amyrin synthase (Sad1) and cytochrome P450 CYP51H10 (Sad2) genes, complete cds | 83% | 83% |
| CL26C63 | 2502 | Lolium perenne retrotransposon Lotte_7D23_1 and transposon retrotransposon Camilla_7D23_1, complete sequence; and VRN1 (Lp_7D23_1) gene, complete cds | 87% | 82% |
| CL27C38 | 6171 | Avena strigosa pAs111 repetitive DNA sequence | 6% | 88% |
| CL28C187 | 6354 | Triticum aestivum chromosome 3B, genomic scaffold, cultivar Chinese Spring | 64% | 81% |
| CL29C142 | 1655 | Avena strigosa cytochrome P450 CYP51H11 (Cyp51H11) gene, complete cds | 76% | 87% |
| CL30C188 | 2338 | Avena strigosa beta-amyrin synthase (Sad1) and cytochrome P450 CYP51H10 (Sad2) genes, complete cds | 85% | 84% |
| CL31C68 | 1801 | Avena strigosa beta-amyrin synthase (Sad1) and cytochrome P450 CYP51H10 (Sad2) genes, complete cds | 83% | 76% |
| CL32C126 | 3998 | Hordeum vulgare clone HV_Mba113-M07, complete sequence | 14% | 73% |
| CL33C90 | 3331 | Triticum aestivum chromosome 3B, genomic scaffold, cultivar Chinese Spring | 16% | 76% |
| CL34C108 | 1884 | Avena strigosa beta-amyrin synthase (Sad1) and cytochrome P450 CYP51H10 (Sad2) genes, complete cds | 32% | 88% |
| CL35C68 | 1970 | Avena strigosa beta-amyrin synthase (Sad1) and cytochrome P450 CYP51H10 (Sad2) genes, complete cds | 93% | 82% |
| CL36C19 | 2636 | Avena strigosa beta-amyrin synthase (Sad1) and cytochrome P450 CYP51H10 (Sad2) genes, complete cds | 100% | 87% |
| CL37C103 | 3477 | Lolium perenne retrotransposon Lotte_7D23_1 and transposon retrotransposon Camilla_7D23_1, complete sequence; and VRN1 (Lp_7D23_1) gene, complete cds | 12% | 80% |
| CL38C52 | 2314 | Avena strigosa DNA for dispersed repeat region, clone As22 | 31% | 98% |
| CL39C28 | 4026 | Avena sativa LTR-retrotransposon OARE-1 gag-pol pseudogene for polyprotein | 99% | 98% |
| CL40C134 | 1771 | Avena strigosa beta-amyrin synthase (Sad1) and cytochrome P450 CYP51H10 (Sad2) genes, complete cds | 62% | 95% |
| CL41C104 | 1284 | Avena strigosa beta-amyrin synthase (Sad1) and cytochrome P450 CYP51H10 (Sad2) genes, complete cds | 82% | 89% |
| CL42C45 | 5052 | Triticum aestivum chromosome 3B, genomic scaffold, cultivar Chinese Spring | 28% | 71% |
| CL43C245 | 3711 | Avena sativa receptor-like kinase extracellular domain rlk2a13 pseudogene, complete sequence | 3% | 97% |
| CL44C130 | 6829 | Triticum aestivum chromosome 3B, genomic scaffold, cultivar Chinese Spring | 50% | 71% |
| CL45C16 | 2900 | Avena strigosa beta-amyrin synthase (Sad1) and cytochrome P450 CYP51H10 (Sad2) genes, complete cds | 99% | 94% |
| CL46C74 | 2025 | Lolium perenne partial putative non-autonomous helitron with gi pseudogene fragment, clone Lp-psGI.2 | 9% | 85% |
| CL47C372 | 12417 | Avena sativa DNA, satellite sequence | 96% | 84% |
| CL48C107 | 2498 | Avena strigosa beta-amyrin synthase (Sad1) and cytochrome P450 CYP51H10 (Sad2) genes, complete cds | 99% | 74% |
| CL49C1 | 10046 | No significant similarity found | 0% | 0% |
| CL49C1 | 10046 | Festuca pratensis satellite TR4 sequence (Somewhat similar sequences (blastn)) | 21% | 75% |
| CL50C34 | 1864 | Avena strigosa beta-amyrin synthase (Sad1) and cytochrome P450 CYP51H10 (Sad2) genes, complete cds | 99% | 95% |
| CL51C70 | 7125 | Brachypodium distachyon retrotransposon Copia-like REBd3, complete sequence | 95% | 91% |
| CL52C36 | 3088 | Triticum aestivum chromosome 3B, genomic scaffold, cultivar Chinese Spring | 29% | 71% |
| CL53C8 | 38096 | Avena sterilis chloroplast, complete genome | 99% | 100% |
| CL54C119 | 4169 | Oryza sativa Indica Group cultivar Shuhui498 chromosome 4 sequence | 27% | 71% |
| CL55C178 | 3365 | Triticum aestivum chromosome 3B, genomic scaffold, cultivar Chinese Spring | 4% | 90% |
| CL56C32 | 4127 | No significant similarity found | 0% | 0% |
| CL56C32 | 4127 | Triticum aestivum chromosome 3B, genomic scaffold, cultivar Chinese Spring (Somewhat similar sequences (blastn)) | 98% | 67% |
| CL57C168 | 5966 | Triticum aestivum chromosome 3B, genomic scaffold, cultivar Chinese Spring | 26% | 76% |
| CL58C111 | 4103 | Triticum aestivum chromosome 3B, genomic scaffold, cultivar Chinese Spring | 84% | 78% |
| CL59C82 | 2472 | Hordeum vulgare subsp. vulgare BAC 259I16, complete sequence | 72% | 68% |
| CL60C79 | 5334 | Triticum aestivum chromosome 3B, genomic scaffold, cultivar Chinese Spring | 8% | 78% |
| CL61C21 | 8344 | Triticum aestivum chromosome 3B specific BAC library, BAC clone TaaCsp3BFhA_0147D05 | 57% | 81% |
| CL62C33 | 1467 | Avena sativa LTR-retrotransposon OARE-1 gag-pol pseudogene for polyprotein | 99% | 95% |
| CL63C301 | 4331 | No significant similarity found | 0% | 0% |
| CL63C301 | 4331 | Triticum aestivum chromosome 3B, genomic scaffold, cultivar Chinese Spring (Somewhat similar sequences (blastn)) | 22% | 92% |
| CL64C41 | 1973 | Triticum aestivum chromosome 3B, genomic scaffold, cultivar Chinese Spring | 2% | 95% |
| CL65C51 | 3482 | Triticum aestivum chromosome 3B, genomic scaffold, cultivar Chinese Spring | 6% | 78% |
| CL66C38 | 2875 | Triticum aestivum chromosome 3B, genomic scaffold, cultivar Chinese Spring | 1% | 95% |
| CL67C34 | 2255 | Brachypodium distachyon clone BAC DH037O21, complete sequence | 88% | 83% |
| CL68C36 | 2189 | Avena sativa LTR-retrotransposon OARE-1 gag-pol pseudogene for polyprotein | 99% | 98% |
| CL69C93 | 4962 | No significant similarity found | 0% | 0% |
| CL69C93 | 4962 | Triticum aestivum chromosome 3B, genomic scaffold, cultivar Chinese Spring (Somewhat similar sequences (blastn)) | 97% | 68% |
| CL70C67 | 2757 | No significant similarity found | 0% | 0% |
| CL70C67 | 2757 | Oceanisphaera sp. AMac2203, complete genome | 1% | 89% |
| CL71C33 | 2937 | No significant similarity found | 0% | 0% |
| CL71C33 | 2937 | Agrostis transcaspica clone AtransGAAT21_insert microsatellite AgrosSSR9 sequences (Somewhat similar sequences (blastn)) | 5% | 75% |
| CL72C195 | 3271 | Avena clauda receptor-like kinase extracellular domain lrka18a4 pseudogene, partial sequence | 26% | 91% |
| CL73C138 | 2515 | A.sativa GLAV 3 gene for 11S globulin | 15% | 89% |
| CL74C46 | 3558 | Triticum aestivum chromosome 3B, genomic scaffold, cultivar Chinese Spring | 22% | 79% |
| CL75C117 | 1157 | Hordeum vulgare clone HV_Mba574-B01, complete sequence | 11% | 85% |
| CL76C68 | 2077 | No significant similarity found | 0% | 0% |
| CL76C68 | 2077 | Mus musculus chromosome 13, clone RP23-104G24, complete sequence (Somewhat similar sequences (blastn)) | 1% | 100% |
| CL77C122 | 5422 | Triticum aestivum chromosome 3B, genomic scaffold, cultivar Chinese Spring | 78% | 86% |
| CL78C94 | 8056 | Brachypodium distachyon retrotransposon BD-Tekay, partial sequence | 39% | 75% |
| CL79C300 | 2566 | Phyllostachys heterocycla genomic DNA, BAC clone: PH01B031C15, complete sequence | 2% | 96% |
| CL80C4 | 4864 | Secale cereale external transcribed spacer, 18S ribosomal RNA gene, internal transcribed spacer 1, 5.8S ribosomal RNA gene, and internal transcribed spacer 2, complete sequence; and 26S ribosomal RNA gene, partial sequence | 63% | 96% |
| CL81C103 | 4070 | No significant similarity found | 0% | 0% |
| CL81C103 | 4070 | Allium sativum microsatellite Asa31 sequence (Somewhat similar sequences (blastn)) | 8% | 69% |
| CL82C114 | 5221 | No significant similarity found | 0% | 0% |
| CL82C114 | 5221 | Eimeria mitis hypothetical protein partial mRNA (Somewhat similar sequences (blastn)) | 3% | 72% |
| CL83C21 | 1928 | No significant similarity found | 0% | 0% |
| CL83C21 | 1928 | Avena strigosa beta-amyrin synthase (Sad1) and cytochrome P450 CYP51H10 (Sad2) genes, complete cds | 77% | 69% |
| CL84C45 | 6606 | Zea mays BAC clone CH201-151G9 from chromosome 5, complete sequence | 92% | 81% |
| CL85C84 | 1608 | Avena strigosa pAs93 repetitive DNA sequence | 27% | 83% |
| CL86C16 | 4374 | No significant similarity found | 0% | 0% |
| CL86C16 | 4374 | Eimeria mitis hypothetical protein, conserved partial mRNA (Somewhat similar sequences (blastn)) | 1% | 80% |
| CL87C39 | 3077 | Triticum aestivum chromosome 3B, genomic scaffold, cultivar Chinese Spring | 14% | 75% |
| CL88C47 | 4612 | Aegilops tauschii chromosome 1Ds prolamin gene locus, complete sequence | 67% | 81% |
| CL89C12 | 3098 | No significant similarity found | 0% | 0% |
| CL89C12 | 3098 | Hordeum vulgare subsp. spontaneum voucher OUH602, partial sequence | 1% | 85% |
| CL90C22 | 6537 | Avena strigosa beta-amyrin synthase (Sad1) and cytochrome P450 CYP51H10 (Sad2) genes, complete cds | 95% | 94% |
| CL91C6 | 5326 | No significant similarity found | 0% | 0% |
| CL91C6 | 5326 | PREDICTED: Aegilops tauschii subsp. tauschii uncharacterized LOC109763087 (LOC109763087), mRNA (Somewhat similar sequences (blastn)) | 8% | 68% |
| CL92C18 | 5779 | Triticum aestivum chromosome 3B, genomic scaffold, cultivar Chinese Spring | 60% | 75% |
| CL93C5 | 3399 | No significant similarity found | 0% | 0% |
| CL93C5 | 3399 | Festuca pratensis satellite TR4 sequence (Somewhat similar sequences (blastn)) | 22% | 72% |
| CL94C24 | 5584 | Avena sativa REMAP marker D004-CAC7T genomic sequence | 3% | 92% |
| CL95C43 | 4498 | Triticum aestivum chromosome 3B, genomic scaffold, cultivar Chinese Spring | 17% | 71% |
| CL96C286 | 1994 | No significant similarity found | 0% | 0% |
| CL96C286 | 1994 | Ovis canadensis canadensis isolate 43U chromosome 1 sequence (Somewhat similar sequences (blastn)) | 2% | 83% |
| CL97C62 | 1101 | Avena strigosa beta-amyrin synthase (Sad1) and cytochrome P450 CYP51H10 (Sad2) genes, complete cds | 90% | 87% |
| CL98C12 | 1656 | No significant similarity found | 0% | 0% |
| CL98C12 | 1656 | Avena strigosa beta-amyrin synthase (Sad1) and cytochrome P450 CYP51H10 (Sad2) genes, complete cds (Somewhat similar sequences (blastn)) | 69% | 71% |
| CL99C152 | 4233 | Arabis alpina genome assembly, chromosome: chr4 | 0% | 94% |
| CL100C7 | 2627 | Triticum aestivum chromosome 3B, genomic scaffold, cultivar Chinese Spring | 38% | 77% |
| CL101C3 | 3100 | A.sativa rDNA spacer | 92% | 95% |
| CL102C89 | 4287 | Avena sativa clone OP9 receptor kinase gene cluster, complete sequence | 2% | 83% |
| CL103C124 | 2394 | Hordeum vulgare clone HV_Mba727-J05, complete sequence | 1% | 95% |
| CL104C72 | 2271 | Triticum aestivum chromosome 3B, genomic scaffold, cultivar Chinese Spring | 1% | 97% |
| CL105C17 | 3917 | No significant similarity found | 0% | 0% |
| CL105C17 | 3917 | Festuca pratensis satellite TR4 sequence (Somewhat similar sequences (blastn)) | 92% | 68% |
| CL106C25 | 1745 | No significant similarity found | 0% | 0% |
| CL106C25 | 1745 | Avena strigosa pAs93 repetitive DNA sequence (Somewhat similar sequences (blastn)) | 19% | 72% |
| CL107C56 | 1593 | Avena strigosa beta-amyrin synthase (Sad1) and cytochrome P450 CYP51H10 (Sad2) genes, complete cds | 84% | 81% |
| CL108C22 | 5005 | Hordeum vulgare cultivar Cepada capa Rym4 and MCT-1 genes, complete cds | 15% | 79% |
| CL109C80 | 4787 | Triticum aestivum chromosome 3B, genomic scaffold, cultivar Chinese Spring | 62% | 79% |
| CL110C27 | 7496 | Hordeum vulgare subsp. vulgare eIF4E gene locus, complete sequence | 17% | 74% |
| CL111C43 | 2367 | No significant similarity found | 0% | 0% |
| CL111C43 | 2367 | Triticum aestivum chromosome 3B, genomic scaffold, cultivar Chinese Spring (Somewhat similar sequences (blastn)) | 99% | 67% |
| CL112C1 | 23017 | Avena sterilis chloroplast, complete genome | 99% | 99% |
| CL113C107 | 3592 | Aegilops tauschii chromosome 1Ds prolamin gene locus, complete sequence | 63% | 75% |
| CL114C69 | 2753 | No significant similarity found | 0% | 0% |
| CL114C69 | 2753 | Homo sapiens 3 BAC RP11-488M12 (Roswell Park Cancer Institute Human BAC Library) complete sequence (Somewhat similar sequences (blastn)) | 1% | 92% |
| CL115C46 | 6686 | No significant similarity found | 0% | 0% |
| CL115C46 | 6686 | PREDICTED: Aegilops tauschii subsp. tauschii uncharacterized LOC109736966 (LOC109736966), mRNA (Somewhat similar sequences (blastn)) | 11% | 68% |
| CL116C7 | 3163 | No significant similarity found | 0% | 0% |
| CL116C7 | 3163 | Cyperus rotundus clone Cyr_42 SSR marker genomic sequence (Somewhat similar sequences (blastn)) | 8% | 69% |
| CL117C44 | 5191 | Panicum virgatum clone PV_ABa006-H10, complete sequence | 27% | 73% |
| CL118C22 | 1499 | Avena strigosa beta-amyrin synthase (Sad1) and cytochrome P450 CYP51H10 (Sad2) genes, complete cds | 67% | 79% |
| CL119C165 | 1878 | No significant similarity found | 0% | 0% |
| CL119C165 | 1878 | Avena strigosa beta-amyrin synthase (Sad1) and cytochrome P450 CYP51H10 (Sad2) genes, complete cds (Somewhat similar sequences (blastn)) | 26% | 68% |
| CL120C3 | 18071 | Avena sterilis chloroplast, complete genome | 100% | 99% |
| CL121C1 | 17190 | Avena sativa voucher CAN:Saarela 775 plastid, complete genome | 99% | 99% |
| CL122C21 | 2489 | No significant similarity found | 0% | 0% |
| CL122C21 | 2489 | Triticum aestivum chromosome 3B, genomic scaffold, cultivar Chinese Spring (Somewhat similar sequences (blastn)) | 24% | 67% |
| CL123C11 | 1124 | Avena fatua dormancy-associated clone AFD2 putative ORF1 mRNA, 3'UTR | 33% | 90% |
| CL124C41 | 3257 | Triticum aestivum chromosome 3B, genomic scaffold, cultivar Chinese Spring | 0% | 100% |
| CL125C51 | 2966 | Lolium perenne CACTA-family transposon sequence (Somewhat similar sequences (blastn)) | 50% | 76% |
| CL126C28 | 3318 | Avena sativa SCAR marker BG2 genomic sequence | 35% | 96% |
| CL127C3 | 6050 | Triticum aestivum chromosome 3B, genomic scaffold, cultivar Chinese Spring | 88% | 87% |
| CL128C3 | 3137 | Avena strigosa beta-amyrin synthase (Sad1) and cytochrome P450 CYP51H10 (Sad2) genes, complete cds | 91% | 86% |
| CL129C12 | 5205 | Triticum aestivum chromosome 3B, genomic scaffold, cultivar Chinese Spring | 0% | 100% |
| CL130C1 | 2049 | Hordeum vulgare subsp. vulgare cDNA clone: FLbaf52b15, mRNA sequence (Somewhat similar sequences (blastn)) | 100% | 99% |
| CL131C24 | 1650 | No significant similarity found | 0% | 0% |
| CL131C24 | 1650 | Triticum aestivum chromosome 3B, genomic scaffold, cultivar Chinese Spring (Somewhat similar sequences (blastn)) | 36% | 68% |
| CL132C6 | 2724 | Avena strigosa beta-amyrin synthase (Sad1) and cytochrome P450 CYP51H10 (Sad2) genes, complete cds | 92% | 96% |
| CL133C33 | 3844 | Oryza sativa Japonica Group DNA, chromosome 2, cultivar: Nipponbare, complete sequence | 28% | 71% |
| CL134C1 | 3408 | No significant similarity found | 0% | 0% |
| CL134C1 | 3408 | Triticum aestivum chromosome 3B, genomic scaffold, cultivar Chinese Spring (Somewhat similar sequences (blastn)) | 29% | 78% |
| CL135C4 | 12287 | Avena sterilis chloroplast, complete genome | 100% | 100% |
| CL136C25 | 3397 | No significant similarity found | 0% | 0% |
| CL136C25 | 3397 | Triticum aestivum chromosome 3B, genomic scaffold, cultivar Chinese Spring (Somewhat similar sequences (blastn)) | 90% | 68% |
| CL137C16 | 4619 | Brachypodium sylvaticum hypothetical protein (57h21.1), methyl sulfoxide reductase (57h21.2), hypothetical protein (57h21.3), oxidoreductase (57h21.4), auxin efflux carrier (57h21.6), CCR4 associated factor-like protein (57h21.7), ribosomal subunit 8E protein (57h21.8), expressed protein (57h21.9), expressed protein (57h21.10), acid phosphatase (57h21.11), polygalacturonase inhibitor (57h21.12), proteasome (57h21.13), leucine carboxyl methyl transferase (57h21.14), homeobox (57h21.15), serine threonine kinase (57h21.16), hypothetical protein (57h21.17), GTP binding protein (57h21.18), zinc carboxy peptidase (57h21.20), DNA repair protein (57h21.21), coatomer complex subunit (57h21.22), sulfate transporter (57h21.23), hypothetical protein (57h21.24), hypothetical protein (57h21.25), hypothetical protein (57h21.26), acyl-ATP thioesterase (57h21.27), hypothetical protein (57h21.28), hypothetical protein (57h21.29), superoxide dismutase (57h21.30), expressed protein (57h21.31), protein kinase (57h21.32), early nodulin protein (57h21.33), early nodulin protein (57h21.34), hypothetical protein (57h21.36), hypothetical protein (57h21.37), and expressed protein (57h21.38) genes, complete cds | 96% | 84% |
| CL138C36 | 1742 | No significant similarity found | 0% | 0% |
| CL138C36 | 1742 | PREDICTED: Corvus cornix cornix protein tyrosine phosphatase, receptor type D (PTPRD), transcript variant X40, mRNA (Somewhat similar sequences (blastn)) | 3% | 81% |
| CL139C17 | 4629 | Hordeum vulgare subsp. vulgare eIF4E gene locus, complete sequence | 79% | 75% |
| CL140C28 | 4852 | Triticum aestivum chromosome 3B, genomic scaffold, cultivar Chinese Spring | 94% | 83% |
| CL141C15 | 4817 | No significant similarity found | 0% | 0% |
| CL141C15 | 4817 | Triticum aestivum chromosome 3B, genomic scaffold, cultivar Chinese Spring (Somewhat similar sequences (blastn)) | 35% | 70% |
| CL142C48 | 2952 | Triticum aestivum chromosome 3B, genomic scaffold, cultivar Chinese Spring | 6% | 89% |
| CL143C1 | 4627 | Triticum turgidum subsp. durum Pm3 locus, genomic sequence | 33% | 75% |
| CL144C26 | 1534 | Triticum aestivum chromosome 3B, genomic scaffold, cultivar Chinese Spring | 75% | 78% |
| CL145C61 | 2902 | No significant similarity found | 0% | 0% |
| CL145C61 | 2902 | A.murphyi pAM1 gene (Somewhat similar sequences (blastn)) | 36% | 80% |
| CL146C44 | 1870 | No significant similarity found | 0% | 0% |
| CL146C44 | 1870 | Panicum virgatum clone PV_ABa006-H10, complete sequence (Somewhat similar sequences (blastn)) | 5% | 71% |
| CL147C28 | 1188 | Triticum aestivum chromosome 3B, genomic scaffold, cultivar Chinese Spring | 12% | 85% |
| CL148C17 | 4444 | No significant similarity found | 0% | 0% |
| CL148C17 | 4444 | Festuca pratensis satellite TR4 sequence (Somewhat similar sequences (blastn)) | 88% | 67% |
| CL149C33 | 2538 | Avena sativa cultivar Aslak REMAP marker A002-AC9G genomic sequence | 23% | 96% |
| CL150C16 | 3458 | Avena sativa clone COS204-Sau3AI-194a C genome-specific repeat sequence | 99% | 96% |
| CL151C17 | 1835 | No significant similarity found | 0% | 0% |
| CL151C17 | 1835 | Triticum aestivum chromosome 3B, genomic scaffold, cultivar Chinese Spring (Somewhat similar sequences (blastn)) | 20% | 70% |
| CL152C3 | 5606 | Triticum aestivum chromosome 3B, genomic scaffold, cultivar Chinese Spring | 2% | 83% |
| CL153C18 | 4491 | No significant similarity found | 0% | 0% |
| CL153C18 | 4491 | Avena sativa clone COS204-Sau3AI-193c C genome-specific repeat sequence (Somewhat similar sequences (blastn)) | 64% | 76% |
| CL154C6 | 1303 | No significant similarity found | 0% | 0% |
| CL154C6 | 1303 | Zebrafish DNA sequence from clone CH73-94B15 in linkage group 5, complete sequence (Somewhat similar sequences (blastn)) | 38% | 65% |
| CL155C5 | 2973 | Triticum aestivum chromosome 3B, genomic scaffold, cultivar Chinese Spring | 67% | 74% |
| CL156C18 | 3031 | No significant similarity found | 0% | 0% |
| CL156C18 | 3031 | Triticum aestivum chromosome 3B, genomic scaffold, cultivar Chinese Spring (Somewhat similar sequences (blastn)) | 100% | 68% |
| CL157C1 | 1011 | PREDICTED: Brachypodium distachyon uncharacterized LOC100821162 (LOC100821162), mRNA | 100% | 98% |
| CL158C1 | 1981 | Hordeum vulgare clone HV_Mba329-C16, complete sequence | 56% | 78% |
| CL159C21 | 1711 | Avena sativa receptor-like kinase extracellular domain rlk6a2 pseudogene, complete sequence | 90% | 86% |
| CL160C2 | 1499 | Avena sativa retrotransposon Cassandra, complete sequence | 80% | 92% |
| CL161C9 | 3979 | Triticum aestivum chromosome 3B, genomic scaffold, cultivar Chinese Spring | 71% | 80% |
| CL162C9 | 4999 | Triticum aestivum cv. Arina SNP, chromosome 3B, clone Taes_arina_ctg_71666 | 15% | 85% |
| CL163C39 | 2352 | Arabis alpina genome assembly, chromosome: chr5 | 1% | 92% |
| CL164C7 | 4778 | Triticum aestivum cultivar Chinese Spring clone BAC 351D1 chromosome 4A DELLA protein (Rht-A) gene, complete cds, complete sequence | 56% | 85% |
| CL165C7 | 1277 | Lolium perenne clone LpSSR071 SSR marker sequence | 32% | 79% |
| CL166C12 | 1783 | No significant similarity found | 0% | 0% |
| CL166C12 | 1783 | H.pratense satellite DNA (ID pPRA1_3) (Somewhat similar sequences (blastn)) | 23% | 80% |
| CL167C15 | 3123 | No significant similarity found | 0% | 0% |
| CL167C15 | 3123 | Triticum aestivum cultivar Chinese Spring clone BAC 36I14, complete sequence (Somewhat similar sequences (blastn)) | 2% | 77% |
| CL168C11 | 4693 | Hordeum vulgare subsp. vulgare cultivar Haruna Nijo voucher J247, partial sequence | 30% | 74% |
| CL169C12 | 1650 | Saitoella complicata NRRL Y-17804 cysteine proteinase partial mRNA | 1% | 94% |
| CL170C13 | 3088 | Hordeum vulgare clone HV_Mba112-L24, complete sequence | 74% | 75% |
| CL171C15 | 1671 | PREDICTED: Aegilops tauschii subsp. tauschii uncharacterized LOC109767923 (LOC109767923), partial mRNA | 2% | 97% |
| CL172C17 | 1400 | No significant similarity found | 0% | 0% |
| CL172C17 | 1400 | Oryza sativa Indica Group cultivar Shuhui498 chromosome 10 sequence (Somewhat similar sequences (blastn)) | 3% | 86% |
| CL173C2 | 918 | No significant similarity found | 0% | 0% |
| CL173C2 | 918 | No significant similarity found(Somewhat similar sequences (blastn)) | 0% | 0% |
| CL174C8 | 1320 | Triticum aestivum chromosome 3B specific BAC library, BAC clone TaaCsp3BFhA_0147D05 | 3% | 90% |
| CL175C17 | 1837 | No significant similarity found | 0% | 0% |
| CL175C17 | 1837 | No significant similarity found(Somewhat similar sequences (blastn)) | 0% | 0% |
| CL176C11 | 1988 | Avena sativa receptor-like kinase extracellular domain rlk6a2 pseudogene, complete sequence | 37% | 77% |
| CL177C26 | 1417 | No significant similarity found | 0% | 0% |
| CL177C26 | 1417 | Brachypodium sylvaticum genomic construct, BACs 73P1 and 17E7 (Somewhat similar sequences (blastn)) | 11% | 73% |
| CL178C9 | 1352 | Triticum aestivum chromosome 3B, genomic scaffold, cultivar Chinese Spring | 4% | 90% |
| CL179C9 | 3908 | Triticum aestivum clone BAC 1551N13, complete sequence | 12% | 78% |
| CL180C8 | 1754 | Avena maroccana clone Amar09 5S ribosomal RNA gene, partial sequence | 99% | 99% |
| CL181C6 | 2140 | No significant similarity found | 0% | 0% |
| CL181C6 | 2140 | PREDICTED: Aegilops tauschii subsp. tauschii pseudouridylate synthase 7 homolog (LOC109740888), transcript variant X7, mRNA (Somewhat similar sequences (blastn)) | 12% | 74% |
| CL182C9 | 3964 | Oryza minuta isolate BC_B Shattering4 gene locus, complete sequence | 92% | 81% |
| CL183C7 | 3411 | PREDICTED: Phalaenopsis equestris protein SOMBRERO (LOC110029730), mRNA | 1% | 94% |
| CL184C8 | 1317 | Aegilops tauschii chromosome 1Ds prolamin gene locus, complete sequence | 25% | 79% |
| CL185C5 | 2197 | No significant similarity found | 0% | 0% |
| CL185C5 | 2197 | Syphacia muris genome assembly S_muris_Valencia ,scaffold SMUV_scaffold0000527 (Somewhat similar sequences (blastn)) | 2% | 85% |
| CL186C6 | 1794 | No significant similarity found | 0% | 0% |
| CL186C6 | 1794 | Triticum aestivum chromosome 3B, genomic scaffold, cultivar Chinese Spring (Somewhat similar sequences (blastn)) | 3% | 87% |
| CL187C2 | 842 | No significant similarity found | 0% | 0% |
| CL187C2 | 842 | Festuca pratensis satellite TR4 sequence (Somewhat similar sequences (blastn)) | 77% | 75% |
| CL188C9 | 999 | Avena strigosa beta-amyrin synthase (Sad1) and cytochrome P450 CYP51H10 (Sad2) genes, complete cds | 28% | 70% |
| CL189C4 | 1705 | No significant similarity found | 0% | 0% |
| CL189C4 | 1705 | PREDICTED: Eucalyptus grandis RNA-binding protein 38 (LOC104450347), transcript variant X2, mRNA (Somewhat similar sequences (blastn)) | 2% | 92% |
| CL190C18 | 1303 | Hordeum vulgare BAC 184G9, complete sequece | 14% | 87% |
| CL191C8 | 963 | No significant similarity found | 0% | 0% |
| CL191C8 | 963 | Triticum aestivum chromosome 3B, genomic scaffold, cultivar Chinese Spring (Somewhat similar sequences (blastn)) | 16% | 84% |
| CL192C6 | 1739 | No significant similarity found | 0% | 0% |
| CL192C6 | 1739 | A.sativa rDNA spacer (Somewhat similar sequences (blastn)) | 88% | 64% |
| CL193C2 | 2681 | No significant similarity found | 0% | 0% |
| CL193C2 | 2681 | Triticum aestivum chromosome 3B, genomic scaffold, cultivar Chinese Spring (Somewhat similar sequences (blastn)) | 100% | 64% |
| CL194C5 | 2065 | No significant similarity found | 0% | 0% |
| CL194C5 | 2065 | Triticum aestivum chromosome 3B, genomic scaffold, cultivar Chinese Spring (Somewhat similar sequences (blastn)) | 5% | 77% |
| CL195C6 | 1817 | No significant similarity found | 0% | 0% |
| CL195C6 | 1817 | Triticum aestivum chromosome 3B, genomic scaffold, cultivar Chinese Spring (Somewhat similar sequences (blastn)) | 98% | 67% |
| CL196C1 | 765 | No significant similarity found | 0% | 0% |
| CL196C1 | 765 | Triticum aestivum chromosome 3B, genomic scaffold, cultivar Chinese Spring (Somewhat similar sequences (blastn)) | 37% | 71% |
| CL197C7 | 1491 | No significant similarity found | 0% | 0% |
| CL197C7 | 1491 | Enterobius vermicularis genome assembly E_vermicularis_Canary_Islands ,scaffold EVEC_scaffold0000030 (Somewhat similar sequences (blastn)) | 4% | 77% |
| CL198C7 | 4077 | Hordeum vulgare clone HV_Mba152-O10, complete sequence | 100% | 97% |
| CL199C2 | 1113 | No significant similarity found | 0% | 0% |
| CL199C2 | 1113 | Vigna angularis var. angularis DNA, chromosome 2, almost complete sequence, cultivar: Shumari (Somewhat similar sequences (blastn)) | 19% | 84% |
| CL200C3 | 1772 | No significant similarity found | 0% | 0% |
| CL200C3 | 1772 | Triticum aestivum chromosome 3B, genomic scaffold, cultivar Chinese Spring (Somewhat similar sequences (blastn)) | 100% | 68% |
| CL201C13 | 1747 | Avena sativa clone OP9 receptor kinase gene cluster, complete sequence | 10% | 95% |
| CL202C13 | 1270 | Triticum aestivum chromosome 3B, genomic scaffold, cultivar Chinese Spring | 78% | 72% |
| CL203C6 | 1983 | No significant similarity found | 0% | 0% |
| CL203C6 | 1983 | Festuca pratensis partial putative non-autonomous helitron with gi pseudogene fragment, clone Fp-psGI.1 (Somewhat similar sequences (blastn)) | 11% | 78% |
| CL204C6 | 1956 | No significant similarity found | 0% | 0% |
| CL204C6 | 1956 | Triticum aestivum chromosome 3B, genomic scaffold, cultivar Chinese Spring (Somewhat similar sequences (blastn)) | 99% | 70% |
| CL205C14 | 1805 | Avena sativa clone OP9 receptor kinase gene cluster, complete sequence | 8% | 78% |
| CL206C2 | 1503 | No significant similarity found | 0% | 0% |
| CL206C2 | 1503 | Triticum aestivum clone pTa-885 FISH-positive repetitive sequence (Somewhat similar sequences (blastn)) | 94% | 70% |
| CL207C8 | 1578 | Oryza officinalis clone OO__Ba0092L04, complete sequence | 1% | 100% |
| CL208C1 | 812 | No significant similarity found | 0% | 0% |
| CL208C1 | 812 | Avena strigosa obtusifoliol 14alpha-demethylase (Cyp51G1) gene, complete cds (Somewhat similar sequences (blastn)) | 18% | 84% |
| CL209C2 | 889 | No significant similarity found | 0% | 0% |
| CL209C2 | 889 | Gregarina niphandrodes hypothetical protein partial mRNA (Somewhat similar sequences (blastn)) | 23% | 71% |
| CL210C7 | 1363 | Festuca glaucescens x Lolium multiflorum microsatellite DNA, clone B2-H3 | 9% | 97% |
| CL211C1 | 3434 | No significant similarity found | 0% | 0% |
| CL211C1 | 3434 | Drosophila melanogaster chromosome 3L (Somewhat similar sequences (blastn)) | 1% | 84% |
| CL212C5 | 906 | No significant similarity found | 0% | 0% |
| CL212C5 | 906 | Drosophila melanogaster chromosome 3L (Somewhat similar sequences (blastn)) | 1% | 86% |
| CL213C3 | 2221 | PREDICTED: Brachypodium distachyon serine/threonine-protein phosphatase 7 long form homolog (LOC100821175), transcript variant X6, misc_RNA | 8% | 79% |
| CL214C11 | 1325 | No significant similarity found | 0% | 0% |
| CL214C11 | 1325 | Triticum aestivum chromosome 3B, genomic scaffold, cultivar Chinese Spring (Somewhat similar sequences (blastn)) | 39% | 72% |
| ***Avena hirtula* Lag. (299; 2*x* = 14; AA)** | | | | |
| CL1C15 | 2761 | Avena strigosa beta-amyrin synthase (Sad1) and cytochrome P450 CYP51H10 (Sad2) genes, complete cds | 91% | 90% |
| CL2C44 | 1569 | Avena strigosa beta-amyrin synthase (Sad1) and cytochrome P450 CYP51H10 (Sad2) genes, complete cds | 99% | 90% |
| CL3C28 | 4201 | Triticum aestivum chromosome 3B, genomic scaffold, cultivar Chinese Spring | 83% | 76% |
| CL4C7 | 2278 | No significant similarity found | 0% | 0% |
| CL4C7 | 2278 | Triticum aestivum chromosome 3B, genomic scaffold, cultivar Chinese Spring | 22% | 87% |
| CL5C139 | 2025 | Avena strigosa beta-amyrin synthase (Sad1) and cytochrome P450 CYP51H10 (Sad2) genes, complete cds | 67% | 94% |
| CL6C204 | 1478 | Avena vaviloviana partial dispersed repeat, clone pAvKB32 | 31% | 85% |
| CL7C110 | 1493 | Avena strigosa cytochrome P450 CYP51H11 (Cyp51H11) gene, complete cds | 89% | 86% |
| CL8C251 | 3933 | Triticum aestivum chromosome 3B, genomic scaffold, cultivar Chinese Spring | 2% | 82% |
| CL9C87 | 2217 | Lolium perenne retrotransposon Lotte_7D23_1 and transposon retrotransposon Camilla_7D23_1, complete sequence; and VRN1 (Lp_7D23_1) gene, complete cds | 94% | 77% |
| CL10C165 | 1931 | Avena strigosa beta-amyrin synthase (Sad1) and cytochrome P450 CYP51H10 (Sad2) genes, complete cds | 91% | 92% |
| CL11C57 | 1786 | Avena sativa clone OP45 receptor kinase gene cluster, complete sequence | 56% | 86% |
| CL12C115 | 3646 | Avena sativa receptor-like kinase extracellular domain rlk2a13 pseudogene, complete sequence | 17% | 81% |
| CL13C206 | 2656 | Triticum aestivum chromosome 3B, genomic scaffold, cultivar Chinese Spring | 6% | 77% |
| CL14C131 | 4835 | Hordeum vulgare clone HV_Mba113-M07, complete sequence | 15% | 72% |
| CL15C95 | 5590 | Avena strigosa beta-amyrin synthase (Sad1) and cytochrome P450 CYP51H10 (Sad2) genes, complete cds | 80% | 83% |
| CL16C31 | 3517 | Triticum aestivum chromosome 3B, genomic scaffold, cultivar Chinese Spring | 100% | 79% |
| CL17C52 | 1342 | Avena strigosa beta-amyrin synthase (Sad1) and cytochrome P450 CYP51H10 (Sad2) genes, complete cds | 99% | 93% |
| CL18C1 | 11478 | No significant similarity found | 0% | 0% |
| CL18C1 | 11478 | Festuca pratensis satellite TR4 sequence(Somewhat similar sequences (blastn)) | 19% | 75% |
| CL19C33 | 2029 | Avena strigosa beta-amyrin synthase (Sad1) and cytochrome P450 CYP51H10 (Sad2) genes, complete cds | 100% | 97% |
| CL20C80 | 1600 | Avena sativa clone OP45 receptor kinase gene cluster, complete sequence | 83% | 87% |
| CL21C79 | 1864 | Avena strigosa beta-amyrin synthase (Sad1) and cytochrome P450 CYP51H10 (Sad2) genes, complete cds | 33% | 92% |
| CL22C12 | 4338 | Thinopyrum intermedium repetitive DNA sequence 1 | 10% | 74% |
| CL23C12 | 1235 | Avena strigosa cytochrome P450 CYP51H11 (Cyp51H11) gene, complete cds | 36% | 84% |
| CL24C78 | 1045 | Avena strigosa beta-amyrin synthase (Sad1) and cytochrome P450 CYP51H10 (Sad2) genes, complete cds | 98% | 90% |
| CL25C177 | 2800 | Phyllostachys edulis clone 00059 putative retrotransposon protein gene, complete cds | 3% | 84% |
| CL26C43 | 3209 | Oryza sativa Indica Group cultivar Shuhui498 chromosome 7 sequence | 35% | 72% |
| CL27C8 | 1866 | Avena strigosa beta-amyrin synthase (Sad1) and cytochrome P450 CYP51H10 (Sad2) genes, complete cds | 99% | 96% |
| CL28C86 | 1991 | Avena sativa clone OP45 receptor kinase gene cluster, complete sequence | 58% | 85% |
| CL29C7 | 2361 | Oryza sativa Indica Group cultivar Shuhui498 chromosome 7 sequence | 39% | 75% |
| CL30C15 | 3474 | Triticum aestivum chromosome 3B, genomic scaffold, cultivar Chinese Spring | 3% | 90% |
| CL31C6 | 2413 | Avena strigosa pAs93 repetitive DNA sequence | 18% | 94% |
| CL32C25 | 2444 | Avena strigosa beta-amyrin synthase (Sad1) and cytochrome P450 CYP51H10 (Sad2) genes, complete cds | 99% | 94% |
| CL33C1 | 1501 | Avena sativa LTR-retrotransposon OARE-1 gag-pol pseudogene for polyprotein | 87% | 97% |
| CL34C71 | 4976 | No significant similarity found | 0% | 0% |
| CL34C71 | 4976 | Triticum aestivum chromosome 3B, genomic scaffold, cultivar Chinese Spring(Somewhat similar sequences (blastn)) | 99% | 68% |
| CL35C101 | 3684 | Triticum aestivum chromosome 3B, genomic scaffold, cultivar Chinese Spring | 44% | 76% |
| CL36C54 | 4743 | Brachypodium distachyon retrotransposon Copia-like REBd3, complete sequence | 93% | 91% |
| CL37C26 | 1754 | Avena sativa clone OP45 receptor kinase gene cluster, complete sequence | 29% | 85% |
| CL38C156 | 2895 | Avena strigosa pAs111 repetitive DNA sequence | 13% | 80% |
| CL39C15 | 2572 | Avena sativa isolate Pc68LrkB2 sequence containing transposon and repetitive DNA linked to receptor kinase gene | 39% | 93% |
| CL40C78 | 1474 | Avena strigosa beta-amyrin synthase (Sad1) and cytochrome P450 CYP51H10 (Sad2) genes, complete cds | 27% | 77% |
| CL41C4 | 3837 | No significant similarity found | 0% | 0% |
| CL41C4 | 3837 | Actinoplanes missouriensis 431 DNA, complete genome(Somewhat similar sequences (blastn)) | 1% | 81% |
| CL42C157 | 4423 | Triticum aestivum chromosome 3B, genomic scaffold, cultivar Chinese Spring | 33% | 72% |
| CL43C16 | 2175 | Avena sativa LTR-retrotransposon OARE-1 gag-pol pseudogene for polyprotein | 100% | 95% |
| CL44C9 | 1945 | Avena strigosa beta-amyrin synthase (Sad1) and cytochrome P450 CYP51H10 (Sad2) genes, complete cds | 97% | 97% |
| CL45C151 | 4668 | Avena sativa cultivar Aslak REMAP marker A002-AC9G genomic sequence | 1% | 96% |
| CL46C65 | 8037 | Triticum aestivum chromosome 3B, genomic scaffold, cultivar Chinese Spring | 42% | 71% |
| CL47C10 | 2642 | Avena sativa LTR-retrotransposon OARE-1 gag-pol pseudogene mRNA for polyprotein | 100% | 97% |
| CL48C97 | 3522 | A.sativa GLAV 3 gene for 11S globulin | 10% | 88% |
| CL49C103 | 3112 | Panicum virgatum clone PV_ABa006-D05, complete sequence | 33% | 74% |
| CL50C52 | 8298 | Triticum aestivum chromosome 3B, genomic scaffold, cultivar Chinese Spring | 7% | 72% |
| CL51C7 | 2179 | Allium sativum microsatellite Asa59 sequence | 8% | 91% |
| CL52C37 | 2640 | No significant similarity found | 0% | 0% |
| CL52C37 | 2640 | Agrostis transcaspica clone AtransGAAT21_insert microsatellite AgrosSSR9 sequences(Somewhat similar sequences (blastn)) | 6% | 72% |
| CL53C64 | 2242 | Avena strigosa DNA for dispersed repeat region, clone As22 | 32% | 98% |
| CL54C142 | 6443 | Triticum aestivum chromosome 3B, genomic scaffold, cultivar Chinese Spring | 26% | 76% |
| CL55C3 | 35834 | Avena sterilis chloroplast, complete genome | 99% | 99% |
| CL56C34 | 1592 | Avena fatua dormancy-associated clone AFD2 putative ORF1 mRNA, 3'UTR | 23% | 95% |
| CL57C78 | 4160 | Triticum aestivum chromosome 3B, genomic scaffold, cultivar Chinese Spring | 85% | 78% |
| CL58C135 | 2944 | Triticum aestivum chromosome 3B, genomic scaffold, cultivar Chinese Spring | 21% | 72% |
| CL59C67 | 2180 | Avena strigosa beta-amyrin synthase (Sad1) and cytochrome P450 CYP51H10 (Sad2) genes, complete cds | 37% | 81% |
| CL60C55 | 2345 | Avena strigosa beta-amyrin synthase (Sad1) and cytochrome P450 CYP51H10 (Sad2) genes, complete cds | 86% | 91% |
| CL61C4 | 4414 | Triticum aestivum chromosome 3B, genomic scaffold, cultivar Chinese Spring | 89% | 84% |
| CL62C35 | 2207 | Triticum aestivum chromosome 3B, genomic scaffold, cultivar Chinese Spring | 6% | 94% |
| CL63C8 | 11129 | Secale cereale external transcribed spacer, 18S ribosomal RNA gene, internal transcribed spacer 1, 5.8S ribosomal RNA gene, and internal transcribed spacer 2, complete sequence; and 26S ribosomal RNA gene, partial sequence | 52% | 97% |
| CL64C16 | 4572 | Triticum aestivum chromosome 3B, genomic scaffold, cultivar Chinese Spring | 76% | 75% |
| CL65C65 | 1279 | Avena strigosa beta-amyrin synthase (Sad1) and cytochrome P450 CYP51H10 (Sad2) genes, complete cds | 89% | 81% |
| CL66C3 | 1732 | Avena sativa LTR-retrotransposon OARE-1 gag-pol pseudogene for polyprotein | 100% | 98% |
| CL67C45 | 6304 | Zea mays BAC clone CH201-151G9 from chromosome 5, complete sequence | 94% | 81% |
| CL68C102 | 5093 | Triticum aestivum chromosome 3B, genomic scaffold, cultivar Chinese Spring | 58% | 78% |
| CL69C60 | 1145 | Erycina pusilla MADS18 (MADS18) gene, complete cds | 2% | 97% |
| CL70C9 | 1573 | Cyperus rotundus clone Cyr_624 SSR marker genomic sequence | 15% | 75% |
| CL71C56 | 5558 | No significant similarity found | 0% | 0% |
| CL71C56 | 5558 | Triticum aestivum chromosome 3B, genomic scaffold, cultivar Chinese Spring(Somewhat similar sequences (blastn)) | 99% | 66% |
| CL72C83 | 1565 | No significant similarity found | 0% | 0% |
| CL72C83 | 1565 | Avena strigosa beta-amyrin synthase (Sad1) and cytochrome P450 CYP51H10 (Sad2) genes, complete cds(Somewhat similar sequences (blastn)) | 66% | 81% |
| CL73C113 | 3317 | Triticum aestivum chromosome 3B, genomic scaffold, cultivar Chinese Spring | 4% | 82% |
| CL74C62 | 4638 | Triticum aestivum chromosome 3B, genomic scaffold, cultivar Chinese Spring | 28% | 68% |
| CL75C28 | 1449 | Lolium perenne retrotransposon Lotte_7D23_1 and transposon retrotransposon Camilla_7D23_1, complete sequence; and VRN1 (Lp_7D23_1) gene, complete cds | 71% | 87% |
| CL76C7 | 2818 | Avena sterilis cultivar PI267989 clone 1 retrotransposon Cereba, complete sequence | 44% | 98% |
| CL77C25 | 2358 | Brachypodium distachyon clone BAC DH037O21, complete sequence | 98% | 79% |
| CL78C71 | 1137 | Avena strigosa beta-amyrin synthase (Sad1) and cytochrome P450 CYP51H10 (Sad2) genes, complete cds | 100% | 90% |
| CL79C149 | 2997 | Triticum aestivum chromosome 3B, genomic scaffold, cultivar Chinese Spring | 13% | 72% |
| CL80C48 | 3921 | No significant similarity found | 0% | 0% |
| CL80C48 | 3921 | Triticum aestivum chromosome 3B, genomic scaffold, cultivar Chinese Spring(Somewhat similar sequences (blastn)) | 96% | 61% |
| CL81C103 | 4819 | Triticum aestivum chromosome 3B, genomic scaffold, cultivar Chinese Spring | 73% | 75% |
| CL82C142 | 3228 | Triticum aestivum chromosome 3B, genomic scaffold, cultivar Chinese Spring | 3% | 88% |
| CL83C37 | 6314 | Avena strigosa beta-amyrin synthase (Sad1) and cytochrome P450 CYP51H10 (Sad2) genes, complete cds | 92% | 87% |
| CL84C226 | 1921 | Triticum aestivum chromosome 3B, genomic scaffold, cultivar Chinese Spring | 3% | 97% |
| CL85C142 | 2519 | No significant similarity found | 0% | 0% |
| CL85C142 | 2519 | PREDICTED: Scleropages formosus cyclic nucleotide-gated cation channel beta-3-like (LOC108927603), mRNA(Somewhat similar sequences (blastn)) | 1% | 88% |
| CL86C16 | 1470 | Avena strigosa beta-amyrin synthase (Sad1) and cytochrome P450 CYP51H10 (Sad2) genes, complete cds | 87% | 79% |
| CL87C34 | 5898 | Brachypodium distachyon retrotransposon Gypsy CRBd2, complete sequence | 84% | 85% |
| CL88C16 | 2872 | Avena strigosa beta-amyrin synthase (Sad1) and cytochrome P450 CYP51H10 (Sad2) genes, complete cds | 2% | 89% |
| CL89C110 | 6119 | Avena strigosa obtusifoliol 14alpha-demethylase (Cyp51G1) gene, complete cds | 1% | 94% |
| CL90C66 | 1314 | Avena strigosa beta-amyrin synthase (Sad1) and cytochrome P450 CYP51H10 (Sad2) genes, complete cds | 99% | 89% |
| CL91C37 | 2615 | Triticum aestivum chromosome 3B, genomic scaffold, cultivar Chinese Spring | 35% | 77% |
| CL92C35 | 7635 | Triticum aestivum chromosome 3B, genomic scaffold, cultivar Chinese Spring | 37% | 74% |
| CL93C131 | 1863 | Triticum aestivum chromosome 3B, genomic scaffold, cultivar Chinese Spring | 4% | 92% |
| CL94C40 | 4398 | No significant similarity found | 0% | 0% |
| CL94C40 | 4398 | Triticum aestivum chromosome 3B, genomic scaffold, cultivar Chinese Spring (Somewhat similar sequences (blastn)) | 99% | 66% |
| CL95C10 | 1447 | Brachypodium distachyon clone BAC DH037O21, complete sequence | 98% | 84% |
| CL96C40 | 7964 | Triticum aestivum chromosome 3B, genomic scaffold, cultivar Chinese Spring | 71% | 76% |
| CL97C181 | 2164 | No significant similarity found | 0% | 0% |
| CL97C181 | 2164 | Triticum aestivum chromosome 3B, genomic scaffold, cultivar Chinese Spring (Somewhat similar sequences (blastn)) | 6% | 88% |
| CL98C19 | 2044 | No significant similarity found | 0% | 0% |
| CL98C19 | 2044 | Phyllostachys edulis clone 00004 putative retrotransposon protein gene, partial cds (Somewhat similar sequences (blastn)) | 4% | 82% |
| CL99C60 | 1582 | Triticum aestivum chromosome 3B, genomic scaffold, cultivar Chinese Spring | 64% | 79% |
| CL100C9 | 1777 | Triticum aestivum chromosome 3B, genomic scaffold, cultivar Chinese Spring | 70% | 72% |
| CL101C26 | 4105 | Avena strigosa beta-amyrin synthase (Sad1) and cytochrome P450 CYP51H10 (Sad2) genes, complete cds | 95% | 97% |
| CL102C71 | 2840 | No significant similarity found | 0% | 0% |
| CL102C71 | 2840 | Oryza sativa Indica Group cultivar Shuhui498 chromosome 1 sequence (Somewhat similar sequences (blastn)) | 55% | 67% |
| CL103C18 | 2416 | Hordeum vulgare cultivar Cepada capa Rym4 and MCT-1 genes, complete cds | 31% | 79% |
| CL104C6 | 3680 | No significant similarity found | 0% | 0% |
| CL104C6 | 3680 | Agrostis stolonifera microsatellite AsNJGT602 sequence (Somewhat similar sequences (blastn)) | 26% | 70% |
| CL105C1 | 4571 | Avena strigosa beta-amyrin synthase (Sad1) and cytochrome P450 CYP51H10 (Sad2) genes, complete cds | 96% | 96% |
| CL106C11 | 2420 | Triticum aestivum chromosome 3B, genomic scaffold, cultivar Chinese Spring | 18% | 76% |
| CL107C55 | 5187 | Triticum aestivum chromosome 3B, genomic scaffold, cultivar Chinese Spring | 2% | 83% |
| CL108C1 | 20132 | Avena sterilis chloroplast, complete genome | 100% | 99% |
| CL109C22 | 3298 | Triticum aestivum chromosome 3B, genomic scaffold, cultivar Chinese Spring | 80% | 73% |
| CL110C40 | 3278 | Lolium perenne retrotransposon Lotte_7D23_1 and transposon retrotransposon Camilla_7D23_1, complete sequence; and VRN1 (Lp_7D23_1) gene, complete cds | 1% | 98% |
| CL111C21 | 5695 | Triticum aestivum chromosome 3B, genomic scaffold, cultivar Chinese Spring | 86% | 87% |
| CL112C37 | 3694 | Triticum aestivum chromosome 3B, genomic scaffold, cultivar Chinese Spring | 63% | 76% |
| CL113C34 | 1817 | Brachypodium distachyon retrotransposon Gypsy CRBd1, complete sequence | 4% | 90% |
| CL114C23 | 1509 | Triticum aestivum chromosome 3B, genomic scaffold, cultivar Chinese Spring | 11% | 79% |
| CL115C1 | 19927 | Avena sterilis chloroplast, complete genome | 99% | 99% |
| CL116C6 | 5867 | Triticum aestivum chromosome 3B, genomic scaffold, cultivar Chinese Spring | 83% | 82% |
| CL117C48 | 3623 | No significant similarity found | 0% | 0% |
| CL117C48 | 3623 | Triticum aestivum chromosome 3B, genomic scaffold, cultivar Chinese Spring (Somewhat similar sequences (blastn)) | 1% | 80% |
| CL118C8 | 4198 | No significant similarity found | 0% | 0% |
| CL118C8 | 4198 | Festuca pratensis satellite TR4 sequence (Somewhat similar sequences (blastn)) | 80% | 67% |
| CL119C1 | 15573 | Avena sativa voucher CAN:Saarela 775 plastid, complete genome | 100% | 99% |
| CL120C46 | 2253 | No significant similarity found | 0% | 0% |
| CL120C46 | 2253 | PREDICTED: Aegilops tauschii subsp. tauschii uncharacterized LOC109736966 (LOC109736966), mRNA (Somewhat similar sequences (blastn)) | 34% | 68% |
| CL121C16 | 3223 | No significant similarity found | 0% | 0% |
| CL121C16 | 3223 | Triticum aestivum chromosome 3B, genomic scaffold, cultivar Chinese Spring (Somewhat similar sequences (blastn)) | 100% | 68% |
| CL122C11 | 1161 | Triticum aestivum chromosome 3B, genomic scaffold, cultivar Chinese Spring | 13% | 87% |
| CL123C3 | 3239 | No significant similarity found | 0% | 0% |
| CL123C3 | 3239 | Triticum aestivum chromosome 3B, genomic scaffold, cultivar Chinese Spring (Somewhat similar sequences (blastn)) | 99% | 65% |
| CL124C26 | 4615 | Triticum aestivum chromosome 3B, genomic scaffold, cultivar Chinese Spring | 71% | 79% |
| CL125C7 | 2412 | No significant similarity found | 0% | 0% |
| CL125C7 | 2412 | H.pratense satellite DNA (ID pPRA1_3) (Somewhat similar sequences (blastn)) | 21% | 79% |
| CL126C1 | 1872 | Avena sativa receptor-like kinase extracellular domain rlk6a2 pseudogene, complete sequence | 100% | 91% |
| CL127C5 | 4918 | Triticum aestivum chromosome 3B, genomic scaffold, cultivar Chinese Spring | 85% | 87% |
| CL128C2 | 4148 | Triticum aestivum chromosome 3B, genomic scaffold, cultivar Chinese Spring | 68% | 80% |
| CL129C15 | 4614 | Avena sativa cultivar Aslak REMAP marker A002-AC9G genomic sequence | 13% | 96% |
| CL130C15 | 3633 | Triticum turgidum subsp. durum Pm3 locus, genomic sequence | 43% | 75% |
| CL131C5 | 2392 | Triticum aestivum chromosome 3B, genomic scaffold, cultivar Chinese Spring | 4% | 97% |
| CL132C1 | 3238 | No significant similarity found | 0% | 0% |
| CL132C1 | 3238 | Select seq emb\|HG670306.1\| Triticum aestivum chromosome 3B, genomic scaffold, cultivar Chinese Spring (Somewhat similar sequences (blastn)) | 32% | 38% |
| CL133C12 | 1507 | No significant similarity found | 0% | 0% |
| CL133C12 | 1507 | Festuca arundinacea genomic FaH13 repeat element (Somewhat similar sequences (blastn)) | 5% | 78% |
| CL134C3 | 4248 | Hordeum vulgare BAC CC24_14, complete sequence | 42% | 75% |
| CL135C11 | 3608 | No significant similarity found | 0% | 0% |
| CL135C11 | 3608 | Brachypodium sylvaticum hypothetical protein (57h21.1), methyl sulfoxide reductase (57h21.2), hypothetical protein (57h21.3), oxidoreductase (57h21.4), auxin efflux carrier (57h21.6), CCR4 associated factor-like protein (57h21.7), ribosomal subunit 8E protein (57h21.8), expressed protein (57h21.9), expressed protein (57h21.10), acid phosphatase (57h21.11), polygalacturonase inhibitor (57h21.12), proteasome (57h21.13), leucine carboxyl methyl transferase (57h21.14), homeobox (57h21.15), serine threonine kinase (57h21.16), hypothetical protein (57h21.17), GTP binding protein (57h21.18), zinc carboxy peptidase (57h21.20), DNA repair protein (57h21.21), coatomer complex subunit (57h21.22), sulfate transporter (57h21.23), hypothetical protein (57h21.24), hypothetical protein (57h21.25), hypothetical protein (57h21.26), acyl-ATP thioesterase (57h21.27), hypothetical protein (57h21.28), hypothetical protein (57h21.29), superoxide dismutase (57h21.30), expressed protein (57h21.31), protein kinase (57h21.32), early nodulin protein (57h21.33), early nodulin protein (57h21.34), hypothetical protein (57h21.36), hypothetical protein (57h21.37), and expressed protein (57h21.38) genes, complete cds (Somewhat similar sequences (blastn)) | 50% | 67% |
| CL136C15 | 4128 | Triticum aestivum chromosome 3B, genomic scaffold, cultivar Chinese Spring | 10% | 73% |
| CL137C5 | 3483 | No significant similarity found | 0% | 0% |
| CL137C5 | 3483 | Triticum aestivum chromosome 3B, genomic scaffold, cultivar Chinese Spring (Somewhat similar sequences (blastn)) | 97% | 64% |
| CL138C13 | 2941 | Brachypodium sylvaticum hypothetical protein (57h21.1), methyl sulfoxide reductase (57h21.2), hypothetical protein (57h21.3), oxidoreductase (57h21.4), auxin efflux carrier (57h21.6), CCR4 associated factor-like protein (57h21.7), ribosomal subunit 8E protein (57h21.8), expressed protein (57h21.9), expressed protein (57h21.10), acid phosphatase (57h21.11), polygalacturonase inhibitor (57h21.12), proteasome (57h21.13), leucine carboxyl methyl transferase (57h21.14), homeobox (57h21.15), serine threonine kinase (57h21.16), hypothetical protein (57h21.17), GTP binding protein (57h21.18), zinc carboxy peptidase (57h21.20), DNA repair protein (57h21.21), coatomer complex subunit (57h21.22), sulfate transporter (57h21.23), hypothetical protein (57h21.24), hypothetical protein (57h21.25), hypothetical protein (57h21.26), acyl-ATP thioesterase (57h21.27), hypothetical protein (57h21.28), hypothetical protein (57h21.29), superoxide dismutase (57h21.30), expressed protein (57h21.31), protein kinase (57h21.32), early nodulin protein (57h21.33), early nodulin protein (57h21.34), hypothetical protein (57h21.36), hypothetical protein (57h21.37), and expressed protein (57h21.38) genes, complete cds | 95% | 83% |
| CL139C8 | 1783 | No significant similarity found | 0% | 0% |
| CL139C8 | 1783 | Triticum aestivum chromosome 3B, genomic scaffold, cultivar Chinese Spring (Somewhat similar sequences (blastn)) | 100% | 68% |
| CL140C8 | 1545 | Hordeum vulgare clone HV_Mba329-C16, complete sequence | 47% | 80% |
| CL141C1 | 2391 | No significant similarity found | 0% | 0% |
| CL141C1 | 2391 | Triticum aestivum chromosome 3B, genomic scaffold, cultivar Chinese Spring (Somewhat similar sequences (blastn)) | 15% | 81% |
| CL142C23 | 2423 | Triticum aestivum chromosome 3B, genomic scaffold, cultivar Chinese Spring | 79% | 75% |
| CL143C4 | 2139 | Avena sativa clone OP45 receptor kinase gene cluster, complete sequence | 29% | 78% |
| CL144C7 | 3165 | Allium sativum microsatellite Asa25 sequence | 7% | 95% |
| CL145C8 | 1280 | Avena sativa retrotransposon Cassandra, complete sequence | 99% | 94% |
| CL146C9 | 1202 | Triticum aestivum chromosome 3B, genomic scaffold, cultivar Chinese Spring | 68% | 73% |
| CL147C10 | 5374 | Triticum aestivum chromosome 3B, genomic scaffold, cultivar Chinese Spring | 58% | 85% |
| CL148C22 | 1009 | No significant similarity found | 0% | 0% |
| CL148C22 | 1009 | Apteryx australis mantelli genome assembly AptMant0, scaffold scaffold2513 (Somewhat similar sequences (blastn)) | 4% | 88% |
| CL149C6 | 1344 | No significant similarity found | 0% | 0% |
| CL149C6 | 1344 | Triticum aestivum chromosome 3B, genomic scaffold, cultivar Chinese Spring | 17% | 74% |
| CL150C8 | 3793 | Oryza sativa Indica Group cultivar Shuhui498 chromosome 10 sequence | 1% | 88% |
| CL151C4 | 2464 | Allium sativum microsatellite Asa59 sequence | 8% | 91% |
| CL152C5 | 2645 | No significant similarity found | 0% | 0% |
| CL152C5 | 2645 | Agrostis transcaspica clone AtransGAAT21_insert microsatellite AgrosSSR9 sequences (Somewhat similar sequences (blastn)) | 6% | 72% |
| CL153C27 | 1444 | Avena strigosa DNA for dispersed repeat region, clone As22 | 32% | 98% |
| CL154C1 | 1464 | Poa palustris PgiC protein (PgiC) gene, partial cds | 3% | 95% |
| CL155C4 | 1707 | Triticum aestivum chromosome 3B, genomic scaffold, cultivar Chinese Spring | 3% | 100% |
| CL156C10 | 3541 | Avena barbata clone Abar07 5S ribosomal RNA gene, partial sequence | 97% | 99% |
| CL157C1 | 990 | Triticum aestivum chromosome 3B, genomic scaffold, cultivar Chinese Spring | 7% | 96% |
| CL158C1 | 1427 | Aegilops umbellulata satellite DNA, p25147-146 | 2% | 88% |
| CL158C1 | 1427 | No significant similarity found | 0% | 0% |
| CL159C10 | 2494 | Triticum aestivum chromosome 3B, genomic scaffold, cultivar Chinese Spring (Somewhat similar sequences (blastn)) | 1% | 97% |
| CL160C1 | 1814 | No significant similarity found | 0% | 0% |
| CL160C1 | 1814 | Triticum aestivum chromosome 3B, genomic scaffold, cultivar Chinese Spring (Somewhat similar sequences (blastn)) | 61% | 69% |
| CL161C11 | 2259 | No significant similarity found | 0% | 0% |
| CL161C11 | 2259 | Triticum aestivum chromosome 3B, genomic scaffold, cultivar Chinese Spring | 51% | 70% |
| CL162C4 | 2136 | Triticum aestivum chromosome 3B, genomic scaffold, cultivar Chinese Spring | 7% | 85% |
| CL163C1 | 648 | No significant similarity found | 0% | 0% |
| CL163C1 | 648 | Oryza sativa Indica Group cultivar Shuhui498 chromosome 3 sequence (Somewhat similar sequences (blastn)) | 35% | 71% |
| CL164C1 | 965 | Triticum aestivum chromosome 3B, genomic scaffold, cultivar Chinese Spring | 70% | 78% |
| CL165C13 | 2702 | No significant similarity found | 0% | 0% |
| CL165C13 | 2702 | Triticum aestivum chromosome 3B, genomic scaffold, cultivar Chinese Spring (Somewhat similar sequences (blastn)) | 99% | 70% |
| CL166C7 | 2997 | Triticum aestivum chromosome 3B, genomic scaffold, cultivar Chinese Spring | 6% | 77% |
| CL167C2 | 1054 | No significant similarity found | 0% | 0% |
| CL167C2 | 1054 | Triticum aestivum chromosome 3B, genomic scaffold, cultivar Chinese Spring (Somewhat similar sequences (blastn)) | 75% | 78% |
| CL168C8 | 1505 | Triticum aestivum chromosome 3B, genomic scaffold, cultivar Chinese Spring | 75% | 78% |
| CL169C4 | 1237 | Lolium perenne clone LpSSR027 SSR marker sequence | 13% | 84% |
| CL170C3 | 2590 | No significant similarity found | 0% | 0% |
| CL170C3 | 2590 | Vigna angularis var. angularis DNA, chromosome 6, almost complete sequence, cultivar: Shumari (Somewhat similar sequences (blastn)) | 1% | 86% |
| CL171C17 | 873 | Lolium perenne partial putative non-autonomous helitron with gi pseudogene fragment, clone Lp-psGI.1 | 35% | 74% |
| CL172C4 | 1185 | Hordeum vulgare subsp. vulgare Lks2 gene for putative short internodesfamily transcription factor, complete cds | 8% | 86% |
| CL173C17 | 1183 | PREDICTED: Brachypodium distachyon DNA-directed RNA polymerase V subunit 1 (LOC100825545), mRNA | 2% | 100% |
| CL174C6 | 5209 | Hordeum vulgare clone HV_Mba152-O10, complete sequence | 92% | 98% |
| CL175C4 | 966 | No significant similarity found | 0% | 0% |
| CL175C4 | 966 | Triticum aestivum chromosome 3B, genomic scaffold, cultivar Chinese Spring (Somewhat similar sequences (blastn)) | 14% | 71% |
| CL176C8 | 2284 | No significant similarity found | 0% | 0% |
| CL176C8 | 2284 | Aegilops tauschii chromosome 1Ds prolamin gene locus, complete sequence (Somewhat similar sequences (blastn)) | 22% | 69% |
| CL177C10 | 1357 | Lolium perenne retrotransposon Lotte_7D23_1 and transposon retrotransposon Camilla_7D23_1, complete sequence; and VRN1 (Lp_7D23_1) gene, complete cds | 16% | 77% |
| CL178C8 | 1018 | No significant similarity found | 0% | 0% |
| CL178C8 | 1018 | Hordeum vulgare clone HV_Mba442-A20, complete sequence (Somewhat similar sequences (blastn)) | 47% | 68% |
| CL179C6 | 1361 | Triticum aestivum chromosome 3B, genomic scaffold, cultivar Chinese Spring | 31% | 86% |
| CL180C6 | 3294 | Triticum aestivum chromosome 3B, genomic scaffold, cultivar Chinese Spring | 29% | 75% |
| CL181C8 | 813 | No significant similarity found | 0% | 0% |
| CL181C8 | 813 | Avena sativa clone OP45 receptor kinase gene cluster, complete sequence (Somewhat similar sequences (blastn)) | 14% | 80% |
| CL182C4 | 1264 | No significant similarity found | 0% | 0% |
| CL182C4 | 1264 | Triticum aestivum chromosome 3B, genomic scaffold, cultivar Chinese Spring (Somewhat similar sequences (blastn)) | 10% | 79% |
| CL183C3 | 1592 | Avena strigosa beta-amyrin synthase (Sad1) and cytochrome P450 CYP51H10 (Sad2) genes, complete cds | 24% | 76% |
| CL184C5 | 3374 | Oryza sativa Indica Group cultivar Shuhui498 chromosome 12 sequence | 2% | 83% |
| CL185C1 | 1073 | No significant similarity found | 0% | 0% |
| CL185C1 | 1073 | Arabidopsis thaliana chromosome 2 sequence (Somewhat similar sequences (blastn)) | 6% | 81% |
| CL186C7 | 1058 | No significant similarity found | 0% | 0% |
| CL186C7 | 1058 | Triticum aestivum chromosome 3B, genomic scaffold, cultivar Chinese Spring (Somewhat similar sequences (blastn)) | 100% | 66% |
| CL187C18 | 855 | No significant similarity found | 0% | 0% |
| CL187C18 | 855 | Avena strigosa beta-amyrin synthase (Sad1) and cytochrome P450 CYP51H10 (Sad2) genes, complete cds (Somewhat similar sequences (blastn)) | 38% | 71% |
| CL188C15 | 1145 | [Candida] intermedia strain PYCC 4715 genome assembly, chromosome: I | 3% | 86% |
| CL189C10 | 1973 | Oryza officinalis clone OO__Ba0092L04, complete sequence | 1% | 100% |
| CL190C9 | 1150 | Triticum aestivum chromosome 3B, genomic scaffold, cultivar Chinese Spring | 68% | 79% |
| CL191C1 | 1760 | No significant similarity found | 0% | 0% |
| CL191C1 | 1760 | Triticum aestivum chromosome 3B, genomic scaffold, cultivar Chinese Spring (Somewhat similar sequences (blastn)) | 17% | 77% |
| CL192C1 | 1992 | No significant similarity found | 0% | 0% |
| CL192C1 | 1992 | Triticum aestivum chromosome 3B, genomic scaffold, cultivar Chinese Spring (Somewhat similar sequences (blastn)) | 99% | 66% |
| CL193C1 | 1413 | PREDICTED: Castor canadensis tripartite motif containing 17 (Trim17), mRNA | 2% | 97% |
| CL194C1 | 1605 | No significant similarity found | 0% | 0% |
| CL194C1 | 1605 | Triticum aestivum chromosome 3B, genomic scaffold, cultivar Chinese Spring (Somewhat similar sequences (blastn)) | 100% | 68% |
| CL195C7 | 1040 | Hordeum vulgare subsp. vulgare Lks2 gene for putative short internodesfamily transcription factor, complete cds | 37% | 81% |
| CL196C6 | 1152 | No significant similarity found | 0% | 0% |
| CL196C6 | 1152 | Festuca pratensis satellite TR4 sequence (Somewhat similar sequences (blastn)) | 98% | 75% |
| CL197C9 | 742 | No significant similarity found | 0% | 0% |
| CL197C9 | 742 | Avena strigosa beta-amyrin synthase (Sad1) and cytochrome P450 CYP51H10 (Sad2) genes, complete cds (Somewhat similar sequences (blastn)) | 80% | 76% |
| CL198C6 | 932 | No significant similarity found | 0% | 0% |
| CL198C6 | 932 | Triticum aestivum chromosome 3B, genomic scaffold, cultivar Chinese Spring (Somewhat similar sequences (blastn)) | 34% | 71% |
| ***Avena strigosa* Schreb. (315; 2*x* = 14; AA)** | | | | |
| CL1C85 | 2282 | Avena strigosa beta-amyrin synthase (Sad1) and cytochrome P450 CYP51H10 (Sad2) genes, complete cds | 82% | 84% |
| CL2C42 | 2083 | Avena strigosa beta-amyrin synthase (Sad1) and cytochrome P450 CYP51H10 (Sad2) genes, complete cds | 41% | 81% |
| CL3C256 | 2345 | No significant similarity found | 0% | 0% |
| CL3C256 | 2345 | Triticum aestivum chromosome 3B, genomic scaffold, cultivar Chinese Spring | 16% | 86% |
| CL4C119 | 3424 | Triticum aestivum chromosome 3B, genomic scaffold, cultivar Chinese Spring | 66% | 73% |
| CL5C3 | 449 | Avena strigosa beta-amyrin synthase (Sad1) and cytochrome P450 CYP51H10 (Sad2) genes, complete cds | 78% | 98% |
| CL6C298 | 1413 | Avena vaviloviana partial dispersed repeat, clone pAvKB32 | 32% | 86% |
| CL7C149 | 1975 | Lolium perenne retrotransposon Lotte_7D23_1 and transposon retrotransposon Camilla_7D23_1, complete sequence; and VRN1 (Lp_7D23_1) gene, complete cds | 62% | 93% |
| CL8C26 | 1479 | Avena strigosa cytochrome P450 CYP51H11 (Cyp51H11) gene, complete cds | 89% | 88% |
| CL9C54 | 4000 | Avena strigosa pAs93 repetitive DNA sequence | 16% | 95% |
| CL10C179 | 2455 | Triticum aestivum chromosome 3B, genomic scaffold, cultivar Chinese Spring | 3% | 85% |
| CL11C9 | 1724 | Avena sativa clone OP45 receptor kinase gene cluster, complete sequence | 68% | 87% |
| CL12C150 | 6651 | Triticum aestivum chromosome 3B, genomic scaffold, cultivar Chinese Spring | 29% | 70% |
| CL13C86 | 7447 | Avena strigosa pAs111 repetitive DNA sequence | 8% | 84% |
| CL14C71 | 1800 | Avena strigosa beta-amyrin synthase (Sad1) and cytochrome P450 CYP51H10 (Sad2) genes, complete cds | 99% | 92% |
| CL15C16 | 2029 | Avena strigosa beta-amyrin synthase (Sad1) and cytochrome P450 CYP51H10 (Sad2) genes, complete cds | 99% | 97% |
| CL16C101 | 5430 | Avena strigosa beta-amyrin synthase (Sad1) and cytochrome P450 CYP51H10 (Sad2) genes, complete cds | 81% | 83% |
| CL17C221 | 4680 | Avena sativa receptor-like kinase extracellular domain rlk2a13 pseudogene, complete sequence | 11% | 83% |
| CL18C39 | 6466 | No significant similarity found | 0% | 0% |
| CL18C39 | 6466 | Triticum aestivum chromosome 3B, genomic scaffold, cultivar Chinese Spring (Somewhat similar sequences (blastn)) | 98% | 68% |
| CL19C166 | 2607 | Avena strigosa beta-amyrin synthase (Sad1) and cytochrome P450 CYP51H10 (Sad2) genes, complete cds | 99% | 93% |
| CL20C23 | 1846 | Avena sativa clone OP45 receptor kinase gene cluster, complete sequence | 72% | 85% |
| CL21C4 | 1346 | Avena strigosa beta-amyrin synthase (Sad1) and cytochrome P450 CYP51H10 (Sad2) genes, complete cds | 80% | 85% |
| CL22C4 | 1773 | Avena sativa clone OP45 receptor kinase gene cluster, complete sequence | 27% | 84% |
| CL23C108 | 1439 | Avena strigosa beta-amyrin synthase (Sad1) and cytochrome P450 CYP51H10 (Sad2) genes, complete cds | 70% | 87% |
| CL24C9 | 8699 | Triticum aestivum chromosome 3B, genomic scaffold, cultivar Chinese Spring | 39% | 71% |
| CL25C18 | 2337 | Avena strigosa beta-amyrin synthase (Sad1) and cytochrome P450 CYP51H10 (Sad2) genes, complete cds | 97% | 96% |
| CL26C120 | 1342 | Avena strigosa pAs14 repetitive DNA sequence | 32% | 92% |
| CL27C105 | 5819 | Triticum aestivum chromosome 3B, genomic scaffold, cultivar Chinese Spring | 60% | 80% |
| CL28C78 | 3722 | Triticum aestivum chromosome 3B, genomic scaffold, cultivar Chinese Spring | 1% | 89% |
| CL29C77 | 2641 | Triticum aestivum chromosome 3B, genomic scaffold, cultivar Chinese Spring | 5% | 90% |
| CL30C308 | 2394 | Triticum aestivum chromosome 3B, genomic scaffold, cultivar Chinese Spring | 28% | 75% |
| CL31C73 | 2159 | Avena sativa clone OP45 receptor kinase gene cluster, complete sequence | 53% | 85% |
| CL32C36 | 1909 | Triticum aestivum chromosome 3B, genomic scaffold, cultivar Chinese Spring | 16% | 78% |
| CL33C29 | 6194 | Triticum aestivum chromosome 3B specific BAC library, BAC clone TaaCsp3BFhA_0147D05 | 72% | 81% |
| CL34C2 | 8123 | No significant similarity found | 0% | 0% |
| CL34C2 | 8123 | Bovine viral diarrhea virus 1 genome assembly, chromosome: viral_cRNA (Somewhat similar sequences (blastn)) | 0% | 87% |
| CL35C35 | 1541 | Avena sativa LTR-retrotransposon OARE-1 gag-pol pseudogene for polyprotein | 99% | 97% |
| CL36C159 | 5347 | Triticum aestivum chromosome 3B, genomic scaffold, cultivar Chinese Spring | 15% | 76% |
| CL37C13 | 1511 | Avena strigosa beta-amyrin synthase (Sad1) and cytochrome P450 CYP51H10 (Sad2) genes, complete cds | 40% | 96% |
| CL38C2 | 2408 | Avena strigosa DNA for dispersed repeat region, clone As22 | 29% | 89% |
| CL39C39 | 6052 | Oryza sativa Indica Group cultivar Shuhui498 chromosome 4 sequence | 18% | 71% |
| CL40C126 | 4204 | Triticum aestivum chromosome 3B, genomic scaffold, cultivar Chinese Spring | 41% | 78% |
| CL41C277 | 1381 | No significant similarity found | 0% | 0% |
| CL41C277 | 1381 | Avena sativa clone OP45 receptor kinase gene cluster, complete sequence (Somewhat similar sequences (blastn)) | 15% | 75% |
| CL42C75 | 9204 | Triticum aestivum chromosome 3B, genomic scaffold, cultivar Chinese Spring | 37% | 77% |
| CL43C1 | 2664 | Avena sativa LTR-retrotransposon OARE-1 gag-pol pseudogene for polyprotein | 100% | 98% |
| CL44C65 | 2126 | Allium sativum microsatellite Asa59 sequence | 5% | 93% |
| CL45C77 | 2532 | Avena sativa isolate Pc68LrkB2 sequence containing transposon and repetitive DNA linked to receptor kinase gene | 39% | 94% |
| CL46C1 | 60288 | Avena sterilis chloroplast, complete genome | 99% | 99% |
| CL47C92 | 2964 | A.sativa GLAV 3 gene for 11S globulin | 10% | 90% |
| CL48C21 | 1396 | Avena strigosa beta-amyrin synthase (Sad1) and cytochrome P450 CYP51H10 (Sad2) genes, complete cds | 100% | 95% |
| CL49C25 | 2221 | Avena sativa LTR-retrotransposon OARE-1 gag-pol pseudogene for polyprotein | 99% | 96% |
| CL50C21 | 1608 | Triticum urartu clone BAC 41C8 genomic sequence | 10% | 81% |
| CL51C76 | 3225 | Arthrobacter arilaitensis strain KLBMP5180, complete genome | 0% | 100% |
| CL52C28 | 2918 | No significant similarity found | 0% | 0% |
| CL52C28 | 2918 | Allium sativum microsatellite Asa31 sequence (Somewhat similar sequences (blastn)) | 4% | 80% |
| CL53C31 | 4042 | Panicum virgatum clone PV_ABa107-B06, complete sequence | 79% | 78% |
| CL54C63 | 2754 | Triticum aestivum chromosome 3B, genomic scaffold, cultivar Chinese Spring | 5% | 84% |
| CL55C110 | 2458 | Avena strigosa beta-amyrin synthase (Sad1) and cytochrome P450 CYP51H10 (Sad2) genes, complete cds | 57% | 85% |
| CL56C61 | 3391 | Triticum aestivum chromosome 3B, genomic scaffold, cultivar Chinese Spring | 90% | 79% |
| CL57C15 | 2523 | No significant similarity found | 0% | 0% |
| CL57C15 | 2523 | Agrostis transcaspica clone AtransGAAT21_insert microsatellite AgrosSSR9 sequences (Somewhat similar sequences (blastn)) | 6% | 74% |
| CL58C56 | 1218 | Avena strigosa beta-amyrin synthase (Sad1) and cytochrome P450 CYP51H10 (Sad2) genes, complete cds | 50% | 86% |
| CL59C124 | 5274 | Triticum aestivum chromosome 3B, genomic scaffold, cultivar Chinese Spring | 60% | 82% |
| CL60C38 | 7098 | Triticum aestivum chromosome 3B, genomic scaffold, cultivar Chinese Spring | 13% | 71% |
| CL61C103 | 1706 | Avena strigosa beta-amyrin synthase (Sad1) and cytochrome P450 CYP51H10 (Sad2) genes, complete cds | 94% | 92% |
| CL62C41 | 3707 | Brachypodium distachyon retrotransposon Copia-like REBd3, complete sequence | 92% | 91% |
| CL63C50 | 5241 | Triticum aestivum chromosome 3B, genomic scaffold, cultivar Chinese Spring | 66% | 75% |
| CL64C14 | 2346 | No significant similarity found | 0% | 0% |
| CL64C14 | 2346 | Festuca pratensis partial putative non-autonomous helitron with gi pseudogene fragment, clone Fp-psGI.1 (Somewhat similar sequences (blastn)) | 9% | 78% |
| CL65C50 | 3842 | No significant similarity found | 0% | 0% |
| CL65C50 | 3842 | Triticum aestivum chromosome 3B, genomic scaffold, cultivar Chinese Spring (Somewhat similar sequences (blastn)) | 99% | 64% |
| CL66C90 | 5002 | Avena sativa REMAP marker D004-CAC7T genomic sequence | 10% | 90% |
| CL67C12 | 2377 | Brachypodium distachyon clone BAC DH037O21, complete sequence | 87% | 82% |
| CL68C17 | 4423 | Triticum aestivum chromosome 3B, genomic scaffold, cultivar Chinese Spring | 78% | 78% |
| CL69C200 | 3085 | Triticum aestivum chromosome 3B, genomic scaffold, cultivar Chinese Spring | 1% | 97% |
| CL70C9 | 1604 | No significant similarity found | 0% | 0% |
| CL70C9 | 1604 | Avena strigosa beta-amyrin synthase (Sad1) and cytochrome P450 CYP51H10 (Sad2) genes, complete cds (Somewhat similar sequences (blastn)) | 72% | 82% |
| CL71C12 | 6314 | Secale cereale external transcribed spacer, 18S ribosomal RNA gene, internal transcribed spacer 1, 5.8S ribosomal RNA gene, and internal transcribed spacer 2, complete sequence; and 26S ribosomal RNA gene, partial sequence | 47% | 96% |
| CL72C35 | 4041 | Panicum virgatum clone PV_ABa073-D10, complete sequence | 32% | 75% |
| CL73C4 | 1659 | Avena sativa LTR-retrotransposon OARE-1 gag-pol pseudogene mRNA for polyprotein | 100% | 98% |
| CL74C38 | 7752 | Zea mays BAC clone CH201-151G9 from chromosome 5, complete sequence | 86% | 81% |
| CL75C22 | 2747 | Brachypodium distachyon retrotransposon Gypsy CRBd1, complete sequence | 80% | 75% |
| CL76C100 | 4288 | No significant similarity found (Somewhat similar sequences (blastn)) | 0% | 0% |
| CL76C100 | 4288 | Acinetobacter sp. NCu2D-2, complete genome | 0% | 100% |
| CL77C12 | 5161 | Triticum aestivum chromosome 3B, genomic scaffold, cultivar Chinese Spring | 98% | 62% |
| CL78C2 | 5825 | Triticum aestivum chromosome 3B, genomic scaffold, cultivar Chinese Spring | 94% | 84% |
| CL79C149 | 2101 | Avena strigosa beta-amyrin synthase (Sad1) and cytochrome P450 CYP51H10 (Sad2) genes, complete cds | 60% | 75% |
| CL80C44 | 1301 | No significant similarity found | 0% | 0% |
| CL80C44 | 1301 | Avena vaviloviana partial dispersed repeat, clone pAvKB32 (Somewhat similar sequences (blastn)) | 14% | 80% |
| CL81C13 | 1108 | Avena strigosa beta-amyrin synthase (Sad1) and cytochrome P450 CYP51H10 (Sad2) genes, complete cds | 81% | 78% |
| CL82C114 | 3189 | Triticum aestivum chromosome 3B, genomic scaffold, cultivar Chinese Spring | 14% | 76% |
| CL83C19 | 3486 | Brachypodium distachyon retrotransposon Copia-like REBd3, complete sequence | 92% | 91% |
| CL84C43 | 2015 | Lolium perenne retrotransposon Lotte_7D23_1 and transposon retrotransposon Camilla_7D23_1, complete sequence; and VRN1 (Lp_7D23_1) gene, complete cds | 81% | 81% |
| CL85C3 | 2910 | Triticum aestivum chromosome 3B, genomic scaffold, cultivar Chinese Spring | 51% | 75% |
| CL86C40 | 3005 | Avena strigosa beta-amyrin synthase (Sad1) and cytochrome P450 CYP51H10 (Sad2) genes, complete cds | 2% | 85% |
| CL87C7 | 1455 | No significant similarity found | 0% | 0% |
| CL87C7 | 1455 | Avena strigosa beta-amyrin synthase (Sad1) and cytochrome P450 CYP51H10 (Sad2) genes, complete cds (Somewhat similar sequences (blastn)) | 27% | 68% |
| CL88C5 | 1235 | Avena strigosa beta-amyrin synthase (Sad1) and cytochrome P450 CYP51H10 (Sad2) genes, complete cds | 100% | 92% |
| CL89C24 | 6433 | Festuca pratensis gi pseudogene fragment, clone Fp-psGI.3 | 24% | 82% |
| CL90C82 | 1496 | No significant similarity found | 0% | 0% |
| CL90C82 | 1496 | Triticum aestivum chromosome 3B, genomic scaffold, cultivar Chinese Spring (Somewhat similar sequences (blastn)) | 95% | 70% |
| CL91C7 | 1985 | No significant similarity found | 0% | 0% |
| CL91C7 | 1985 | Nocardia seriolae DNA, complete genome, strain: UTF1 (Somewhat similar sequences (blastn)) | 3% | 75% |
| CL92C30 | 3074 | No significant similarity found | 0% | 0% |
| CL92C30 | 3074 | Triticum aestivum chromosome 3B, genomic scaffold, cultivar Chinese Spring (Somewhat similar sequences (blastn)) | 100% | 67% |
| CL93C8 | 2777 | Triticum aestivum chromosome 3B, genomic scaffold, cultivar Chinese Spring | 42% | 78% |
| CL94C21 | 5477 | Triticum aestivum chromosome 3B, genomic scaffold, cultivar Chinese Spring | 74% | 87% |
| CL95C74 | 2284 | Phyllostachys heterocycla genomic DNA, BAC clone: PH01B015M02, complete sequence | 2% | 94% |
| CL96C7 | 4020 | No significant similarity found | 0% | 0% |
| CL96C7 | 4020 | Zebrafish DNA sequence from clone CH211-286C5 in linkage group 24, complete sequence (Somewhat similar sequences (blastn)) | 4% | 77% |
| CL97C1 | 23092 | Avena sterilis chloroplast, complete genome | 99% | 99% |
| CL98C91 | 1779 | No significant similarity found | 0% | 0% |
| CL98C91 | 1779 | Macaca fascicularis complete genome, chromosome chr2 (Somewhat similar sequences (blastn)) | 3% | 84% |
| CL99C8 | 3818 | Avena strigosa beta-amyrin synthase (Sad1) and cytochrome P450 CYP51H10 (Sad2) genes, complete cds | 99% | 97% |
| CL100C35 | 2305 | Triticum aestivum chromosome 3B, genomic scaffold, cultivar Chinese Spring | 33% | 77% |
| CL101C10 | 2100 | No significant similarity found | 0% | 0% |
| CL101C10 | 2100 | Festuca pratensis satellite TR4 sequence (Somewhat similar sequences (blastn)) | 22% | 74% |
| CL102C31 | 720 | Avena strigosa DNA for tandem repeat (pAs120) | 26% | 89% |
| CL103C34 | 2051 | Triticum aestivum chromosome 3B, genomic scaffold, cultivar Chinese Spring | 8% | 93% |
| CL104C65 | 2049 | No significant similarity found | 0% | 0% |
| CL104C65 | 2049 | PREDICTED: Brachypodium distachyon uncharacterized LOC100827738 (LOC100827738), transcript variant X6, ncRNA (Somewhat similar sequences (blastn)) | 6% | 79% |
| CL105C6 | 3965 | Triticum aestivum chromosome 3B, genomic scaffold, cultivar Chinese Spring | 74% | 73% |
| CL106C12 | 5964 | Triticum aestivum chromosome 3B, genomic scaffold, cultivar Chinese Spring | 82% | 86% |
| CL107C21 | 2683 | Triticum aestivum chromosome 3B, genomic scaffold, cultivar Chinese Spring | 48% | 70% |
| CL108C19 | 4545 | Avena strigosa beta-amyrin synthase (Sad1) and cytochrome P450 CYP51H10 (Sad2) genes, complete cds | 97% | 96% |
| CL109C1 | 2585 | No significant similarity found | 0% | 0% |
| CL109C1 | 2585 | Triticum aestivum chromosome 3B, genomic scaffold, cultivar Chinese Spring (Somewhat similar sequences (blastn)) | 99% | 64% |
| CL110C2 | 16978 | Avena sterilis chloroplast, complete genome | 99% | 99% |
| CL111C19 | 3112 | PREDICTED: Aegilops tauschii subsp. tauschii uncharacterized LOC109787051 (LOC109787051), mRNA | 4% | 78% |
| CL112C39 | 2048 | Triticum aestivum chromosome 3B, genomic scaffold, cultivar Chinese Spring | 11% | 88% |
| CL113C46 | 4589 | No significant similarity found | 0% | 0% |
| CL113C46 | 4589 | Cyperus rotundus clone Cyr_42 SSR marker genomic sequence (Somewhat similar sequences (blastn)) | 5% | 73% |
| CL114C9 | 2836 | Agrostis stolonifera microsatellite AsNJGA1763 sequence | 13% | 86% |
| CL115C7 | 1569 | Triticum aestivum chromosome 3B, genomic scaffold, cultivar Chinese Spring | 96% | 66% |
| CL116C17 | 3467 | Festuca pratensis satellite TR4 sequence | 75% | 68% |
| CL117C7 | 5406 | Triticum aestivum chromosome 3B, genomic scaffold, cultivar Chinese Spring | 90% | 82% |
| CL118C151 | 944 | No significant similarity found | 0% | 0% |
| CL118C151 | 944 | PREDICTED: Solenopsis invicta caveolin-3-like (LOC105194358), transcript variant X4, mRNA (Somewhat similar sequences (blastn)) | 3% | 97% |
| CL119C49 | 1410 | Triticum aestivum chromosome 3B, genomic scaffold, cultivar Chinese Spring | 3% | 10% |
| CL120C30 | 4776 | Triticum turgidum subsp. durum Pm3 locus, genomic sequence | 32% | 75% |
| CL121C5 | 8744 | Avena sativa voucher CAN:Saarela 775 plastid, complete genome | 100% | 99% |
| CL122C6 | 3409 | Triticum aestivum chromosome 3B, genomic scaffold, cultivar Chinese Spring | 78% | 81% |
| CL123C17 | 1210 | Triticum aestivum chromosome 3B, genomic scaffold, cultivar Chinese Spring | 13% | 87% |
| CL124C29 | 2427 | Avena sativa cultivar Aslak REMAP marker A002-AC9G genomic sequence | 17% | 96% |
| CL125C12 | 2791 | H.pratense satellite DNA (ID pPRA1_3) | 11% | 78% |
| CL126C30 | 2048 | Avena sativa retrotransposon Cassandra, complete sequence | 83% | 94% |
| CL127C13 | 1477 | Avena sativa receptor-like kinase extracellular domain rlk6a2 pseudogene, complete sequence | 99% | 93% |
| CL128C4 | 5519 | No significant similarity found | 0% | 0% |
| CL128C4 | 5519 | Triticum aestivum chromosome 3B, genomic scaffold, cultivar Chinese Spring (Somewhat similar sequences (blastn)) | 33% | 70% |
| CL129C10 | 4513 | Triticum aestivum chromosome 3B, genomic scaffold, cultivar Chinese Spring | 94% | 81% |
| CL130C8 | 1886 | No significant similarity found | 0% | 0% |
| CL130C8 | 1886 | Triticum aestivum chromosome 3B, genomic scaffold, cultivar Chinese Spring (Somewhat similar sequences (blastn)) | 97% | 68% |
| CL131C21 | 3304 | No significant similarity found | 0% | 0% |
| CL131C21 | 3304 | Triticum aestivum chromosome 3B, genomic scaffold, cultivar Chinese Spring (Somewhat similar sequences (blastn)) | 47% | 74% |
| CL132C32 | 3125 | Hordeum vulgare BAC CC24_14, complete sequence | 57% | 75% |
| CL133C4 | 5013 | Brachypodium sylvaticum hypothetical protein (57h21.1), methyl sulfoxide reductase (57h21.2), hypothetical protein (57h21.3), oxidoreductase (57h21.4), auxin efflux carrier (57h21.6), CCR4 associated factor-like protein (57h21.7), ribosomal subunit 8E protein (57h21.8), expressed protein (57h21.9), expressed protein (57h21.10), acid phosphatase (57h21.11), polygalacturonase inhibitor (57h21.12), proteasome (57h21.13), leucine carboxyl methyl transferase (57h21.14), homeobox (57h21.15), serine threonine kinase (57h21.16), hypothetical protein (57h21.17), GTP binding protein (57h21.18), zinc carboxy peptidase (57h21.20), DNA repair protein (57h21.21), coatomer complex subunit (57h21.22), sulfate transporter (57h21.23), hypothetical protein (57h21.24), hypothetical protein (57h21.25), hypothetical protein (57h21.26), acyl-ATP thioesterase (57h21.27), hypothetical protein (57h21.28), hypothetical protein (57h21.29), superoxide dismutase (57h21.30), expressed protein (57h21.31), protein kinase (57h21.32), early nodulin protein (57h21.33), early nodulin protein (57h21.34), hypothetical protein (57h21.36), hypothetical protein (57h21.37), and expressed protein (57h21.38) genes, complete cds | 95% | 84% |
| CL134C12 | 2142 | Triticum aestivum chromosome 3B, genomic scaffold, cultivar Chinese Spring | 92% | 80% |
| CL135C4 | 1794 | No significant similarity found | 0% | 0% |
| CL135C4 | 1794 | Avena strigosa DNA for tandem repeat (pAs120) (Somewhat similar sequences (blastn)) | 15% | 73% |
| CL136C29 | 1550 | No significant similarity found | 0% | 0% |
| CL136C29 | 1550 | Festuca arundinacea genomic FaH13 repeat element | 3% | 85% |
| CL137C4 | 1672 | Avena strigosa clone Astr11-Ciav9066 5S ribosomal RNA gene, complete sequence | 96% | 100% |
| CL138C5 | 2114 | No significant similarity found | 0% | 0% |
| CL138C5 | 2114 | Triticum aestivum chromosome 3B, genomic scaffold, cultivar Chinese Spring (Somewhat similar sequences (blastn)) | 59% | 68% |
| CL139C5 | 2155 | No significant similarity found | 0% | 0% |
| CL139C5 | 2155 | Triticum aestivum chromosome 3B, genomic scaffold, cultivar Chinese Spring (Somewhat similar sequences (blastn)) | 99% | 66% |
| CL140C6 | 1595 | PREDICTED: Brachypodium distachyon uncharacterized LOC100821162 (LOC100821162), mRNA | 99% | 98% |
| CL141C6 | 5142 | Hordeum vulgare clone HV_Mba789-L09, complete sequence | 6% | 81% |
| CL142C22 | 2051 | No significant similarity found | 0% | 0% |
| CL142C22 | 2051 | Triticum turgidum subsp. durum cultivar Langdon clone BAC 406B11, complete sequence (Somewhat similar sequences (blastn)) | 21% | 66% |
| CL143C2 | 2142 | Oryza sativa Indica Group cultivar Shuhui498 chromosome 10 sequence | 2% | 88% |
| CL144C5 | 1235 | Triticum aestivum chromosome 3B, genomic scaffold, cultivar Chinese Spring | 5% | 96% |
| CL145C2 | 1895 | Hordeum vulgare clone HV_Mba329-C16, complete sequence | 55% | 78% |
| CL146C7 | 1501 | No significant similarity found | 0% | 0% |
| CL146C7 | 1501 | Elaeophora elaphi genome assembly E_elaphi ,scaffold EEL_contig0000459 (Somewhat similar sequences (blastn)) | 2% | 86% |
| CL147C16 | 1526 | No significant similarity found | 0% | 0% |
| CL147C16 | 1526 | Triticum aestivum chromosome 3B, genomic scaffold, cultivar Chinese Spring (Somewhat similar sequences (blastn)) | 28% | 74% |
| CL148C9 | 2731 | No significant similarity found | 0% | 0% |
| CL148C9 | 2731 | Brachypodium sylvaticum hypothetical protein (57h21.1), methyl sulfoxide reductase (57h21.2), hypothetical protein (57h21.3), oxidoreductase (57h21.4), auxin efflux carrier (57h21.6), CCR4 associated factor-like protein (57h21.7), ribosomal subunit 8E protein (57h21.8), expressed protein (57h21.9), expressed protein (57h21.10), acid phosphatase (57h21.11), polygalacturonase inhibitor (57h21.12), proteasome (57h21.13), leucine carboxyl methyl transferase (57h21.14), homeobox (57h21.15), serine threonine kinase (57h21.16), hypothetical protein (57h21.17), GTP binding protein (57h21.18), zinc carboxy peptidase (57h21.20), DNA repair protein (57h21.21), coatomer complex subunit (57h21.22), sulfate transporter (57h21.23), hypothetical protein (57h21.24), hypothetical protein (57h21.25), hypothetical protein (57h21.26), acyl-ATP thioesterase (57h21.27), hypothetical protein (57h21.28), hypothetical protein (57h21.29), superoxide dismutase (57h21.30), expressed protein (57h21.31), protein kinase (57h21.32), early nodulin protein (57h21.33), early nodulin protein (57h21.34), hypothetical protein (57h21.36), hypothetical protein (57h21.37), and expressed protein (57h21.38) genes, complete cds (Somewhat similar sequences (blastn)) | 64% | 67% |
| CL149C1 | 3406 | Triticum aestivum chromosome 3B, genomic scaffold, cultivar Chinese Spring | 83% | 77% |
| CL150C2 | 2267 | Triticum aestivum chromosome 3B, genomic scaffold, cultivar Chinese Spring | 85% | 55% |
| CL151C2 | 1984 | No significant similarity found | 0% | 0% |
| CL151C2 | 1984 | Triticum aestivum chromosome 3B, genomic scaffold, cultivar Chinese Spring (Somewhat similar sequences (blastn)) | 99% | 68% |
| CL152C5 | 6023 | Hordeum vulgare clone HV_Mba152-O10, complete sequence | 96% | 91% |
| CL153C6 | 1813 | No significant similarity found | 0% | 0% |
| CL153C6 | 1813 | Triticum aestivum chromosome 3B, genomic scaffold, cultivar Chinese Spring (Somewhat similar sequences (blastn)) | 100% | 61% |
| CL154C11 | 1728 | Triticum aestivum chromosome 3B, genomic scaffold, cultivar Chinese Spring | 6% | 81% |
| CL155C10 | 1115 | PREDICTED: Paralichthys olivaceus ST3 beta-galactoside alpha-2,3-sialyltransferase 2 (st3gal2), partial mRNA | 3% | 88% |
| CL156C5 | 4723 | Triticum aestivum chromosome 3B, genomic scaffold, cultivar Chinese Spring | 16% | 74% |
| CL157C3 | 789 | Poa palustris PgiC protein (PgiC) gene, partial cds | 7% | 95% |
| CL158C3 | 2701 | PREDICTED: Sarcophilus harrisii X-linked retinitis pigmentosa GTPase regulator-like (LOC100922304), partial mRNA | 1% | 91% |
| CL159C5 | 2812 | No significant similarity found | 0% | 0% |
| CL159C5 | 2812 | Triticum aestivum chromosome 3B, genomic scaffold, cultivar Chinese Spring (Somewhat similar sequences (blastn)) | 39% | 65% |
| CL160C2 | 5046 | Triticum aestivum chromosome 3B, genomic scaffold, cultivar Chinese Spring | 61% | 85% |
| CL161C19 | 1211 | Avena sativa receptor-like kinase extracellular domain rlk6a2 pseudogene, complete sequence | 62% | 71% |
| CL162C2 | 1204 | Triticum aestivum chromosome 3B, genomic scaffold, cultivar Chinese Spring | 16% | 78% |
| CL163C4 | 6192 | Triticum aestivum chromosome 3B, genomic scaffold, cultivar Chinese Spring | 8% | 78% |
| CL164C1 | 2686 | PREDICTED: Aegilops tauschii subsp. tauschii uncharacterized LOC109761959 (LOC109761959), mRNA | 31% | 75% |
| CL165C1 | 1281 | Oryza sativa Indica Group cultivar Shuhui498 chromosome 9 sequence | 12% | 80% |
| CL166C5 | 1838 | Hordeum vulgare clone HV_Mba727-J05, complete sequence | 2% | 95% |
| CL167C1 | 1360 | Triticum aestivum chromosome 3B, genomic scaffold, cultivar Chinese Spring | 82% | 76% |
| CL168C14 | 1353 | Triticum aestivum chromosome 3B, genomic scaffold, cultivar Chinese Spring | 13% | 86% |
| CL169C5 | 1271 | Triticum aestivum chromosome 3B, genomic scaffold, cultivar Chinese Spring | 4% | 90% |
| CL170C4 | 978 | Agrostis stolonifera microsatellite AsNJGA1428 sequence | 7% | 79% |
| CL171C1 | 770 | No significant similarity found | 0% | 0% |
| CL171C1 | 770 | Lolium perenne partial putative non-autonomous helitron with gi pseudogene fragment, clone Lp-psGI.1 (Somewhat similar sequences (blastn)) | 34% | 72% |
| CL172C3 | 3618 | Triticum aestivum chromosome 3B, genomic scaffold, cultivar Chinese Spring | 64% | 76% |
| CL173C7 | 1088 | Triticum aestivum chromosome 3B, genomic scaffold, cultivar Chinese Spring | 74% | 78% |
| CL174C4 | 1933 | Avena sativa clone OP9 receptor kinase gene cluster, complete sequence | 4% | 88% |
| CL175C5 | 1170 | No significant similarity found | 0% | 0% |
| CL175C5 | 1170 | Avena sativa SCAR marker BG2 genomic sequence (Somewhat similar sequences (blastn)) | 44% | 71% |
| CL176C4 | 1809 | No significant similarity found | 0% | 0% |
| CL176C4 | 1809 | Eutrema salsugineum hypothetical protein (EUTSA_v10015313mg) mRNA, complete cds (Somewhat similar sequences (blastn)) | 2% | 85% |
| CL177C6 | 1310 | No significant similarity found | 0% | 0% |
| CL177C6 | 1310 | PREDICTED: Aegilops tauschii subsp. tauschii uncharacterized LOC109736966 (LOC109736966), mRNA (Somewhat similar sequences (blastn)) | 59% | 68% |
| CL178C2 | 4301 | PREDICTED: Sorghum bicolor protein FAR1-RELATED SEQUENCE 5 (LOC8058725), mRNA | 1% | 89% |
| CL179C4 | 1265 | Triticum aestivum chromosome 3B, genomic scaffold, cultivar Chinese Spring | 9% | 85% |
| CL180C1 | 3296 | Oryza sativa Indica Group cultivar Shuhui498 chromosome 12 sequence | 2% | 83% |
| CL181C4 | 1592 | Triticum aestivum chromosome 3B, genomic scaffold, cultivar Chinese Spring | 71% | 78% |
| CL182C10 | 1151 | No significant similarity found | 0% | 0% |
| CL182C10 | 1151 | Hordeum vulgare clone HV_Mba329-C16, complete sequence (Somewhat similar sequences (blastn)) | 4% | 91% |
| CL183C2 | 2818 | No significant similarity found | 0% | 0% |
| CL183C2 | 2818 | Hordeum vulgare clone HV_Mba112-L24, complete sequence | 45% | 65% |
| CL184C7 | 1318 | No significant similarity found | 0% | 0% |
| CL184C7 | 1318 | Triticum aestivum chromosome 3B, genomic scaffold, cultivar Chinese Spring | 38% | 75% |
| CL185C3 | 1416 | Avena strigosa beta-amyrin synthase (Sad1) and cytochrome P450 CYP51H10 (Sad2) genes, complete cds | 27% | 76% |
| CL186C3 | 801 | No significant similarity found | 0% | 0% |
| CL186C3 | 801 | Streptomyces albus strain BK3-25, complete genome (Somewhat similar sequences (blastn)) | 17% | 78% |
| CL187C2 | 996 | No significant similarity found | 0% | 0% |
| CL187C2 | 996 | Zebrafish DNA sequence from clone CH211-62D7, complete sequence (Somewhat similar sequences (blastn)) | 10% | 75% |
| CL188C3 | 1562 | No significant similarity found | 0% | 0% |
| CL188C3 | 1562 | Triticum aestivum chromosome 3B, genomic scaffold, cultivar Chinese Spring (Somewhat similar sequences (blastn)) | 13% | 75% |
| CL189C7 | 1458 | No significant similarity found | 0% | 0% |
| CL189C7 | 1458 | Ureaplasma diversum strain ATCC 49782 genome (Somewhat similar sequences (blastn)) | 3% | 87% |
| CL190C17 | 686 | No significant similarity found | 0% | 0% |
| CL190C17 | 686 | Avena strigosa beta-amyrin synthase (Sad1) and cytochrome P450 CYP51H10 (Sad2) genes, complete cds (Somewhat similar sequences (blastn)) | 96% | 67% |
| CL191C5 | 1324 | No significant similarity found | 0% | 0% |
| CL191C5 | 1324 | PREDICTED: Aegilops tauschii subsp. tauschii uncharacterized LOC109733731 (LOC109733731), transcript variant X3, mRNA (Somewhat similar sequences (blastn)) | 17% | 69% |
| CL192C8 | 1314 | Oryza sativa Indica Group cultivar Shuhui498 chromosome 3 sequence | 2% | 97% |
| CL193C1 | 1228 | No significant similarity found | 0% | 0% |
| CL193C1 | 1228 | Triticum aestivum chromosome 3B, genomic scaffold, cultivar Chinese Spring (Somewhat similar sequences (blastn)) | 33% | 76% |
| CL194C4 | 1785 | Brachypodium distachyon retrotransposon Gypsy CRBd1, complete sequence | 7% | 81% |
| CL195C8 | 910 | No significant similarity found | 0% | 0% |
| CL195C8 | 910 | Triticum aestivum chromosome 3B, genomic scaffold, cultivar Chinese Spring (Somewhat similar sequences (blastn)) | 45% | 72% |

Red: chloroplast cluster; blue: rDNA cluster
