# Supplementary material for: The repetitive DNA landscape in Avena (Poaceae): chromosome and genome evolution defined by major repeat classes in whole-genome sequence reads
Source: BMC Plant Biol. 2019 May 30;19:226. doi: 10.1186/s12870-019-1769-z (PMC6543597; doi:10.1186/s12870-019-1769-z)
Supplement: Supplementary file 17 — Table S5. Repetitive DNA composition of genomes of four Avena species. Genome portion (as percent) is listed for each species. Families and subfamilies of Transposable elements Class I (retrotransposons) and Class II (DNA transposons), and Tandem repeats are also given. (DOCX 22 kb) [file 12870_2019_1769_MOESM17_ESM.docx]

**Table S5.** Repetitive DNA composition of four *Avena* species genomes.

| Repeat type | Super family | Family | S312  *A. sativa* | B289  *A. brevis* | H299  *A. hirtula* | S315  *A. strigosa* |
| --- | --- | --- | --- | --- | --- | --- |
| Transposable Elements | | | | | | |
| Class I (Retrotransposons) | | | | | | |
| LTR | Gypsy | Ogre/Tat | 6.48% | 8.43% | 9.51% | 10.09% |
|  |  | Chromovirus | 12.61% | 10.89% | 11.78% | 11.18% |
|  |  | Athila | 6.43% | 6.61% | 4.43% | 3.71% |
|  |  | Unclassified Gypsy | 18.52% | 17.10% | 16.16% | 17.34% |
|  |  | Total Gypsy | 44.04% | 43.02% | 41.88% | 42.31% |
|  | Copia | Tork | 0.09% | 0.09% | 0.05% | 0.05% |
|  |  | TAR | 0.39% | 0.38% | 0.50% | 0.45% |
|  |  | Maximus/SIRE | 1.68% | 1.61% | 2.54% | 1.96% |
|  |  | Ivana/Oryco | 0.05% | 0.16% | 0.04% | 0.05% |
|  |  | Bianca | 0.51% | 0.06% | 0.10% | 0.09% |
|  |  | Angela | 10.19% | 11.04% | 9.46% | 10.60% |
|  |  | Alel-Retrofit | 0.75% | 0.75% | 1.00% | 0.81% |
|  |  | Unclassified Copia | 2.34% | 1.66% | 3.70% | 3.22% |
|  |  | Total Copia | 16.00% | 15.74% | 17.39% | 17.24% |
|  | PARA-RT | | 0.94% | 0.00% | 1.49% | 1.32% |
|  | Cassandra | | 0.06% | 0.10% | 0.04% | 0.06% |
|  | Unclassified LTR | | 0.02% | 0.03% | 0.00% | 0.00% |
|  | Non-Gypsy and non-Copia | | 1.02% | 0.13% | 1.53% | 1.38% |
| Total LTR- Retrotransposons | | | 61.37% | 58.19% | 61.25% | 61.35% |
| Non-LTR | LINE | L1 | 0.32% | 0.32% | 0.50% | 0.42% |
| Total Class I retrotransposons | | | 61.39% | 59.22% | 61.25% | 61.35% |
| Class II (DNA transposons)-Subclass 1 | | | | | | |
|  | PIF_Harbinger | | 0.03% | 0.04% | 0.05% | 0.03% |
|  | CMC-EnSpm | | 3.98% | 3.96% | 4.98% | 5.20% |
|  | MULE-MuDR | | 0.74% | 0.93% | 0.58% | 0.39% |
|  | TcMar-Stowaway | | 0.79% | 0.49% | 0.28% | 0.26% |
| Class II (DNA transposons)-Subclass II | | | | | | |
|  | Helitron | | 0.06% | 0.00% | 0.14% | 0.17% |
| Total DNA Class II transposons (Class II) | | | 5.59% | 5.43% | 6.03% | 6.05% |
| Total Transposable elements | | | 66.98% | 64.65% | 67.27% | 67.40% |
| Tandem Repeats | | | | | | |
| Satellite | | | 0.62% | 0.58% | 0.27% | 0.22% |
| Satellite telomere | | | 1.18% | 1.17% | 2.13% | 2.40% |
| rDNA | | | 0.64% | 0.73% | 0.44% | 0.45% |
| Total Tandem repeats | | | 2.44% | 2.48% | 2.82% | 3.06% |
| Unclassified (low complex, simple repeat, unknown) | | | 2.55% | 4.49% | 3.51% | 2.44% |
| Total Repetitive DNA | | | 71.96% | 71.62% | 73.60% | 72.90% |

For result comparation between subset genomes uploaded to the web server and whole genome data upload to the home server of Genepioneer Biotechnologies Co. Ltd, please refer to Zhou, 2018. The 25 clusters were selected for FISH analysis based on former analysis results in this paper.
