# Supplementary material for: The repetitive DNA landscape in Avena (Poaceae): chromosome and genome evolution defined by major repeat classes in whole-genome sequence reads
Source: BMC Plant Biol. 2019 May 30;19:226. doi: 10.1186/s12870-019-1769-z (PMC6543597; doi:10.1186/s12870-019-1769-z)
Supplement: Supplementary file 18 — Table S6. Oligonucleotides used to generate FISH probes. Primer pairs, annealing temperatures, the expected fragment sizes and lables for repeats identified from k-mer or RepeatExplorer analyses of four Avena species. (DOCX 23 kb) [file 12870_2019_1769_MOESM18_ESM.docx]

**Table S6.** Oligonucleotides used to generate FISH probes. Primer pairs, annealing temperatures, the expected fragment sizes and lables for repeats identified from k-mer or RepeatExplorer analyses of four *Avena* species.

| Repeat designation^1,2)^ | Oligonucleotide name | Sequence: 5’ to 3’ | Tm | Actual Tm | Expected product size (monomer; GC-content) | Label^3)^ |
| --- | --- | --- | --- | --- | --- | --- |
| S312_*Avena sativa* L. | | | | | | |
| - | 312CL82C115_263F | TGGTTCACTTACGCCATCCC | 67.0 | 57.1 | 636 (35.8%) | No PCR |
| - | 312CL82C115_898R | CTTGTACATCGCCCCAGGTT | 66.0 |  |  |  |
| - | 312CL83C5_31F | TTATAGGTGTGCCGATGCCC | 66.8 | 55.9 | 476  (353; 53.6%) | No PCR |
| - | 312CL83C5_506R | AACAAGTGAGGGCTACACCG | 64.0 |  |  |  |
| **As-T119** | **312CL119C15_226F** | **CATGTGATTTAGGCGCACCG** | **68.5** | **57.1** | **284**  **(338; 55.6%)** | **Dig** |
|  | **312CL119C15_509R** | **ATGTGTAGGCCCCATGTGTG** | **65.7** |  |  |  |
| - | 312CL125C11_115F | GATTGGCCAGCTCCTTCAGT | 65.6 | 55.0 | 899 (46.4%) | No PCR |
| - | 312CL125C11_1013R | AGCAGAGGGATCACCGTACT | 62.7 |  |  |  |
| As-R133 | 312CL133C2_1776F | AGGCGTCTAAGCATCATCGG | 66.3 | 53.1 | 342 (63.2%) | Dig |
|  | 312CL133C2_2117R | GATCTAGGAGCTGCGGTGTC | 63.9 |  |  |  |
| - | 312CL151C2_14F | TACGGGCCACAAAATCCTCC | 67.9 | 60.0 | 612  (116; 41.0%) | No PCR |
| - | 312CL151C2_625R | CACAGTTTTGGGGTTGCTCG | 67.7 |  |  |  |
| **As-T153** | **312CL153C32_82F** | **TTCACCCATCTCCATGTGGC** | **68.3** | **56.2** | **248**  **(193; 49.8%)** | **Dig** |
|  | **312CL153C32_330R** | **GTCAGAAAGCCCAGAGAGCC** | **65.3** |  |  |  |
| **As-T175** | **312CL175C6_251F** | **GGAACTTGCACGTGGACCTA** | **65.0** | **55.9** | **253**  **(186; 48.6%)** | **Dig** |
|  | **312CL175C6_503R** | **GGTTCAGACAACGCGCTTTT** | **66.3** |  |  |  |
| B289_*A*. *brevis* Roth | | | | | | |
| Ab-R18 | 289CL18C635_23F | ACTAATGGTCTGGCATTGCTG | 63.9 | 54.7 | 230 (36.8%) | Bio  Dig |
|  | 289CL18C635_252R | GTATCTTCTTAACCCTTGCTAGGT | 59.7 |  |  |  |
| Ab-R19 | 289CL19C395_48F | GAGTTCGAGGTGGTGCTCAA | 65.5 | 54.1 | 317 (57.4%) | Dig |
|  | 289CL19C395_364R | CCAGTACCCTCCGTTCAACC | 65.5 |  |  |  |
| - | 289CL93C5_145F | GTCACAACACGCTCCCACTA | 63.9 | 56.0 | 317  (357; 52.4%) | No PCR |
| - | 289CL93C5_627R | GCGGGCCTCAGATCATCTAC | 65.9 |  |  |  |
| **Ab-T105** | **289CL105C17_305F** | **AACATTCTAGGCCCCGGTTG** | **66.9** | **54.3** | **245**  **(352; 59.2%)** | **Bio**  **Dig** |
|  | **289CL105C17_549R** | **ATAGGGGTAGACGATCCGGG** | **65.9** |  |  |  |
| **Ab-R126** | 289CL126C28_445F | CTCCCCGACCTTTCCAACTC | 67.0 | 58.8 | 481 (45.7%) | Dig |
|  | 289CL126C28_925R | GCCTTCTCTCCGACATCGAG | 66.3 |  |  |  |
| **Ab-T145** | **289CL145C61_16F** | **ACCAGTGTGGCAGAAGTTGT** | **62.3** | **57.1** | **369**  **(198; 43.1%)** | **Dig** |
|  | **289CL145C61_384R** | **CATGCAATGTGGAAGCTGCT** | **66.3** |  |  |  |
| **Ab-T148** | **289CL148C17_281F** | **GGTGGATTGCCCCTAGATGG** | **67.5** | **54.3** | **353**  **(341; 56.4%)** | **Dig** |
|  | **289CL148C17_633R** | **GCAATCCACCTCCCTCTTGC** | **68.1** |  |  |  |
| **Ab- T159** | **289CL159C20_657F** | **TTCCACGCATGCTACACCAT** | **66.4** | **56.9** | **498**  **(750; 51.8%)** | **Dig** |
|  | **289CL159C20_1154R** | **AGGTGTTGGTTTGGGCTCAA** | **66.6** |  |  |  |
| **Ab-T166** | **289CL166C12_88F** | **AACCATGCATGTTTGGGCAC** | **67.9** | **57.1** | **397**  **(360; 47.4%)** | **Dig** |
|  | **289CL166C12_484R** | **CCGGGTTGGATCTTGTCACA** | **68.3** |  |  |  |
| - | 289CL187C4_21F | ATTCCTGGACGTGTAGGGGT | 64.4 | 56.0 | 331  (191; 48.6%) | No PCR |
| - | 289CL187C4_351F | GGTTCAGACAACGCGCTTTT | 66.3 |  |  |  |
| H299_*A*. *hirtula* Lag. | | | | | | |
| Ah-R31 | 299CL31C6_72F | TCGGTGACAAATGAGTGCGA | 68.0 | 57.1 | 338 (50.0%) | Dig |
|  | 299CL31C6_409R | CCGAGGACTGGAAGCAATGT | 66.4 |  |  |  |
| Ah-R52 | 299CL52C377_251F | ACTAGGGTCCAGAGATGCGT | 62.7 | 53.0 | 370 (57.8%) | Dig |
|  | 299CL52C377_620R | AAGATACTCGCCGTCGTTCC | 65.3 |  |  |  |
| **Ah-T118** | **299CL118C8_252F** | **CCCCGTAGCATTCTCCGTAC** | **65.5** | **53.0** | **610**  **(342; 55.7%)** | **Dig** |
|  | **299CL118C8_861R** | **CGTGTGGCCCTAGCATACAT** | **64.8** |  |  |  |
| **Ah-T125** | **299CL125C7_32F** | **GACCCAACCAAGCACAAACC** | **66.5** | **53.0** | **236**  **(358; 50.4%)** | **Dig** |
|  | **299CL125C7_267R** | **CGCATGTCTGTGAAGAGGGT** | **65.4** |  |  |  |
| -- | 299CL126C1_229F | CGATGCCAAGAGGGAGCTAG | 66.1 | 56.0 | 518  (650; 53.1%) | No PCR |
|  | 299CL126C1_746R | TTCCACGCATGCTACACCAT | 66.4 |  |  |  |
| S315_*A*. *strigosa* Schreb. | | | | | | |
| Ast-R87 | 315CL87C7_337F | CACTGAACAGAGGACCCCAC | 64.7 | 54.1 | 542 (54.6%) | Dig |
|  | 315CL87C7_878R | TGCTTGACGAAGGCGAAGAT | 67.1 |  |  |  |
| **Ast-T116** | **315CL116C17_235F** | **CCAAGGTTCCACCGGTCTA** | **64.9** | **58.5** | **608**  **(335; 56.9%)** | **Dig** |
|  | **315CL116C17_842R** | **TGCGTTGTTCAACCCTCGTA** | **66.5** |  |  |  |
| **Ast-T125** | **315CL125C12_303F** | **GCAATGGACGCTAGCATGTG** | **66.7** | **54.1** | **379**  **(355; 47.2%)** | **Dig** |
|  | **315CL125C12_681R** | **ACGTGCTAGGTCCATTGCAA** | **65.5** |  |  |  |
| Ast-R155 | 315CL155C10_349F | GAGGGAGTTGAAGTCGGTGG | 66.0 | 54.1 | 524 (48.3%) | Dig |
|  | 315CL155C10_872R | TTCCCCCATTCCCATCGTTG | 70.9 |  |  |  |
| Ast-R171 | 315CL171C1_64F | TCACAACCACCAGAAACCC | 63.6 | 59.5 | 632 (39.7%) | Dig |
|  | 315CL171C1_695R | AACGATTGCGATGCCTCAGA | 68.0 |  |  |  |
| Ast-R176 | 315CL176C4_478F | ACAAATACAGGCAGGGGAGC | 65.2 | 54.3 | 575 (47.0%) | Dig |
|  | 315CL176C4_1052R | CTTTGGGAGTGTTGTGCAGC | 65.8 |  |  |  |
| pAs120a  (AJ001922.1) | pAs120a_65F | CTGCCAACAACTGCTTGCTT | 59.9 | 51.3 | 289  (90; 38.8%) | Dig |
|  | pAs120a_354R | GAGGAAGTCGACCGAGGAAC | 59.8 |  |  |  |
| 45S rDNA^4)^ | pTa71_F | CGAACTGTGAAACTGCGAATGGC | 71.6 | 68.0 | 2700 (56%) | Dig |
|  | pTa71_R | TAGGAGCGACGGGCGGTGTG | 74.7 |  |  | Bio |
| 5S rDNA^5)^ | pTa794_M13_F | GTAAAACGACGGCCAGT | 58.7 | 55.0 | 410 (51.1%) | Bio |
|  | pTa794_M13_R | GGAAACAGCTATGACCATG | 58.1 |  |  |  |
| As_16mer43bp^6)^ | 312_16mer43bp | CATATGTGGTTTGTGGAAAGAACACACATGCAATGACTCTGGT | – | – | 43 (40.2%) | Bio |
| AF226603_45bp^6)^ | C_genome45bp  (Ananiev *et al.*, 2002) | GATCCACCAGTGTCATTGCATGTGTGTTGTTCTCATGAAACCACA | – | – | 45 (44.4%) | TET |

1. Repeat names included Species abbreviations: Ab, *Avena brevis*; Ah, *A. hirtula*; Ast, *A. strigosa*; As, *A. sativa* and repeat type. T, tandem; R, retrotransposon. Bold characters denoted tandem/satellite repeats.
2. Short hyphen indicate that no PCR product was generated.
3. Bio: biotin; Dig: digoxygenin; TET: Tetrachloro-Fluorescein Phosphoramidite.
4. Designed from the sequence of wheat clone pTa71 (Gerlach and Bedbrook, 1979).
5. Designed from the sequence of wheat clone pTa794 (Gerlach and Dyer, 1980).
6. Synthesied with label attached.
