# Supplementary material for: The repetitive DNA landscape in Avena (Poaceae): chromosome and genome evolution defined by major repeat classes in whole-genome sequence reads
Source: BMC Plant Biol. 2019 May 30;19:226. doi: 10.1186/s12870-019-1769-z (PMC6543597; doi:10.1186/s12870-019-1769-z)
Supplement: Supplementary file 19 — Table S7. RepeatExplorer characterization of selected repeat clusters of four Avena species. Characterization of repetitive fragments used as FISH probes from k-mer or RepeatExplorer analyses of Avena species. Genome proportion, domain hits, contig length and FISH figure references are listed. (DOCX 24 kb) [file 12870_2019_1769_MOESM19_ESM.docx]

**Table S7.** RepeatExplorer characterization of selected repeat clusters of four *Avena* species.

| Designation | Oligo name | Cluster read numbers | Cluster genome proportion | RepeatMasker annotation | Domain hits | Contig length (bp) | Read depth | Genome representation | FISH Figure(s) |
| --- | --- | --- | --- | --- | --- | --- | --- | --- | --- |
| **As-T119** | **312CL119C15** | 2956 | 0.15% | Satellite (925 hits, 23.20%) | Ty3-INT Ty3/gypsy  Ogre/Tat (1 hits, 0.0338%) | 5259 | 84.1 | 442182 | S3d |
| As-R133 | 312CL133C12 | 2010 | 0.10% | Simple-repeat (62 hits, 0.43%) | LINE-ENDO NA NA (1 hits, 0.05%) | 2155 | 21.6 | 46592 | S10b |
| **As-T153** | **312CL153C32**  **(Tandem)** | 1116 | 0.06% | RC.Helitron (2 hits, 0.03%) | DTA-CD1 NA NA (15 hits, 1.34%) | 2134 | 23.3 | 49714 | 4c |
| **As-T175** | **312CL175C6** | 554 | 0.03% | Satellite (34 hits, 43.20%) | Ty3-INT Ty3/gypsy Ogre/Tat (3 hits, 0.52%) | 1368 | 92.1 | 125964 | S3e |
| Ab-R18 | 289CL18C635 | 19232 | 0.99% | Low_cpmplexity (3325 hits, 5.55%) | PARA0RT NA NA (47 hits, 0.24%) | 1112 | 37.0 | 41141 | S3a |
| Ab-R19 | 289CL19C395 | 19197 | 0.99% | Low_cpmplexity (5686 hits, 4.28%) | Ty3-INT Ty3/gypsy chromovirus (8 hits, 0.04%) | 899 | 4.9 | 4434 | S3b |
| **Ab-T105** | **289CL105C17** | 3958 | 0.21% | Satellite (1342 hits, 25.30%) | DTN-CD2 NA NA (1 hits, 0.03%) | 3917 | 119.0 | 462205 | 5d |
| Ab-R126 | 289CL126C28 | 2183 | 0.11% | Low_cpmplexity (59 hits, 0.75%) | DTM-CD1 NA NA (1 hits, 0.05%) | 3318 | 56.1 | 186143 | S3b, S7a-S7f |
| **Ab-T145** | **289CL145C61**  **(Tandem)** | 1202 | 0.06% | Low_cpmplexity (3 hits, 0.07%) | DTM-CD1 NA NA (1 hits, 0.08%) | 2902 | 10.4 | 30193 | 4b |
| **Ab-T148** | **289CL148C17** | 1135 | 0.06% | Satellite (90 hits, 3.85%) | DTC-CD1 NA NA (1 hits, 0.09%) | 4444 | 59.7 | 265277 | 5a, S9a-S9f |
| **Ab-T159** | **289CL159C20** | 839 | 0.04% | Low_cpmplexity (15 hits, 0.18%) | LINE-ENDO NA NA (1 hits, 0.12%) | 1615 | 53.4 | 86186 | 5b, 5d |
| **Ab-T166** | **289CL166C12** | 724 | 0.04% | Satellite (25 hits, 1.53%) | DTH-CD1 NA NA (1 hits, 0.14%) | 1783 | 14.7 | 26174 | 5c, S6g-S6i |
| Ah-R31 | 299CL31C6 | 12857 | 0.77% | LTR.Gypsy (12485 hits, 77.8%) | Ty3-RT Tys/gypsy chromovirus (4291 hits, 33.4%) | 2412 | 777.9 | 1876966 | S8a-S8f |
| Ah-R52 | 299CL52C377 | 10288 | 0.62% | Simple-repeat (583 hits, 1.03%) | Ty3-GAG Ty3/gypsy chromovirus (39 hits, 0.38%) | 1647 | 13.2 | 21741 | S10a |
| **Ah-T118** | **299CL118C8** | 1841 | 0.11% | Satellite (865 hits, 25.5%) | DTC-CD1 NA NA (2 hits, 0.11%) | 4198 | 98.0 | 411410 | S3c |
| **Ah-T125** | **299CL125C7** | 1404 | 0.08% | Satellite (31 hits, 1.00%) | DTM-CD1 NA NA (2 hits, 0.14%) | 2412 | 11.4 | 275216 | 5e, S10e |
| Ast-R87 | 315CL87C7 | 3744 | 0.24% | Simple-repeat (295 hits, 1.32%) | LINE-RH NA NA (3 hits, 0.08%) | 1455 | 9.2 | 13433 | 4a |
| **Ast-T116** | **315CL116C17** | 1643 | 0.11% | Satellite (766 hits, 25.8%) | DTC-CD1 NA NA (1 hits, 0.06%) | 3467 | 102.0 | 353632 | 4e, S5a-S5f, S6d-S6f |
| **Ast-T125** | **315CL125C12** | 974 | 0.06% | Satellite (22 hits, 1.1%) | LINE-ENDO NA NA (1 hits, 0.10%) | 2791 | 68.7 | 191676 | S10f |
| Ast-R155 | 315CL155C10 | 437 | 0.03% | Simple-repeat (6 hits, 0.22%) | DTM-CD1 NA NA (1 hits, 0.23%) | 1115 | 14.4 | 16080 | S10c |
| Ast-R171 | 315CL171C1 | 298 | 0.02% | Simple-repeat (31 hits, 1.85%) | - | 614 | 5.0 | 3089 | 4d, S4a-S4f, S6a-S6c |
| Ast-R176 | 315CL176C4 | 234 | 0.02% | Simple-repeat (7 hits, 0.54%) | Ty1-INT Ty1/copia AleI/Retrofit (1 hits, 0.43%) | 1809 | 22.7 | 41062 | S10d |
| pAs120a | pAs120a align to 312CL17C141 | 18224 | 0.93% | LTR.Gypsy (2309 hits, 6.51%) | Ty3-INT Ty3/gypsy chromovirus (17 hits, 0.09%) | 2240 | 284.8 | 637979 | 4a, 4c-4e, 5b-5c, 5f, S3a, S3c, S4-s9 |
| **As_16mer43bp** | **312_16mer43bp align to 289CL8C154** | 24554 | 1.27% | LTR.Gypsy (100 hits, 0.09%) | Ty1-INT Ty1/copia Maximus/SIRE (74 hits, 0.30%) | 6997 | 26.2 | 183404 | 4b, S3d-S3f, S10a-S10f |
| **AF226603_45bp** | **C-genome45bp align to 289CL8C248** | 24554 | 1.27% | LTR.Gypsy (100 hits, 0.09%) | Ty1-INT Ty1/copia Maximus/SIRE (74 hits, 0.30%) | 4567 | 19.1 | 87034 | 4a, 4c-4e, S3a, S3c, S3f, S4-S9 |

Ab, *Avena brevis*; Ah, *A. hirtula*; Ast, *A. strigosa*; As, *A. sativa*. T, tandem; R, retrotransposon. Bold characters denoted tandem/satellite repeats.
