# Supplementary material for: The repetitive DNA landscape in Avena (Poaceae): chromosome and genome evolution defined by major repeat classes in whole-genome sequence reads
Source: BMC Plant Biol. 2019 May 30;19:226. doi: 10.1186/s12870-019-1769-z (PMC6543597; doi:10.1186/s12870-019-1769-z)
Supplement: Supplementary file 20 — Table S8. Genomic copy number and relative proportion of selected repeats of Avena species. PairedEnd fasta data were used to for “Map to Reference” tool in Geneious v.10.0.7 (Kearse et al., 2012). (DOCX 25 kb) [file 12870_2019_1769_MOESM20_ESM.docx]

**Table S8.** Genomic copy number and relative proportion of selected repeats of *Avena* species. PairedEnd fasta data were used to for “Map to Reference” tool in Geneious v.10.0.7 (Kearse et al., 2012).

(a) Repeats used for FISH analysis.

| Repeat designation | Lab number^1)^ | Copy number per genome | | | | Relative proportion within A genome species^2)^ | | | Genome specificity after FISH |
| --- | --- | --- | --- | --- | --- | --- | --- | --- | --- |
|  |  | S312_  *A. sativa*  (AACCDD) | H299_  *A. hirtula*  (AA) | B289_  *A. brevis*  (AA) | S315_  *A. strigosa*  (AA) | H299_  *A. hirtula*  (AA) | B289_  *A. brevis*  (AA) | S315_  *A. strigosa*  (AA) |  |
| As-T119 | 312CL119C15 | 424,926 | 620,107 | 292,288 | 832,560 | 35.74% | 17.16% | 47.10% | C |
| As-R133 | 312CL133C2 | 46,640 | 36,038 | 16,293 | 12,440 | 55.58% | 25.59% | 18.83% | ACD |
| As-T153 | 312CL153C32 | 107,820 | 24,847 | 59,239 | 0 | 29.17% | 70.83% | 0.00% | C |
| As-T175 | 312CL175C6 | 135,288 | 143,521 | 32,012 | 174,841 | 41.27% | 9.38% | 49.35% | C |
| Ab-R18 | 289CL18C635 | 355,982 | 285,651 | 198,632 | 251,635 | 38.87% | 27.52% | 33.61% | C |
| Ab-R19 | 289CL19C395 | 31,472 | 33,023 | 75,227 | 29,107 | 23.89% | 55.43% | 20.67% | C |
| Ab-T105 | 289CL105C17 | 367,725 | 838,491 | 255,860 | 490,113 | 53.07% | 16.49% | 30.44% | ACD |
| Ab-R126 | 289CL126C28 | 44,546 | 31,240 | 42,405 | 23,045 | 32.19% | 44.05% | 23.31% | AD |
| Ab-T145 | 289CL145C61 | 134,128 | 10,469 | 158,104 | 7,920 | 5.84% | 89.82% | 4.34% | C |
| Ab-T148 | 289CL148C17 | 149,274 | 142,931 | 273,207 | 128,011 | 26.14% | 50.88% | 22.98% | AD |
| Ab-T159 | 289CL159C20 | 100,237 | 104,228 | 52,265 | 91,338 | 42.18% | 21.54% | 36.28% | AD |
| Ab-T166 | 289CL166C12 | 863,319 | 109,471 | 351,203 | 82,294 | 19.98% | 65.28% | 14.74% | AD |
| Ah-R31 | 299CL31C6 | 722,967 | 545,493 | 448,550 | 546,289 | 35.46% | 29.69% | 34.85% | AD |
| Ah-R52 | 299CL52C377 | 35,189 | 27,407 | 17,144 | 34,111 | 34.98% | 22.28% | 42.73% | ACD |
| Ah-T118 | 299CL118C8 | 183,731 | 148,619 | 104,027 | 134,355 | 38.46% | 27.41% | 34.13% | C |
| Ah-T125 | 299CL125C7 | 870,357 | 245,382 | 581,483 | 679,977 | 16.31% | 39.35% | 44.35% | ACD |
| Ast-R87 | 315CL87C7 | 36,024 | 5,620 | 18,535 | 37,836 | 9.12% | 30.63% | 60.25% | C |
| Ast-T116 | 315CL116C17 | 160,509 | 204,152 | 78,146 | 127,971 | 49.87% | 19.44% | 30.68% | D |
| Ast-T125 | 315CL125C12 | 846,911 | 106,412 | 380,941 | 82,466 | 18.50% | 67.43% | 14.07% | ACD |
| Ast-R155 | 315CL155C10 | 57,714 | 20,947 | 32,233 | 24,687 | 26.85% | 42.08% | 31.06% | ACD |
| Ast-R171 | 315CL171C1 | 103,615 | 197,330 | 53,693 | 26,584 | 70.96% | 19.66% | 9.38% | D |
| Ast-R176 | 315CL176C4 | 60,004 | 19,289 | 31,562 | 17,207 | 28.23% | 47.05% | 24.72% | ACD |
| pAs120a^3)^ | pAs120a | 780,538 | 421,047 | 683,337 | 1,099,957 | 19.17% | 31.68% | 49.15% | A |
| As_16mer43bp | 312_16mer43bp^*)^ | 3,346,757 | 260 | 1,819,046 | 0 | 0.01% | 99.99% | 0.00% | C |
| AF226603_45bp^3)^ | C_genome45bp^*)^ | 755,507 | 689,064 | 683,437 | 993,882 | 29.19% | 29.48% | 41.33% | C |

(b) Additional repeats where FISH probes were not generated.

| Lab number^3)^ | S312_  *A. sativa*  (AACCDD) | H299_  *A. hirtula*  (AA) | B289_  *A. brevis*  (AA) | S315_  *A. strigosa*  (AA) |
| --- | --- | --- | --- | --- |
| 312CL82C115 | 102,384 | 2,414 | 52,302 | 3,255 |
| 312CL83C115 | 1,780,309 | 961,657 | 912,210 | 908,769 |
| 312CL125C11 | 62,417 | 1,510 | 32,697 | 30 |
| 312CL151C2 | 1,505,249 | 1,543,021 | 96,269 | 1,605,967 |
| 289CL93C5 | 1,668,984 | 1,110,061 | 481,122 | 895,806 |
| 289CL187C4 | 74,396 | 144,479 | 23,622 | 132,513 |
| 299CL126C1 | 113,989 | 116,217 | 53,896 | 78,644 |

1. Repeat names included species abbreviations: Ab, *Avena brevis*; Ah, *A. hirtula*; Ast, *A. strigosa*; As, *A. sativa* and repeat type. T, tandem; R, retrotransposon. Bold characters denoted tandem/satellite repeats
2. Probes generated by PCR except for those indicated by ^*)^, which were synthetic oligonucleotides.
3. Calculation method interpretation: Relative proportion = probe percentage in one *Avena* diploid species genome/probe percentage sum in *A. brevis*, *A. hirtula*, and *A. strigosa* genomes; Probe percentage in one diploid species genome = Probe fragment copy number/whole genome sequenced read number of the diploid species, Probe fragment copy number was determined as the probe fragment sequence mapping to the PairedEnd fasta data of particular *Avena* diploid species by “Map to Reference” tool in Geneious v.10.0.7 [38]; Probe percentage sum = Probe percentages in three *Avena* diploid genomes.
4. Repeats were identified in our analysis showing homology to known repeats [36,37].
5. No product was obtained after FISH.
