# Supplementary material for: The repetitive DNA landscape in Avena (Poaceae): chromosome and genome evolution defined by major repeat classes in whole-genome sequence reads
Source: BMC Plant Biol. 2019 May 30;19:226. doi: 10.1186/s12870-019-1769-z (PMC6543597; doi:10.1186/s12870-019-1769-z)
Supplement: Supplementary file 21 — Table S9. FISH signal distribution patterns on Avena sativa chromosomes using probes for selected repeats. Descriptions are based on analysis of 269 slides and 2353 metaphase (avg. 8 metaphases per slide) were analysed and chromosomes assigned to genomes C, A or D according to Sanz et al. (2010). Ten probes are C genome specific, one probe A genome specific, two probe D genome specific, five probes A and D genome specific and seven probes hybridized to all three genomes. (DOCX 25 kb) [file 12870_2019_1769_MOESM21_ESM.docx]

**Table S9.** FISH signal distribution patterns on *Avena sativa* chromosomes using probes for selected repeats. Descritptions are based on analysis of 269 slides and 2353 metaphase (avg. 8 metaphses per slide) were analysed and chromosomes assigned to genomes C, A or D according to Sanz et al. (2010).

| Repeat designation^1)^ | Oligonucleotide name | Labelled with^2)^ | Fig(s) | 1. *A. breivs* | 2. *A. atlantica* | 3. *A. strigosa* | 4. *A. wiestii* | 5. *A.longiglumis* | 6. *A.s­ativa* | 7. *A. eriantha* | 8. *A. hirtula* |
| --- | --- | --- | --- | --- | --- | --- | --- | --- | --- | --- | --- |
| **Direct labelled oligonucleotides** | | | | | | | | | | | |
| C-genome specific repeats | | | | | | | | | | | |
| **As_16mer43bp** | 312_16mer43bp | Bio | 4b, 5a, 5e, S3d, S3e, S3f, S10a-S10f | | Arms of 14 C-chromosomes, pericentromeres of 10 C-chromosomes, and distal regions of four C-chromosome long arms | | | | | | |
| **AF226603_45bp** | C_genome45bp | TET | 4a, c-e, 5b, 5c, 5f, S3a, S3c, S3f , S4-S9, S11c-S11e | | Dispersed signals along arms and pericentromeres of 14 C-chromosomes and on 10 A/D-chromosome long arms | | | | | | |
| **PCR amplified probes** | | | | | | | | | | | |
| C-genome specific repeats | | | | | | | | | | | |
| ●**Ab-R18** | 289CL18C635_23F | Dig | S3a |  | Dispersed on C-, A-, and D-chromosomes with high C-genome abundance |  | Dispersed on C-, A-, and D-chromosomes with high C-genome abundance |  |  |  |  |
|  | 289CL18C635_252R |  |  |  |  |  |  |  |  |  |  |
| ●**Ab-R19** | 289CL19C395_48F | Dig | S3b |  |  |  | Dispersed on C-, A-, and D-chromosomes with high C-genome abundance |  |  |  |  |
|  | 289CL19C395_364R |  |  |  |  |  |  |  |  |  |  |
| ●**Ast-R87** | 315CL87C7_337F | Dig | 4a | Strong signals on C-chromosomes and very weak signals on A/D- chromosomes | Strong signals on C- chromosomes and very weak signals on A/D-chromosomes | Strong signals on C- chromosomes and very weak signals on A/D- chromosomes |  | Strong signals on C- chromosomes and very weak signals on A/D- chromosomes |  |  | Strong signals on C-chromosomes and very weak signals on A/D- chromosomes |
|  | 315CL87C7_878R |  |  |  |  |  |  |  |  |  |  |
| ▲ **Ab-T145** | 289CL145C61_16F | Dig | 4b | Dispersed on C-, A-, and D-chromosomes with high C-genome abundance |  |  |  |  |  |  |  |
|  | 289CL145C61_384R |  |  |  |  |  |  |  |  |  |  |
| ▲**Ah-T118** | 299CL118C8_252F | Dig | S3c |  |  |  |  |  |  |  | Strong signals on C- chromosomes and very weak signals on A/D-chromosomes |
|  | 299CL118C8_861R |  |  |  |  |  |  |  |  |  |  |
| ▲**As-T119** | 312CL119C15_226F | Dig | S3d |  |  |  |  |  | Four C-chromosomes with double and multiple dots |  |  |
|  | 312CL119C15_509R |  |  |  |  |  |  |  |  |  |  |
| ▲**As-T153** | 312CL153C32_82F | Dig | 4c |  |  |  |  |  |  | Strong signals on C- chromosomes and very weak signals on A/D- chromosomes |  |
|  | 312CL153C32_330R |  |  |  |  |  |  |  |  |  |  |
| ▲**As-T175** | 312CL175C6_251F | Dig | S3e |  |  |  |  |  | Evenly dispersed on C-, A-, and D- chromosomes with distinctive dot signals on 14 C-chromosomes |  |  |
|  | 312CL175C6_503R |  |  |  |  |  |  |  |  |  |  |
| a-genome specific repeats | | | | | | | | | | | |
| ●**pAs120a** | 315_pAs120_65F | Bio | 4a, 4c-4e, 5b-5c, 5f, S3a, S3c, S4-s9 | Uniform A- chromosome signals | Uniform A- chromosome signals | Uniform A- chromosome signals | Uniform A- chromosome signals | Uniform A- chromosome signals |  |  | Uniform A- chromosome signals |
|  | 315_pAs120_354R |  |  |  |  |  |  |  |  |  |  |
| d-genome specific repeats | | | | | | | | | | | |
| ●**Ast-R171** | 315CL171C1_64F | Dig | 4d, S4a-S4f, S6a-S6c | Strong signals on D- chromosomes, several bands on A- chromosomes and minimal signals on C- chromosomes | Strong signals on D- chromosomes, several bands on A- chromosomes and minimal signals on C- chromosomes | Strong signals on D- chromosomes, several bands on A-chromosomes and minimal signals on C- chromosomes | Strong signals on D- chromosomes, several bands on A-chromosomes and minimal signals on C- chromosomes | Strong signals on D- chromosomes, several bands on A- chromosomes and minimal signals on C- chromosomes |  |  | Strong signals on D- chromosomes, several bands on A-chromosomes and minimal signals on C-chromosomes |
|  | 315CL171C1_695R |  |  |  |  |  |  |  |  |  |  |
| ▲**Ast-T116** | 315CL116C17_235F | Dig | 4e, S5a-S5f, S6d-S6f | Strong signals on D- chromosomes, several bands on A-chromosomes and minimal signals on C-chromosomes | Strong signals on D-Chromosomes, several bands on A-chromosomes and minimal signals on C-chromosomes | Strong signals on D-chromosomes, several bands on A-chromosomes and minimal signals on C-chromosomes | Strong signals on D-chromosomes, several bands on A-chromosomes and minimal signals on C-chromosomes | Strong signals on D-chromosomes, several bands on A-chromosomes and minimal signals on C-chromosomes |  |  | Strong signals on D-chromosomes, several bands on A-chromosomes and minimal signals on C-chromosomes |
|  | 315CL116C17_842R |  |  |  |  |  |  |  |  |  |  |
| A/D-genome specific repeats | | | | | | | | | | | |
| ●**Ab-R126** | 289CL126C28_445F | Dig | S3b, S7a-S7f | Dispersed signals on A/D-chromosomes with 10 unlabelled A/D-chromosome terminals | Dispersed signals on A/D-Chromosomes with 10 unlabelled A/D-chromosome terminals | Dispersed signals on A/D-chromosomes with 10 unlabelled A/D- chromosomes terminals | Dispersed signals on A/D-chromosomes with 10 unlabelled A/D-chromosome terminals | Dispersed signals on A/D- chromosomes with 10 unlabelled A/D-chromosome terminals |  |  | Dispersed signals on A/D- chromosomes with 10 unlabelled A/D- chromosome terminals |
|  | 289CL126C28_925R |  |  |  |  |  |  |  |  |  |  |
| ●**Ah-R31** | 299CL31C6_72F | Dig | S8a-s8f | Dispersed signals on A/D-chromosomes with 10 unlabelled A/D-chromosome terminals | Dispersed signals on A/D-chromosomes with 10 unlabelled A/D-chromosome terminals | Dispersed signals on A/D-chromosomes with 10 unlabelled A/D-chromosome terminals | Dispersed signals on A/D-chromosomes with 10 unlabelled A/D-chromosome terminals | Dispersed signals on A/D-chromosomes with 10 unlabelled A/D-chromosome terminals |  |  | Dispersed signals on A/D-chromosomes with 10 unlabelled A/D-chromosome terminals |
|  | 299CL31C6_409R |  |  |  |  |  |  |  |  |  |  |
| ▲**Ab-T148** | 289CL148C17_281F | Dig | 5a, S9a-S9f | Unevenly intercalary and subtelomeric dot (band) signals on A/D- chromosomes | Unevenly intercalary and subtelomeric dot (band) signals on A/D-chromosomes | Unevenly intercalary and subtelomeric dot (band) signals on A/D-chromosomes | Unevenly intercalary and subtelomeric dot (band) signals on A/D- chromosomes | Unevenly intercalary and subtelomeric dot (band) signals on A/D- chromosomes |  |  | Unevenly intercalary and subtelomeric dot (band) signals on A/D-chromosomes |
|  | 289CL148C17_633R |  |  |  |  |  |  |  |  |  |  |
| ▲**Ab-T159** | 289CL159C20_657F | Dig | 5b, 5d |  |  |  |  | Evenly dispersed and weak pericentromeric band signals on A/D- chromosomes |  |  | Evenly dispersed and weak pericentromeric band signals on A/D-chromosomes |
|  | 289CL159C20_1154R |  |  |  |  |  |  |  |  |  |  |
| ▲**Ab-T166** | 289CL166C12_88F | Dig | 5c, S6g-S6i | Evenly dispersed and weak pericentromeric band signals on A/D- chromosomes |  |  |  |  |  |  |  |
|  | 289CL166C12_484R |  |  |  |  |  |  |  |  |  |  |
| FISH probes labelling C-, A- and D- genomes | | | | | | | | | | | |
| ●**Ah-R52** | 299CL52C377_251F | Dig | S10a |  |  |  |  |  |  |  | Dispersed signals on C-, A- and D- chromosomes |
|  | 299CL52C377_620R |  |  |  |  |  |  |  |  |  |  |
| ●**As-R133** | 312CL133C2_1776F | Dig | S10b |  |  |  |  |  | Dispersed signals on C-, A- and D- chromosomes |  |  |
|  | 312CL133C2_2117R |  |  |  |  |  |  |  |  |  |  |
| ●**Ast-R155** | 315CL155C10_349F | Dig | S10c |  |  |  |  | Dispersed signals on C-, A- and D- chromosomes |  |  |  |
|  | 315CL155C10_872R |  |  |  |  |  |  |  |  |  |  |
| ●**Ast-R176** | 315CL176C4_478F | Dig | S10d |  |  |  | Dispersed signals on C-, A- and D- chromosomes |  |  |  |  |
|  | 315CL176C4_1052R |  |  |  |  |  |  |  |  |  |  |
| ▲**Ab-T105** | 289CL105C17_305F | Bio | 5d |  |  |  |  | Intercalary double dots on C-, A-, and D- chromosomes |  |  |  |
|  | 289CL105C17_549R |  |  |  |  |  |  |  |  |  |  |
| ▲**Ah-T125** | 299CL125C7_32F | Dig | 5e, S10e |  |  |  |  |  |  |  | Dispersed signals on C-, A- and D- chromosomes |
|  | 299CL125C7_267R |  |  |  |  |  |  |  |  |  |  |
| ▲**Ast-T125** | 315CL125C12_303F | Dig | S10f |  |  |  | Intercalary double dots on C-, A-, and D- chromosomes |  |  |  |  |
|  | 315CL125C12_681R |  |  |  |  |  |  |  |  |  |  |

1. Repeat names include species abbreviations: Ab, *Avena brevis*; Ah, *A. hirtula*; Ast, *A. strigosa*; As, *A. sativa*. T, tandem; R, retrotransposon. ●: Retroelement; ▲: Tandem repeat.
2. Bio: Biotin; Dig: Digoxigenin; TET: Tetrachloro-Fluorescein Phosphoramidite.
