# Supplementary material for: The repetitive DNA landscape in Avena (Poaceae): chromosome and genome evolution defined by major repeat classes in whole-genome sequence reads
Source: BMC Plant Biol. 2019 May 30;19:226. doi: 10.1186/s12870-019-1769-z (PMC6543597; doi:10.1186/s12870-019-1769-z)
Supplement: Supplementary file 22 — Table S10. The NCBI blast results of tandem repeats in Fig. 6b. Genome specificity, satellite family designation, monomer lengths and NCBI accession of twelve tandem repeats are listed. (DOCX 28 kb) [file 12870_2019_1769_MOESM22_ESM.docx]

Table S10. The NCBI blast results of tandem repeats in Figure 6b.

| Genomic specificity | Satellite family  designation | Repetitive DNA motif | Monomer length (bp) | Query coverage | Sort by identity | Accession | Satellite family | Reference |
| --- | --- | --- | --- | --- | --- | --- | --- | --- |
| *Avena* common ancestor tandem | Ab-T105 | 289CL105C17 | 352 | 91% | 68% | JX624130.1 | fpTR4 | Kopecký et al. [1] |
| *Avena* common ancestor tandem | Ah-T125 | 299CL125C7 | 358 | 61% | 77% | JX624130.1 | fpTR4 | Kopecký et al. [1] |
| *Avena* common ancestor tandem | Ast-T125 | 315CL125C12 | 355 | 15% | 78% | Z68782.1 | COM1 | Grebenstein et al. [2] |
| C-chromosome tandem | Ab-T145 | 289CL145C61 | 198 | 40% | 76% | AB039840.1 | fpTR4 | Kopecký et al. [1] |
| C-chromosome tandem | Ah-T118 | 299CL118C8 | 342 | 63% | 67% | JX624130.1 | fpTR4 | Kopecký et al. [1] |
| C-chromosome tandem | As-T119 | 312CL119C15 | 338 | 100% | 70% | JX624130.1 | fpTR4 | Kopecký et al. [1] |
| C-chromosome tandem | As-T153 | 312CL153C32 | 193 | 99% | 96% | AF226601.1 | AvsC-88-137 | Ananiev et al. [3] |
| C-chromosome tandem | As-T175 | 312CL175C6 | 186 | 100% | 75% | JX624130.1 | fpTR4 | Kopecký et al. [1] |
| A/D-chromosome tandem | Ab-T148 | 289CL148C17 | 341 | 86% | 79% | JX624130.1 | fpTR4 | Kopecký et al. [1] |
| A/D-chromosome tandem | Ab-T159 | 289CL159C20 | 750 | 100% | 91% | AF237540.1 | rlk6a2 pseudogene | Cheng et al. [4] |
| A/D-chromosome tandem | Ab-T166 | 289CL166C12 | 360 | 35% | 78% | Z68782.1 | fpTR4 | Kopecký et al. [1] |
| D-chromosome tandem | Ast-T116 | 315CL116C17 | 335 | 61% | 68% | JX624130.1 | fpTR4 | Kopecký et al. [1] |

Reference

1. Kopecký D, Martis M, Cíhalíková J, Hřobová E, Vrána J, Bartoš J, Kopecká J, Cattonaro F, Stočes Š, Novák P, et al*.* Flow sorting and sequencing meadow fescue chromosome 4F. Plant Physiol. 2013;163(3):1323–1337.
2. Grebenstein B, Grebenstein O, Sauer W, Hemleben V. Distribution and complex organization of satellite DNA sequences in Aveneae species. Genome. 1996;39(6):1045–1050.
3. Ananiev EV, Vales MI, Phillips RL, Rines HW. Isolation of A/D and C genome specific dispersed and clustered repetitive DNA sequences from *Avena* *sativa*. Genome. 2002;45(2):431–441.
4. Cheng DW, Armstrong KC, Drouin G, McElroy A, Fedak G, Molnar SD. Isolation and identification of Triticeae chromosome 1 receptor-like kinase genes (*Lrk10*) from diploid, tetraploid, and hexaploid species of the genus *Avena*. Genome. 2003;46(1):119–127.
